# Pyridylalkenolato-Stabilized Heavier Tetrylenes: Air-Stability Studies and Preliminary Coordination Chemistry

**DOI:** 10.1021/acs.inorgchem.5c05924

**Published:** 2026-05-18

**Authors:** Sergio García-Vega, Enrique Pérez-Carreño, Javier A. Cabeza, Pablo García-Álvarez

**Affiliations:** † Departamento de Química Orgánica e Inorgánica, Centro de Innovación en Química Avanzada ORFEO−CINQA, 16763 Universidad de Oviedo, Oviedo E-33071, Spain; ‡ Departamento de Química Física y Analítica, Universidad de Oviedo, Oviedo E-33071, Spain

## Abstract

The potential of 3,3,3-trifluoro-1-(2-pyridyl)­prop-1-en-2-ol (tfppOH) as a precursor to synthesize air-stable heavier tetrylenes (HTs), either free or coordinated, was examined. Thus, the stability toward air and water of the homoleptic HTs E­(tfppO)_2_ (E = Ge (**1**_**Ge**_), Sn (**1**_**Sn**_)), which were previously reported by Mathur and coworkers and claimed to be highly air-stable, was evaluated together with that of the novel heteroleptic chloro-HTs E­(tfppO)Cl (E = Ge (**2**_**Ge**_), Sn (**2**_**Sn**_)). These analyses have shown that all these compounds are susceptible to undergo hydrolysis at room temperature, albeit at very different rates for each HT. DFT calculations have been used to study the mechanism of these hydrolytic processes. Additionally, a preliminary study of the coordination chemistry of these HTs was conducted. The reactions of 0.5 equiv of [Ir_2_Cl_2_(μ-Cl)_2_(η^5^-Cp*)_2_] with **2**_**Ge**_ cleanly afforded the Ge–Ir complex [IrCl_2_(η^5^-Cp*)­{κ^1^ *Ge*-Ge­(tfppO)­Cl}] (**3**_**Ge**_); however, the analogous stannylene **2**_**Sn**_ led to a complex mixture, and the homoleptic derivatives **1**_**E**_ resulted in ligand degradation (formation of the HT-free complex [IrCl­(η^5^-Cp*)­{κ^2^ *N,O*-(tfppO)}] (**4**)). The Ge–Au complexes [AuCl­{κ^1^ *Ge*-Ge­(tfppO)_2_}] (**5**_**Ge**_) and [AuCl­{κ^1^ *Ge*-Ge­(tfppO)_2_}_2_] (**6**_**Ge**_) could be prepared by reacting **1**_**Ge**_ with [AuCl­(tht)] (tht = tetrahydrothiophene); however, only the precipitation of dark-purple solids was observed using **1**_**Sn**_. On the other hand, both **1**_**E**_ were capable of rendering the diHT-Ag analogous compounds [Ag­{κ^1^ *E*-E­(tfppO)_2_}_2_]­OTf (E = Ge (**7**_**Ge**_), Sn (**7**_**Sn**_)) in their reactions with AgOTf. The air-stability of all these complexes in solution was proven to be similar or lower (for **7**_**Sn**_) than that of the corresponding metal-free HTs.

## Graphical abstract

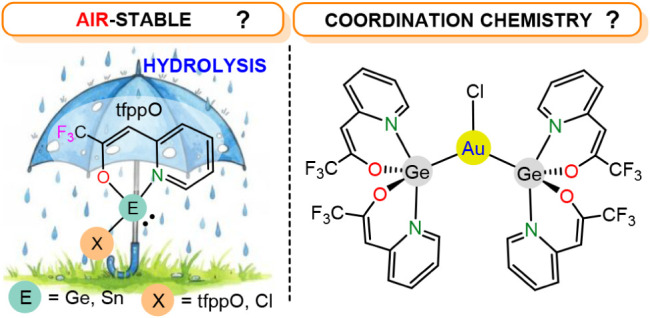

## 1 Introduction

Transition metal (M) complexes of heavier tetrylenes (HTs),^1^ which are heavier carbene analogues, are increasingly attracting the interest of the chemical community as a result of their intrinsic nonconventional characteristics (metal–ligand cooperation scenarios are common due to their ambiphilic character)^2^ and because they have already been successfully involved in important bond activation and catalytic processes.^3,4^ However, the low stability toward air and moisture of the metal-free species and also of their complexes has prevented a wider use of these compounds in catalysis, which has been rarely examined in the absence of an inert atmosphere^5^ or using protic^6^ solvents. Therefore, further studies aimed at expanding the applicability of HTs or HT-M complexes without protective reaction conditions are desirable. In this regard, several HTs have been reported to have a certain air-stability,^7–13^ property which was simply mentioned when describing their synthesis,^7^ or was playing a central role of the corresponding report (see Figure 1).^8–13^ Very important are the contributions of Nagendran et al.,^8–10^ who have managed to prepare, relying on the electronic stabilization and steric protection provided by 6-membered-*N*,*N*-chelating dipyrromethene (**I**),^8^ aza-dipyrrinate (**II**)^9^ and bis­(dipyrromethene) (**III**)^10^ fragments, a large series of germylenes (the first one in 2014)^8a^ showing remarkable air- and water-stability, which was proven by systematic experiments. Such a stability allowed, for example, the evaluation of the antiproliferative effects of the hydroxygermylene of type **I** in culture medium, which was comparable to that of cisplatin in human cancer cells with minimal cytotoxicity.^8b^ Very recently, in 2024, Su, Xu and coworkers have reported the air-stable chlorostannylene **IV**, also stabilized by a very bulky dipyrromethene moiety.^11^ Highlighting the importance of steric protection, the related dipyrromethene-stabilized chlorostannylene reported by Kobayashi, Kawashima et al.,^7c^ but equipped with much smaller protecting groups (Me instead of 2,4,6-triisopropylphenyl (Tripp), as in **IV**), has been described as relatively stable in air only in the solid state. On the other hand, other HTs not equipped with bulky groups, but doubly stabilized by two 6-membered-*N*,*O*-chelating β-heteroarylalkenolate fragments, such as **V** and **VI** (**1**_**Ge**_, **1**_**Sn**_ and **1**_**Pb**_), reported by Mathur et al. in 2011 ^12^ and 2014,^13^ respectively, have also been claimed as highly air- and moisture-stable. According to the authors,^13^ the push–pull effect to the tetrel atom (an electron-donating pyridine and an electron-withdrawing CF_3_ group), which ensures strong chelation of the *N*,*O*-fragment, is responsible for the high stability observed for **VI** (the same can be attributed to **V**). These homoleptic HTs, relying in their monomeric nature, are volatile, which makes them promising precursors for chemical vapor deposition (CVD) processes (this was proven for **V**, which allowed the production of highly crystalline SnO_2_ nanowires).^12^ However, it is noteworthy mentioning that, contrasting with the work of Nagendran,^8–10^ the claimed high air- and moisture-stability of **V** and **VI** was not demonstrated in their corresponding reports.

**Figure 1:**
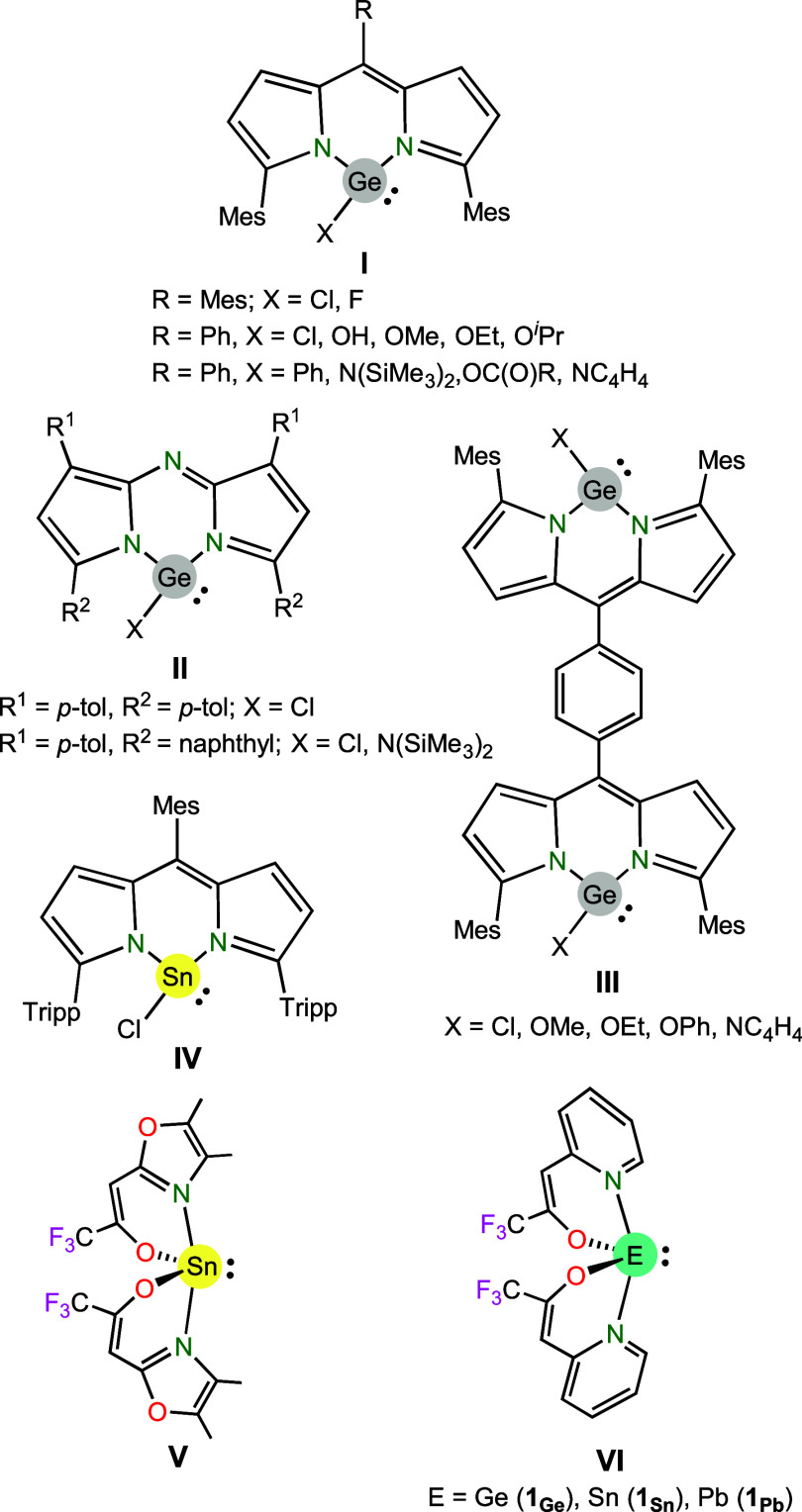
Representative examples of HTs reported to be air-stable.

Prompted by the few reports^8–10^ aimed at systematically evaluating the air-stability of HT derivatives, in this work, we have further examined the real potential of the pyridylalkenolato fragment (tfppO), already utilized for the preparation of HTs **VI (1**_**Ge**_, **1**_**Sn**_ and **1**_**Pb**_),^13^ to render oxygen- and/or water-stable HTs, either free or coordinated. This particular fragment was also chosen because the usefulness of HTs equipped with 6-membered-*N*,*O*-chelating monoanionic fragments as ligands has been barely explored.^14,15^

## 2 Results and Discussion

First, a heteroleptic version of Mathuŕs **1**_**Ge**_ and **1**_**Sn**_ was attempted. Thus, a two-step procedure consisting of deprotonation of tfppOH with Li­(hmds) followed by a reaction with GeCl_2_(1,4-dioxane) or SnCl_2_ was carried out, leading to the quantitative formation of the novel heteroleptic derivatives E­(tfppO)­Cl (E = Ge (**2**_**Ge**_), Sn (**2**_**Sn**_)), which could be isolated in 84% and 79% yield, respectively (Scheme 1). Curiously, **2**_**Ge**_ and **2**_**Sn**_ show a markedly different solubility in organic solvents, the stannylene being much less soluble.

**Scheme 1:**
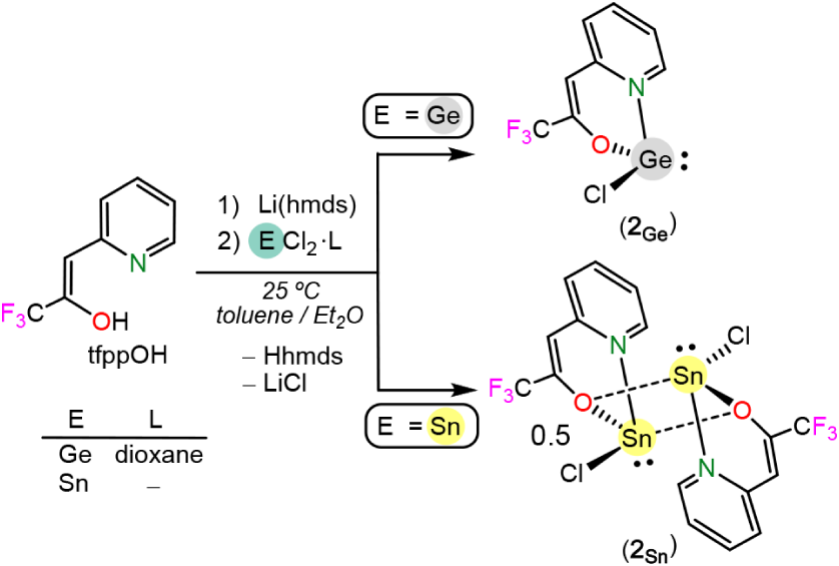
Synthesis of **2**_**Ge**_ and **2**_**Sn**_

The NMR data of **2**_**Ge**_ and **2**_**Sn**_ in CDCl_3_ (Figures S1 and S3, respectively) are quite similar. Their ^1^H and ^13^C­{^1^H}­NMR spectra show, in addition to the signals associated with the pyridine ring, a more shielded signal corresponding to the vinylic C–H group (δ_H_ = 6.33 (**2**_**Ge**_), 6.10 (**2**_**Sn**_) ppm; δ_C_ = 98.9 (**2**_**Ge**_), 98.2 (**2**_**Sn**_) ppm). Regarding the signals of the OCCF_3_ moiety, very distinctive quartets appear at δ_C_ = 150.9 ppm (*J* = 35 Hz; O*C*CF_3_) and 119.6 ppm (*J* = 277 Hz; OC*C*F_3_) for germylene **2**_**Ge**_, which could not be observed for **2**_**Sn**_, possibly due to its much lower solubility, even after prolonged NMR acquisition times.

The solid-state structures of **2**_**Ge**_ and **2**_**Sn**_ were established by single-crystal X-ray diffraction (SCXRD) (Figures 2 and 3, respectively). In both cases, the tetrel atom is bound to the Cl atom and *N*,*O*-chelated by the pyridylalkenolato fragment, forming a six-membered ENCCCO ring. However, while **2**_**Ge**_ is monomeric, stannylene **2**_**Sn**_ forms a centrosymmetric dimer featuring a Sn_2_O_2_ four-membered planar ring due to the existence of long but significant intermolecular Sn···O interactions (2.766 Å). Note that the sum of the vdW radii of oxygen and tin is 3.69 Å.^16^ This dimerization via E···O intermolecular interactions, which has been reported for related chloro-HTs stabilized by *N*,*O*-chelating fragments,^17^ is more common for tin as a consequence of its greater Lewis acidity. To this regard, for example, Khrustalev et al. have reported that compounds E­(OCH_2_CH_2_NMe_2_)­(hmds) are monomeric for E = Ge, but dimeric for E = Sn,^18^ ^a^ and our group has described that the HTs E­(hmds)­(bqfam) (E = Ge, Sn; bqfam = N,N′*-bis*(quinol-8-yl)­formamidinate) form κ^3^ *E,N,N*′*-*bqfam metal complexes in which a quinolyl fragment is pendant for E = Ge but attached to the tetrel atom for the tin complex.^18b^ The geometries of the tetrel atoms of **2**_**Ge**_ and of the monomeric unit of **2**_**Sn**_ are very similar and can be described as very distorted three-coordinate tetrahedral (angles around E are 92(3)° for **2**_**Ge**_ and 85(4)° for **2**_**Sn**_) with one position occupied by the lone electron pair. The E-X (X = Cl, O, N) bond distances of **2**_**E**_ are in the range reported for the other related crystallographically characterized chloro-TPs stabilized by chelating *N*,*O*-monoanionic (6-membered ring) fragments, being, as expected, 0.1–0.2 Å longer for tin.^19^

**Figure 2:**
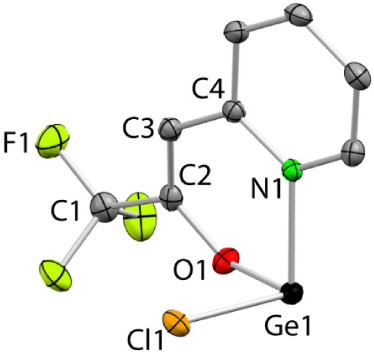
SCXRD molecular structure of **2_Ge_** (only one of the two analogous molecules found in the asymmetric unit is shown; H atoms have been omitted for clarity). Selected interatomic distances (Å) and angles (°): Ge1–Cl1 2.2989(8), Ge1–N1 2.060(2), Ge1–O1 1.876(2), C1–C2 1.515(4), C2–O1 1.325(4), C2–C3 1.342(4), C3–C4 1.446(4), C4–N1 1.354(4); O1–Ge1–N1 90.2(1), O1–Ge1–Cl1 94.70(7), N1–Ge1–Cl1 90.71(7), C2–O1–Ge1 123.8(2).

**Figure 3:**
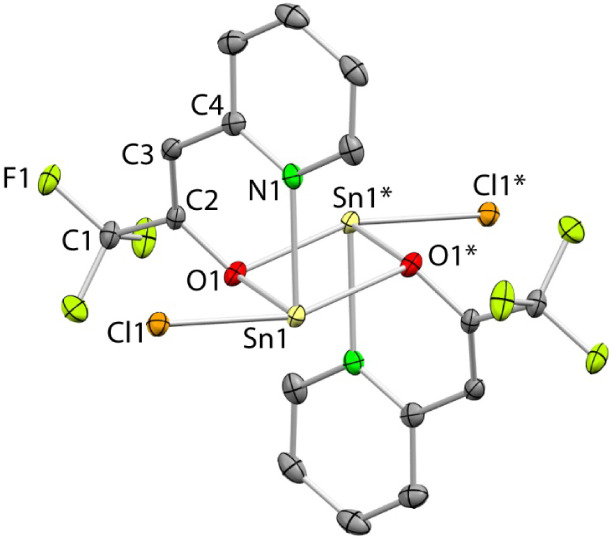
SCXRD molecular structure of **2_Sn_** (the asymmetric unit only shows half of the centrosymmetric molecule; H atoms have been omitted for clarity). Selected interatomic distances (Å) and angles (°): Sn1–Cl1 2.5447(5), Sn1–N1 2.328(2), Sn1–O1 2.095(1), Sn1···O1* 2.766, C1–C2 1.507(3), C2–O1 1.329(2), C2–C3 1.347(3), C3–C4 1.455(3), C4–N1 1.354(3); O1–Sn1–N1 80.41(6), O1–Sn1–Cl1 89.79(4), N1–Sn1–Cl1 86.13(4), C2–O1–Sn1 122.9(1), Cl1–Sn1···O1* 158.14, O1–Sn1···O1* 70.27, Sn1–O1···Sn1* 109.73.

To gather information regarding the aggregation state of **2**_**Sn**_ in solution, the ^1^H DOSY NMR spectra of equally concentrated solutions of **2**_**Ge**_ and **2**_**Sn**_ were recorded in CDCl_3_ (Figure S5). These spectra showed that both compounds have very similar diffusion coefficients (*D* = 1.41 × 10^–9^ (**2**_**Ge**_) and 1.38 × 10^–9^ (**2**_**Sn**_) m^2^ s^–1^). From these data, it can be inferred^20^ that **2**_**Ge**_ and **2**_**Sn**_ have approximately the same size, indicating that the dimeric structure of **2**_**Sn**_ is not retained in solution. Probably, the higher tendency of **2**_**Sn**_ to dimerize (the dimeric form is expected to be less soluble), explains its marked lower solubility compared to that of **2**_**Ge**_.

With **2**_**E**_ (E = Ge, Sn) in hand, we decided to check their air-stability and that of the homoleptic HTs **1**_**E**_ (note that, as previously mentioned, the air-stability claimed for **1**_**E**_ had not been demonstrated).^13^ Thus, the ^1^H NMR spectra of solutions of **1**_**E**_ and **2**_**E**_, both in C_6_D_6_ or CDCl_3_, were recorded under argon and after standing in air for 1 day.^21^ In all cases, hydrolytic degradation occurred (tfppOH was detected), albeit at very different rates for each HT (Figure S33). This degradation was in general faster in CDCl_3_ than in C_6_D_6_, most probably due to the higher solubility of water in the chlorinated solvent.^22^ Interestingly, while the homoleptic germylene **1**_**Ge**_ seemed to be less hydrolysis-stable than stannylene **1**_**Sn**_, the opposite trend was observed for the heteroleptic derivatives, germylene **2**_**Ge**_ appearing to be more resistant to hydrolysis than stannylene **2**_**Sn**_. After 3 days under air, only the signals of tfppOH were observed in the corresponding NMR spectra (Figure S34), except for chlorogermylene **2**_**Ge**_, which showed a 1:1 **2**_**Ge**_:tfppOH ratio. It is important to mention that other degradation processes (i.e., oxidation) cannot be discarded; however, hydrolytic decomposition seems to be particularly relevant, since similar ^1^H NMR spectra were recorded for solutions of **1**_**E**_ and **2**_**E**_ in CDCl_3_ after the addition of one equiv of water.^23^ In order to acquire a more accurate picture of the degradation observed for **1**_**E**_ and **2**_**E**_, the air-stability tests in CDCl_3_ were also carried out in the presence of an internal standard. Figure 4 is a graphical representation of this study, indicating the approximate percentages of remaining HT and of the formed hydrolytic product tfppOH (considering the maximum amount possible in each case) contained in solutions of **1**_**E**_ and **2**_**E**_ in CDCl_3_ that were left standing under atmospheric air (left column) for 1 week.^24^ In order to ascertain the role played by atmospheric oxygen in these reactions, the NMR measurements were also performed under dry air (right column).

**Figure 4:**
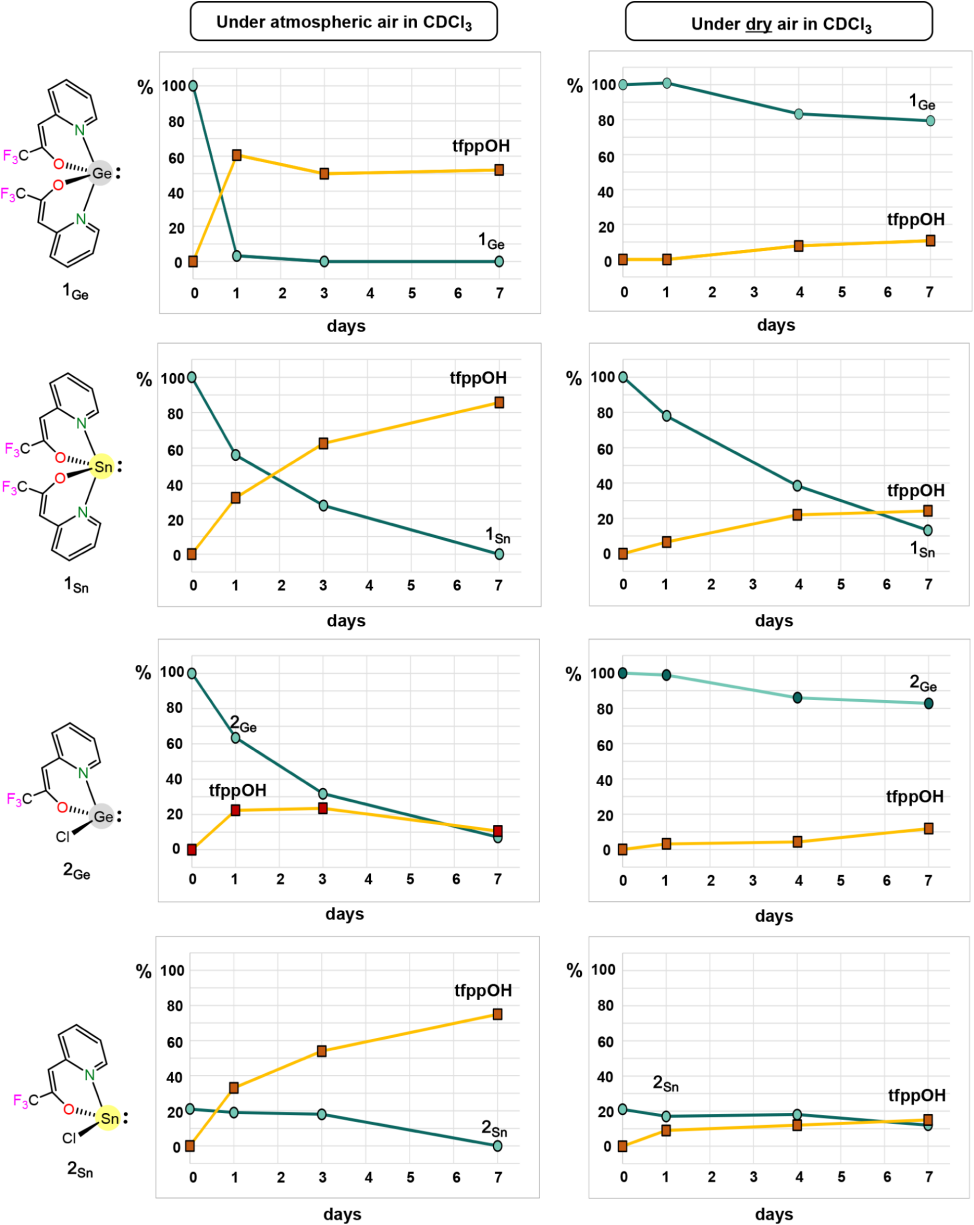
Stability of CDCl_3_ solutions of **1_E_** and **2_E_** under atmospheric air (left) and under dry air (right).

Regarding the homoleptic derivatives under atmospheric air, the germylene disappeared much faster than the stannylene (3% of **1**_**Ge**_ vs 56% of **1**_**Sn**_ after 1 day), the latter undergoing a slow degradation during the analyzed one-week period. Curiously, while almost all the possible hydrolytic product tfppOH was produced when no traces of **1**_**Sn**_ were left (86% of tfppOH after 7 days), roughly half of it was detected when **1**_**Ge**_ completely disappeared (52% of tfppOH after 3 days). On the other hand, under dry air, both HTs slowly degraded during the one-week period, being, in this case, the germylene much more stable than the stannylene (79% of **1**_**Ge**_ vs 13% of **1**_**Sn**_ after 7 days). For **1**_**Ge**_, in addition to this 21% degradation, a 11% of tfppOH was also detected (the dry air must contain traces of moisture), while for **1**_**Sn**_, its almost total disappearance (87% degradation) only rendered a 24% of tfppOH. All these data indicate that *(i)* **1**_**E**_ degradation in air is mainly due to moisture, leading approximately to the hydrolysis of one (for **1**_**Ge**_) or two (for **1**_**Sn**_) of their two tfppO fragments (formation of tfppOH and undetected insoluble species such as Sn­(OH)_2_ or those derived from, for example, Ge­(tfppO)­OH; the formation of solid was also observed during the tests) and *(ii)* **1**_**Sn**_ can additionally undergo degradation due to atmospheric oxygen.

Considering the heteroleptic germylene **2**_**Ge**_ under atmospheric air, a slow degradation was observed (64% remaining after 1 day) during the one-week period. However, in this case, no large amounts of tfppOH were detected, quantifying a maximum of 24% after 3 days, which decreased with time. In fact, when **2**_**Ge**_ almost completely disappeared (7% remaining after 7 days), only 11% of tffppOH was present in solution. While these results seem to indicate that the degradation of **2**_**Ge**_ in air is mainly due to atmospheric oxygen, the data obtained using dry air demonstrate again that moisture is the main cause of the germylene degradation in atmospheric air (**2**_**Ge**_ proved to be quite stable under dry air; 83% detected after 7 days). The faster hydrolytic cleavage of the Cl group, with concomitant HCl release, might explain the absence of large amounts of tfppOH and even its decrease with time during **2**_**Ge**_ degradation (an independent experiment proved that tfppOH reacts with HCl in CDCl_3_ leading to an insoluble solid; see Figure S56). Finally, regarding the stability of the heterolytic stannylene **2**_**Sn**_, less accurate data were obtained due to its low solubility (only *ca*. 20% of the HT is initially in solution), which cannot be reliably compared with those obtained for **1**_**E**_ and **2**_**Ge**_. In fact, from this data, it cannot be clearly concluded that **2**_**Ge**_ is more resistant to hydrolysis than **2**_**Sn**_, as previously suggested. However, the atmospheric air vs dry air data comparison clearly shows that the **2**_**Sn**_ degradation in air is mainly due to moisture (75% and 15% of tfppOH detected after 7 days under atmospheric air and dry-air, respectively).

Additionally, for comparison purposes, the air stability of solid samples of **1**_**E**_ and **2**_**E**_ was evaluated by ^1^H NMR in the presence of an internal standard.^24^ After 1 day, the percentages of remaining HT and of formed tfppOH (considering the maximum amount possible in each case) were: 75/8% (**1**_**Ge**_/tfppOH), 76/7% (**1**_**Sn**_/tfppOH), 31/16% (**2**_**Ge**_/tfppOH) and 27/38% (dissolved **2**_**Sn**_/tfppOH). These data indicate that a higher air-stability in the solid state is only clearly observed for the homoleptic tetrylenes **1**_**E**_, particularly for **1**_**Ge**_.

Having in mind that the experimental stability tests indicate that moisture is the main cause of **1**_**E**_ and **2**_**E**_ degradation in air, DFT calculations were performed modeling the possible initial stages of their reactions with water leading to the formation of the detected hydrolytic product tfppOH. Figure 5 displays the calculated^22^ mechanism for the transformation of **1**_**E**_ + H_2_O (**R-E**) into E­(tfppO)­OH + tfppOH (**P-E**) and a graphical representation of the evolution of relevant bond distances. It should be noted that in the initial hydrolysis products **P-E**, the tfppOH fragment is the ketone-pyridinium tautomer. This form is more stable than the enol-pyridine due to effective O2···H4 and O3···H3 intermolecular hydrogen bonding stabilizations (distances of ca. 1.6 Å). The first step of the reaction, which is rate determining, involves a near concerted reaction of a water O–H bond with one E–O moiety (**TS1-E**). Specifically, O3 coordinates E, while H3 binds one of the tfppO oxygen atoms (O2), leading to O2–E bond scission and forming intermediate **I1-E**. Interestingly, while the formation of **I1-E** leads to the separation of the tetrel atom from the protonated O atom (O2) of the hydrolyzed tfppO fragment, its pyridine N2 atom still remains attached to E [(Ge–N2 distances = 2.336 Å (**R-Ge**) and 2.409 Å (**I1-Ge**); Sn–N2 = 2.458 Å (**R-Sn**) and 2.489 Å (**I1-Sn**)]. The evolution of intermediates **I1-E** toward **P-E** (one step for E = Ge and three steps for E = Sn) implies the separation of the hydrolyzed tfppOH and E­(tfppO)­OH units and the final isomerization to the pyridinium tautomeric form of tfppOH. Note that along the entire process, the E-O1 and E-N1 bond distances, which involve the unhydrolyzed tfppO chelating unit, exhibit similar values. Overall, the modeled hydrolysis can occur at room temperature for both derivatives, particularly for **1**_**Sn**_ (ΔG_**TS1‑E**_ = 14.0 (Ge), 11.5 (Sn) kcal mol^–1^); however, while the process is slightly exergonic for **1**_**Ge**_ (ΔG_**P‑Ge**_ = −2.4 kcal mol^–1^) it is slightly endergonic for **1**_**Sn**_ (ΔG_**P‑Sn**_ = 4.8 kcal mol^–1^). Therefore, the subsequent evolution of E­(tfppO)­OH (which is not detected under experimental conditions possibly due to the formation of insoluble products resulting from, for example, further hydrolysis, condensation and/or oxidation reactions)^25^ should, necessarily for tin, importantly contribute to the favorable thermodynamics of the complete hydrolysis reaction. Despite of the modeled process having a higher kinetic barrier for germanium, its exergonic nature might explain the experimental lower stability of **1**_**Ge**_ toward hydrolysis.

**Figure 5:**
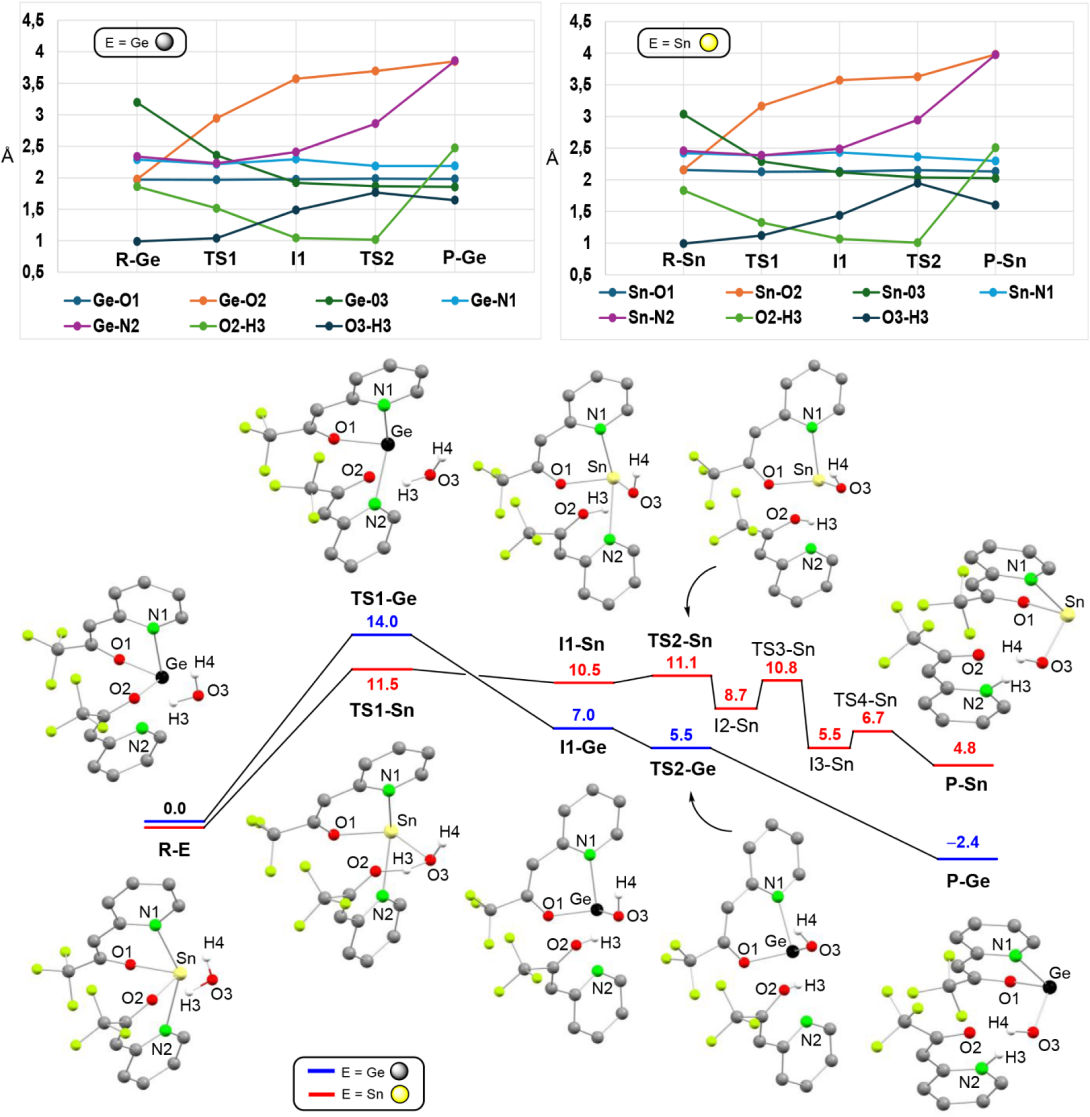
DFT-optimized structures (BP86-D3/SDD­(Ge,Sn)) of the stationary points and relative energy profiles of the transformations of **1_E_** + H_2_O (**R-E**) into E­(tfppO)­OH + tfppOH (**P-E**), for E = Ge and Sn. Gibbs energies (CPCM-CDCl_3_, kcal mol^–1^) are relative to those of **R-E**. The evolution of relevant bond distances (Å) is shown in the upper part of the figure. For clarity, (a) only the H atoms derived from the water molecule are shown and (b) the optimized structures of **I2-Sn**, **TS3-Sn**, **I3-Sn** and **TS4-Sn** and the Gibbs energies (CPCM-C_6_D_6_, kcal mol^–1^),^22^ are not shown here but are depicted in Figure S64.

For the hydrolysis of **2**_**E**_, two possibilities were calculated for the initial reaction with water, leading either to the hydrolytic cleavage of the tfppO fragment or of the Cl group. Figure 6 displays the calculated^22^ transformation of **2**_**E**_ + H_2_O (**R′-E**) into EClOH + tfppOH (**P′-E**) and a graphical representation of the evolution of relevant bond-distances. The reaction processes are similar to those described above for the homoleptic derivatives (Figure 5), however, in this case: *(i)* the number of steps is the same for both tetrels, *(ii)* the rate-determining step, which has also a higher activation barrier for germanium, is ca. 7–6 kcal mol^–1^ higher in energy (ΔG_**TS1′‑E**_ = 21.4 (Ge), 17.1 (Sn) kcal mol^–1^) and *(iii)* the processes for both derivatives, particularly for tin, are endergonic (ΔG_**P′‑E**_ = 6.5 (Ge), 14.0 (Sn) kcal mol^–1^). Regarding the hydrolytic cleavage of the Cl group of **2**_**E**_, Figure 7 displays the calculated^22^ transformation of **2**_**E**_ + H_2_O (**R″-E**) into E­(tfppO)­OH + HCl (**P″-E**), which proceeds via a single step process for both tetrels. For the reaction to occur, the near concerted reaction of a water O–H bond is in this case with the E–Cl group. The process releases HCl, which maintains a clear hydrogen bonding interaction with the hydroxy group of the formed E­(tfppO)­OH product formed (O2···H2 distances are ca. 1.5 Å). Similarly to the previous mechanism, the modeled hydrolysis is endergonic for both derivatives, (ΔG_**P″‑E**_ = 4.6 (Ge), 9.9 (Sn) kcal mol^–1^) and has a higher activation barrier for germanium (ΔG_**TS1″‑E**_ = 16.9 (Ge), 15.6 (Sn) kcal mol^–1^).

**Figure 6:**
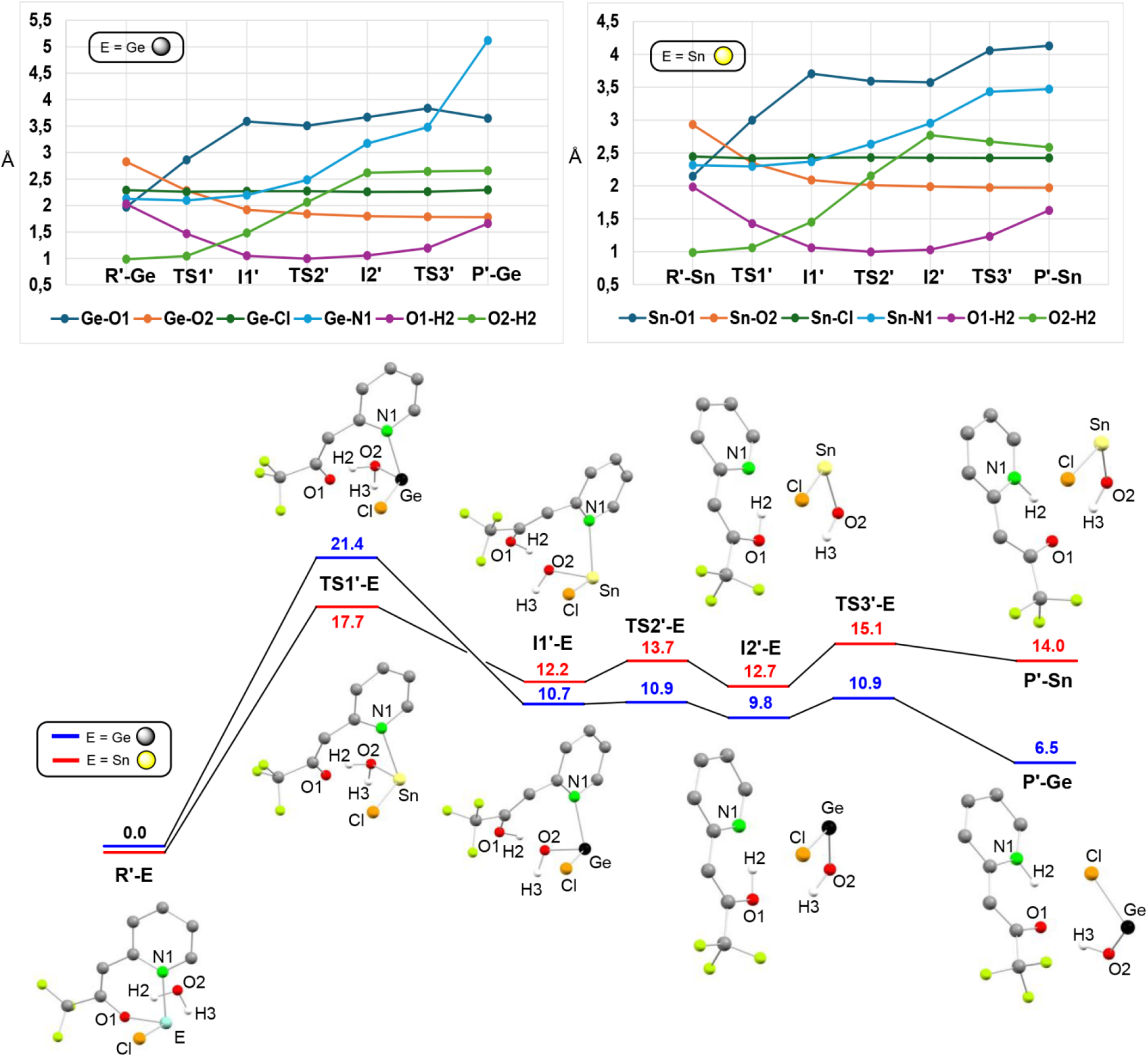
DFT-optimized structures (BP86-D3/SDD­(Ge,Sn)) of the stationary points and relative energy profiles of the first steps of the transformations of **2_E_** + H_2_O (**R′-E**) into ECl­(OH) + tfppOH (**P′-E**), for E = Ge and Sn. Gibbs energies (CPCM-CDCl_3_, kcal mol^–1^) are relative to those of **R′-E**. The evolution of relevant bond distances (Å) is shown in the upper part of the figure. For clarity, (a) only the H atoms derived from the water molecule are shown and (b) the optimized structures of **TS2′-E** and **TS3′-E** and the Gibbs energies (CPCM-C_6_D_6_, kcal mol^–1^),^22^ are not shown here but are depicted in Figure S65.

**Figure 7:**
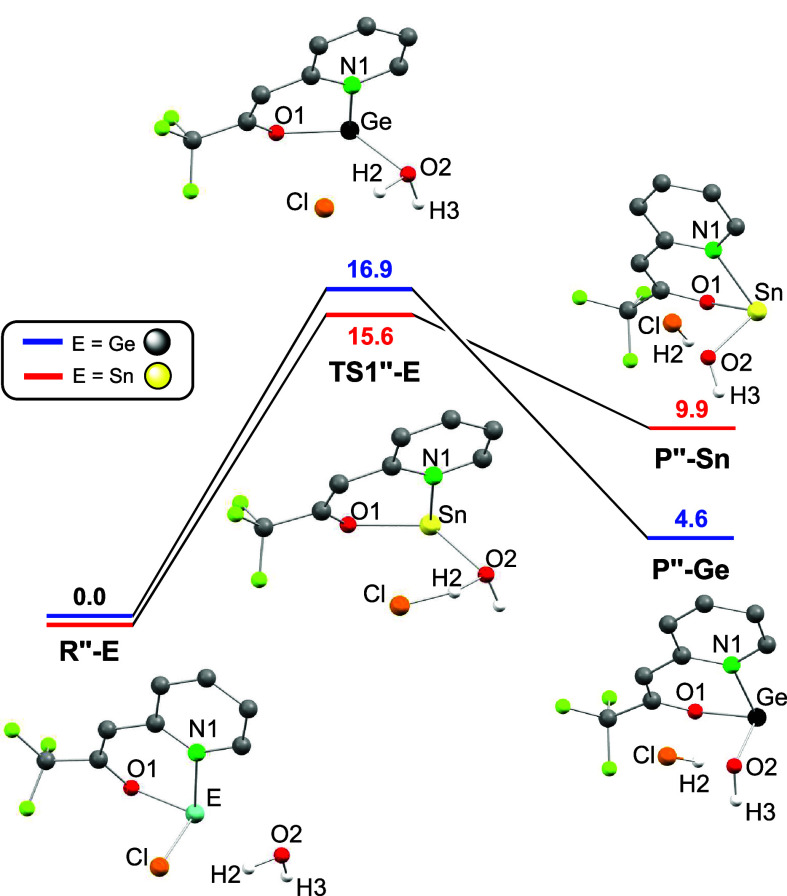
DFT-optimized structures (BP86-D3/SDD­(Ge,Sn)) of the stationary points and relative energy profiles of the transformations of **2_E_** **+** H_2_O (**R″-E**) into E­(tfppO)­OH + HCl (**P″-E**), for E = Ge and Sn. Gibbs energies (CPCM-CDCl_3_, kcal mol^–1^) are relative to those of **R″-E**. For clarity, (a) only the H atoms derived from the water molecule are shown and (b) the Gibbs energies (CPCM-C_6_D_6_, kcal mol^–1^),^22^ are not shown here but are depicted in Figure S66.

In summary, a thermodynamically favorable hydrolysis of the heteroleptic compounds **2**_**E**_ must also rely on the subsequent evolution of the EClOH or E­(tfppO)­OH products, which could form via lower activation barrier processes for tin. According to the theoretical data, the hydrolytic cleavage of the Cl–E bond is the most probable initial pathway occurring since: *(i)* the Cl-hydrolysis (Figure 7) has a lower activation barrier than the tfppO-hydrolysis (Figure 6), *(ii)* the controlled Cl-hydrolysis of other chloro-HTs to render hydroxo-HTs has been reported,^26^ and *(iii)* the further hydrolysis of the E­(tfppO)­OH products proposed formed will render the detected tfppOH. According to the experimental results (see Figure 4), a more predominant Cl–E bond hydrolysis pathway is only evident for **2**_**Ge**_, since, very differently to **2**_**Sn**_, no large amounts of tfppOH were detected during the germylene degradation (maximum of 24% after 3 days). The higher difference between the Cl- and tfppO-hydrolysis activation barriers for E = Ge (ca. 4 kcal mol^–1^) in comparison with that for E = Sn (ca. 2 kcal mol^–1^) might be in line with this experimental observation.

A preliminary study on the coordination chemistry of HTs **1**_**E**_ and **2**_**E**_ was carried out aiming at: *(i)* evaluating the usefulness as ligands of HTs equipped with 6-membered-*N*,*O*-chelating monoanionic fragments, which is, as previously mentioned, barely explored,^14,15^ and *(ii)* improving their air/water stability upon coordination (some HT-Ms have been described to be air-stable^5,6,27^ featuring HTs which are very possibly^28^ or reported to be^29^ air-unstable).

First, the reactivity of **1**_**E**_ and **2**_**E**_ with 0.5 equiv of [Ir_2_Cl_2_(μ-Cl)_2_(η^5^-Cp*)_2_], previously used to produce the fairly air-stable Ge–Ir complex [IrCl_2_(η^5^-Cp*)­{κ^1^ *Ge*-Ge­(^*t*^Bu_2_bzam)^*t*^Bu}] (^*t*^Bu_2_bzam = *N*,*N′* bis­(*tert*-butyl)­benzamidinate),^6^ was investigated (Scheme 2). While the reaction with heteroleptic **2**_**Ge**_ cleanly afforded the Ge–Ir complex [IrCl_2_(η^5^-Cp*)­{κ^1^ *Ge*-Ge­(tfppO)­Cl}] (**3**_**Ge**_), which could be isolated in quantitative yield, the analogous stannylene **2**_**Sn**_ led to a complex mixture of unidentified species. On the other hand, the reaction with the homoleptic derivative **1**_**Ge**_ led to a complex mixture that slowly (after 3 days at 25 °C and 30 h at 60 °C) evolved to the formation of a major product, which according to^1^H NMR analysis (Figure S8), featured an unexpected 1:1 ratio of tfppO and Cp* fragments. This major species was later identified as the as the HT-free complex [IrCl­(η^5^-Cp*)­{κ^2^ *N,O*-(tfppO)}] (**4**), which could be independently prepared (85% isolated yield) by direct reaction of deprotonated tfppOH (using Li­(hmds)) with 0.5 equiv of [Ir_2_Cl_2_(μ-Cl)_2_(η^5^-Cp*)_2_] (Scheme 3). Complex **4** was also formed in the reaction of the same iridium precursor with the homoleptic stannylene **1**_**Sn**_ (see Figure S9). Such a ligand degradation by transmetalation of the HT chelating fragment to a metal complex has been previously reported.^19a,30^

**Scheme 2:**
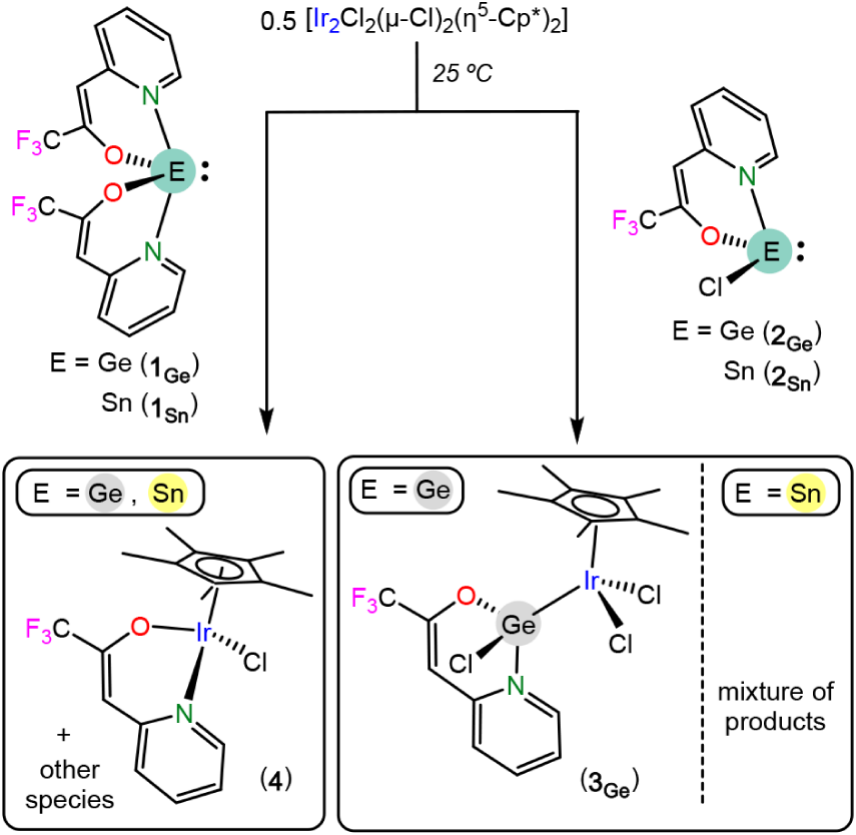
Reactions of **1**_**E**_ and **2**_**E**_ with [Ir_2_Cl_2_(μ-Cl)_2_(η^5^-Cp*)_2_]

**Scheme 3:**
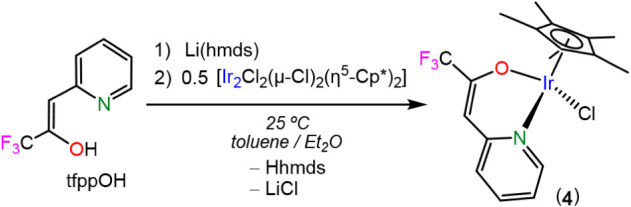
Synthesis of Complex **4**

The solid state structure of **3**_**Ge**_ was established by SCXRD (Figure 8). It has the characteristic “three-legged piano-stool” geometry of [IrCl_2_(η^5^-Cp)­L] (L = two electron-donor ligand) complexes. The germylene is *κGe*-coordinated to the metal center in such a way that the plane defined by the GeNCCCO ring roughly bisects the ClIrCl angle. The Ir–Ge bond distance, 2.3436(7) Å, is within the range of Ir–Ge distances (2.284–2.435 Å) found for the other crystallographically characterized iridium complexes featuring terminal neutral germylene ligand.^31^ The NMR data of **3**_**Ge**_ in CDCl_3_ (Figure S6) show the expected signals for the tfppO and η^5^-Cp* fragments in a 1:1 ratio, being the η^5^-Cp* ligand rotating freely in solution (singlet at δ­(^1^H) 1.85 ppm). It is noteworthy that, having in mind the importance of the [IrCl_2_(η^5^-CpR)­L] (L = two electron-donor ligand, CpR = any cyclopentadienyl ring) family of complexes,^32^ very few [IrCl_2_(η^5^-Cp)­(HT)] compounds have been previously reported.^33^

**Figure 8:**
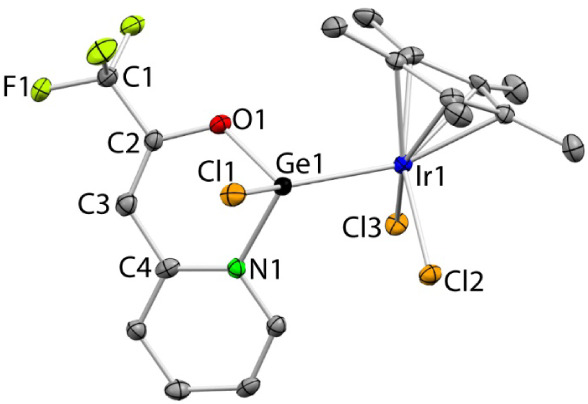
SCXRD molecular structure of **3_Ge_** (only one of the two analogous molecules found in the asymmetric unit is shown; H atoms have been omitted for clarity). Selected interatomic distances (Å) and angles (°): Ge1–Cl1 2.202(2), Ge1–N1 1.977(5), Ge1–O1 1.827(4), Ge1–Ir1 2.3436(7), C1–C2 1.504(9), C2–O1 1.315(8), C2–C3 1.34(1), C3–C4 1.459(9), C4–N1 1.355(8), Ir1–Cl2 2.431(2), Ir1–Cl3 2.411(2); O1–Ge1–N1 93.6(2), O1–Ge1–Cl1 97.4(2), N1–Ge1–Cl1 97.2(2), O1–Ge1–Ir1 117.7(1), N1–Ge1–Ir1 121.6(2), Cl1–Ge1–Ir1 122.96(6), C2–O1–Ge1 121.6(4).

The air-stability of complex **3**_**Ge**_ was checked. ^1^H NMR spectra of a solution of **3**_**Ge**_ in CDCl_3_ were recorded under argon and after standing in air for 1 day (see Figure S57). A considerable, but not complete, hydrolytic degradation (tfppOH and tppfO signals of **3**_**Ge**_ appeared in ca. 1:1 ratio) was observed. Thus, complexation of **2**_**Ge**_ to iridium does not improve the air-stability of this HT (tested under similar conditions, Figure S28).

The tfppO moiety of **4** is assumed to be *N*,*O*-chelated to the metal center, since many other [IrCl­(η^5^-Cp*)­{κ^2^ *N,O*-L)}] (L = monoanionic OC_3_N ligand) complexes have been reported.^34^

Then, further attempts to incorporate **1**_**E**_ to other metal complexes, in particular, using the GXI precursors [AuCl­(tht)] and AgOTf, were carried out. The reactions of **1**_**E**_ with the gold complex led to very different results depending on the E atom (Scheme 4). Thus, while the Ge–Au complex [AuCl­{κ^1^ *Ge*-Ge­(tfppO)_2_}] (**5**_**Ge**_) was the main product (isolated as pale brown solid in 91% yield) of the 1:1 (molar ratio) reaction with **1**_**Ge**_, only the precipitation of an untractable dark-purple solid, possibly related with the formation of Au(0), was observed using **1**_**Sn**_. This solid, albeit in much lesser extent, was also observed during the formation of **5**_**Ge**_ or after long periods of the complex standing in solution. Then, the reactions of [AuCl­(tht)] with 2 equiv of **1**_**E**_ were carried out, since we had previously learned that the reactions of Lappert́s HTs E­(hmds)_2_ (E = Ge, Sn) with [AuCl­(tht)] led to more stable complexes when fitted with two of such ligands.^35^ This different stoichiometry allowed the clean formation (isolated as a yellowish solid in 96% yield) of the disubstituted Ge_2_–Au complex [AuCl­{κ^1^ *Ge*-Ge­(tfppO)_2_}_2_] (**6**_**Ge**_) when **1**_**Ge**_ was used as ligand, however, the precipitation of dark-purple solids were again observed for the reaction with **1**_**Sn**_. Therefore, it seems that tin­(II) species are more prone to reduce gold­(I) to gold(0) than germanium­(II) species.

**Scheme 4:**
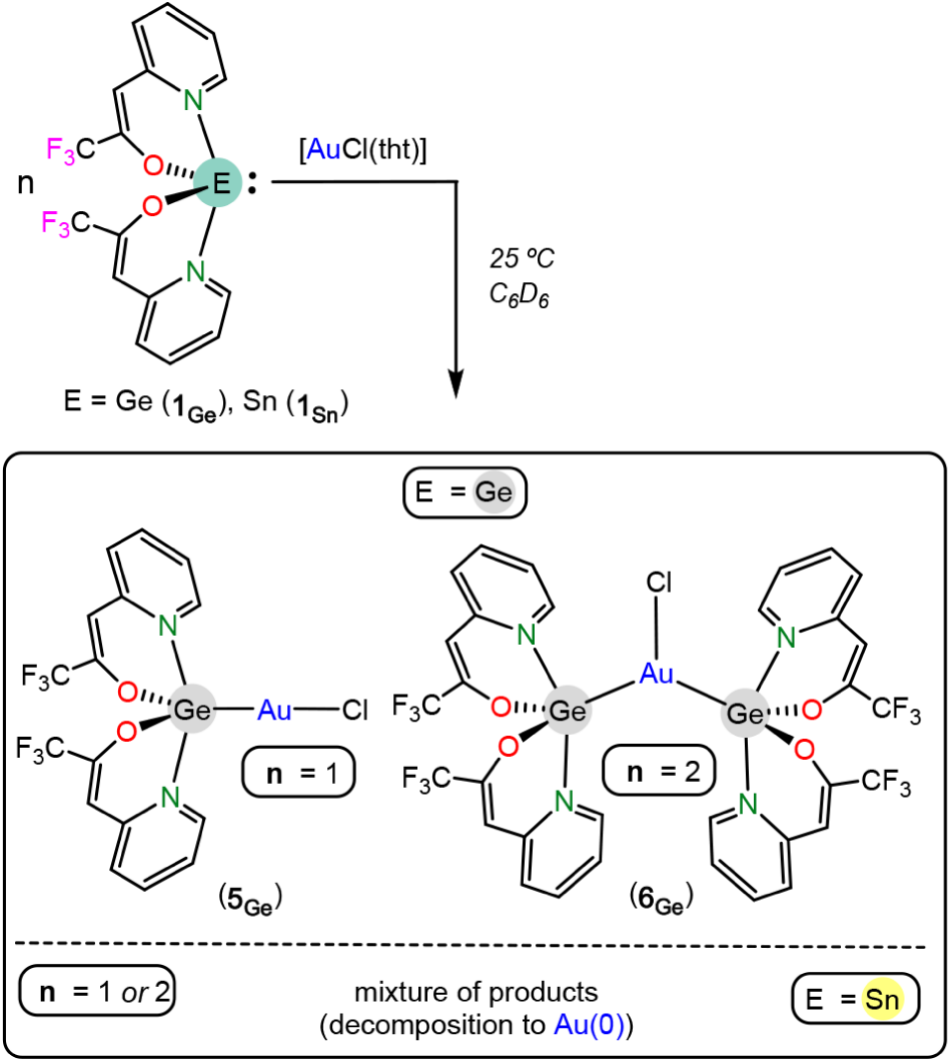
Reactions of **1**_**E**_ with [AuCl­(tht)]

The NMR data of **5**_**Ge**_ in C_6_D_6_ (Figure S11) indicate that both tfppO fragments are equivalent in solution (only one set of signals is observed). These signals are similar to those corresponding to **1**_**Ge**_^13^ (only chemical shift differences were observed). Regarding **6**_**Ge**_, its NMR data in C_6_D_6_/CDCl_3_ (Figure S13) indicate that both germylenes, and also both tfppO fragments of each germylene, are equivalent in solution, since only one set of signals is observed for the four tfppO fragments. Therefore, both ligands of **6**_**Ge**_ must be symmetry-related in solution or must be involved in a fluxional process. The coordination of a second germylene does not exert a significant change in the tfppO signals chemical shifts, which are very similar for both **5**_**Ge**_ and **6**_**Ge**_ (the greatest deviation corresponds to the signal corresponding to the *ortho*-CH proton of the pyridine fragment, which appears at δ 8.73 ppm for **5**_**Ge**_ and at 8.52 ppm for **6**_**Ge**_).

The SCXRD structure of **5**_**Ge**_ (Figure 9) shows a typical linear gold­(I) complex (Cl1–Au1–Ge1 176.12(4)°) equipped with a *κGe*-germylene and a chlorido as ligands. Regarding the germylene, its conformation is very similar to that reported for free **1**_**Ge**_,^13^ in a way the germanium atom is in the center of a distorted trigonal bipyramid (calculated τ_5_ value^36^ for **5**_**Ge**_ is 1.35), in which the pyridinic nitrogens are in axial positions and the oxygens and gold atom (or lone pair for **1**_**Ge**_) are in equatorial positions. The Au–Ge bond distance, 2.3293(7) Å, lies in the lowest limit of the range of Au–Ge distances (2.33–2.48 Å) found for other crystallographically characterized gold complexes featuring terminal neutral germylene ligands.^37^ It is noteworthy that none^38^ of these known complexes feature a five-coordinate germanium atom (all of them are three- or four-coordinate), such as that of **5**_**Ge**_. It is important that the SCXRD structure of **5**_**Ge**_ was measured from a crystal precipitated from a solution of the disubstituted complex **6**_**Ge**_, which hints to the existence in solution at room temperature of the dissociation equilibrium **6**_**Ge**_ ↔ **5**_**Ge**_ + **1**_**Ge**._ The presence of such an equilibrium, which was further investigated by DFT calculations, was also evidenced when studying the air-stability of **6**_**Ge**_ (vide supra).

**Figure 9:**
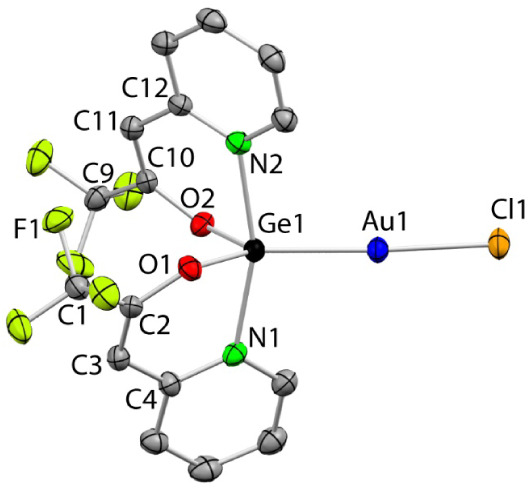
SCXRD molecular structure of **5_Ge_** (H atoms have been omitted for clarity). Selected interatomic distances (Å) and angles (°): Ge1–N1 2.187(5), Ge1–O1 1.828(4), Ge1–N2 2.142(5), Ge1–O2 1.828(4), Ge1–Au1 2.3293(7), C1–C2 1.515(8), C2–O1 1.331(6), C2–C3 1.340(9), C3–C4 1.473(8), C4–N1 1.339(8), C9–C10 1.502(9), C10–O2 1.335(7), C10–C11 1.344(8), C11–C12 1.446(9), C12–N2 1.351(8), Au1–Cl1 2.287(2); O2–Ge1–O1 105.8(2), O2–Ge1–N2 87.9(2), O1–Ge1–N2 82.9(2), O2–Ge1–N1 81.0(2), O1–Ge1–N1 86.8(2), N2–Ge1–N1 162.3(2), O2–Ge1–Au1 130.4(1), O1–Ge1–Au1 123.7(1), N2–Ge1–Au1 100.5­(1), N1–Ge1–Au1 973(1), Ge1–Au1–Cl1 176.12(4).

The structure of **6**_**Ge**_ could not be established by SCXRD. Therefore, the thermodynamic stability of the DFT-optimized structures (Figures S67–S69) of several reasonable isomeric configurations of **6**_**Ge**_ was compared with that of **5**_**Ge**_ + **1**_**Ge**_ (Figure 10). The proposed isomers for **6**_**Ge**_ are *(i)* a neutral tricoordinated gold complex (**6**_**AuClGe2**_) featuring two germylenes and a chloride as ligands (several related [AuClP_2_] (P = monodentate phosphane) compounds have been reported, for example, [AuCl­(PPh_3_)_2_]),^39^ *(ii)* a neutral dicoordinated gold complex (**6**_**AuGeClGe**_) equipped with one germylene and one chloro-germanate, as a result of the insertion of one of the germanium atoms into the Au–Cl bond (insertion reactions of HTs into M-halogen bonds, including Au–Cl bonds,^35,40^ are very common) and *(iii)* the chloride salt of a cationic dicoordinated gold complex $\left(6_{\mathrm{AuGe2+C}l^{‐}}\right)$ featuring two germylenes (several related [AuP_2_]Cl (P = phosphane) ionic compounds are known, for example, [Au­(PCy_3_)_2_]­Cl).^41^ This study indicated that only the neutral tricoordinated gold isomer **6**_**AuClGe2**_ is more stable (−6.2 kcal mol^–1^) than **5**_**Ge**_ + **1**_**Ge**_, (**6**_**AuGeClGe**_ and $6_{\mathrm{AuGe2+C}l^{‐}}$ are, respectively, 6.7 kcal mol^–1^ and 40.8 kcal mol^–1^ less stable than **5**_**Ge**_ + **1**_**Ge**_). Therefore, **6**_**Ge**_ features two germylenes and an Au–Cl bond (isomer **6**_**AuClGe2**_), as shown in Scheme 4. This contrasts with the configuration known for the other few gold complexes equipped with two heavy G14-donor ligands of any type and a chloride group, which invariantly feature the chlorine atom attached to one of the G14-ligands and not to the gold atom.^35,40^ The transformation of **5**_**Ge**_ + **1**_**Ge**_ into **6**_**Ge**_ was modeled by DFT calculations (Figure S70), showing the initial formation of an encounter complex **6-I1** (3.6 kcal mol^–1^ more stable than the reactants), which evolves to the final product **6**_**Ge**_ through an essentially barrierless process (ΔG_**6‑TS1**_ = 2.9 kcal mol^–1^). This, in addition to the small energy difference between **5**_**Ge**_ + **1**_**Ge**_ and **6**_**Ge**_ (6.2 kcal mol^–1^), is in agreement with the proposed existence at room temperature of the dissociation equilibrium **6**_**Ge**_ ↔ **5**_**Ge**_ + **1**_**Ge**_, which can easily lead to **5**_**Ge**_ if **1**_**Ge**_ is consumed by other means (*e.g*., decomposition).

**Figure 10:**
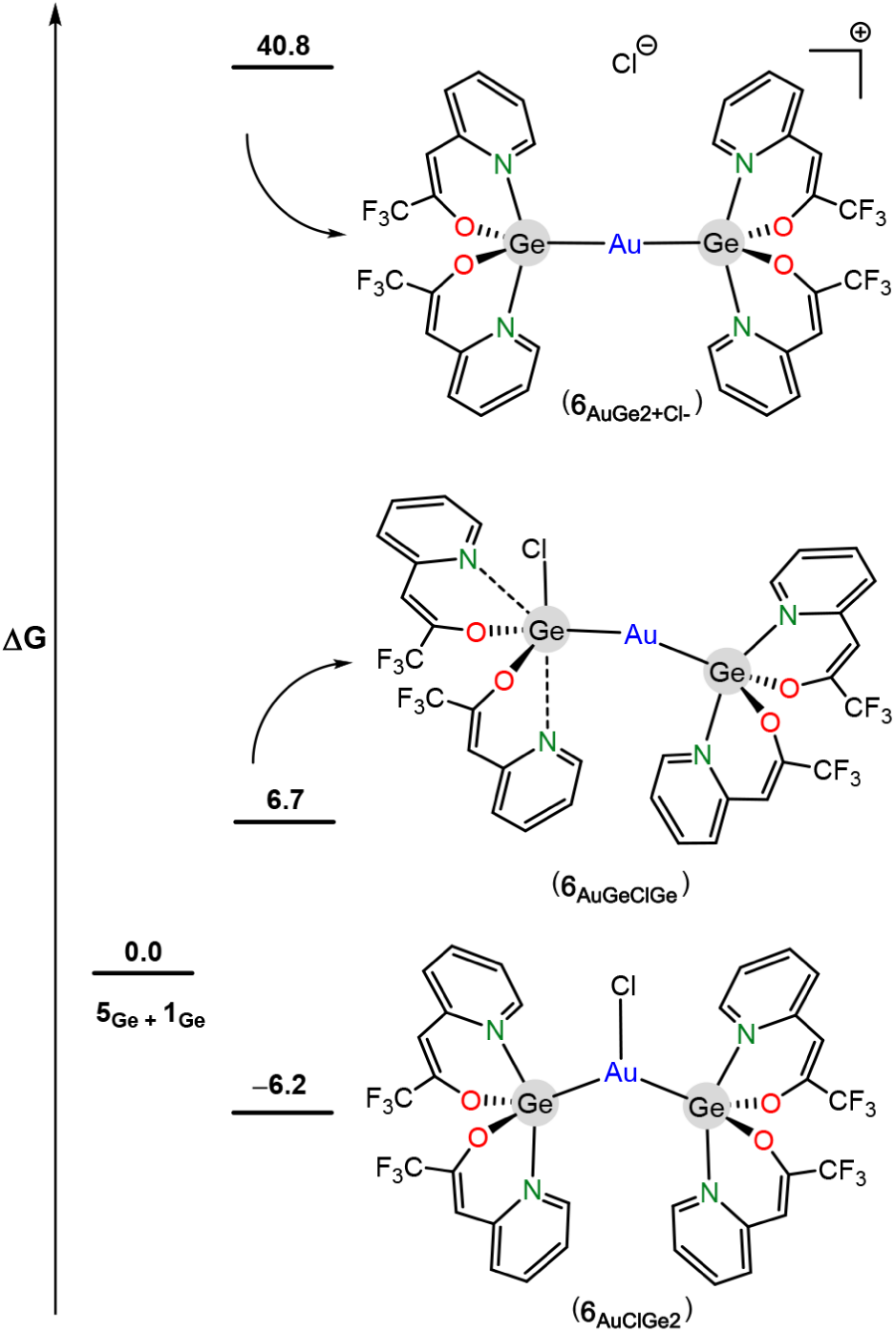
Comparative DFT-calculated (BP86-D3/SDD­(Ge,Au)) energies of **5_Ge_** +**1_Ge_** and the possible isomers of **6_Ge_** (**6_AuClGe2_**, **6_AuGeClGe_** and $6_{\mathrm{AuGe2+C}l^{‐}}$ ). Gibbs energies (CPCM-toluene) are given in kcal mol^–1^.

The air-stability of **5**_**Ge**_ and **6**_**Ge**_ was evaluated acquiring their ^1^H NMR spectra (in C_6_D_6_ or CDCl_3_) under argon and after standing in air for 1 day. For the monogermylene complex **5**_**Ge**_, only the signals of the hydrolytic product tfppOH were observed after the air exposure (Figures S58 and S59). Regarding the disubstituted derivative **6**_**Ge**_ (Figures S60 and S61), in addition to the signals of tfppOH, those of **5**_**Ge**_ could be clearly observed, which also supports the existence of the proposed dissociation equilibrium **6**_**Ge**_ ↔ **5**_**Ge**_ + **1**_**Ge.**_ Note that in both experiments a clear dilution was observed in the NMR spectra after the air exposure, which hints to the existence of other degradation processes (i.e., oxidation, condensation of hydroxo-derivatives, formation of metallic gold, etc.) giving insoluble products (the precipitation of dark solids was, particularly for **5**_**Ge**_, clearly observed).

Regarding the reactivity of **1**_**E**_ with AgOTf, no differences were observed changing the E atom, since the reactions of the silver precursor with two equiv of the ligands (Scheme 5) led to the di-HT complexes [Ag­{κ^1^ *E*-E­(tfppO)_2_}_2_]­OTf (E = Ge (**7**_**Ge**_), Sn (**7**_**Sn**_)), which could be isolated as yellow solids in high yields (>80%). Both products were very insoluble in C_6_D_6_ or CDCl_3_ and were spectroscopically characterized in THF-*d*_8_, showing very similar NMR spectra, with only small variations in the tfppO chemical shifts (see Figures S15 and S17). Their NMR data indicate that both HTs and both tfppOs of each HT are equivalent in solution, since only one set of signals is observed for the four tfppO fragments. Their^19^F­{^1^H} NMR spectra show two singlets in a 4:1 ratio corresponding, respectively, to the CF_3_ groups of the four tfppO fragments (δ −76.30 (**7**_**Ge**_), −76.48 (**7**_**Sn**_) ppm; chemical shift similar to that observed for the other derivatives herein described) and to the CF_3_ moiety of the OTf anion (δ −80.71 (**7**_**Ge**_), −80.51 (**7**_**Sn**_) ppm). The structure of **7**_**E**_ in solution, which could not be established in the solid state by SCXRD, is possibly that depicted in Scheme 5, displaying a cationic linear silver complex not interacting with the OTf counteranion and equipped with two equivalent **1**_**E**_ ligands. This is also proposed considering that the other two crystallographically characterized [Ag­(HT)_2_]­OTf (HT = neutral terminal HT) derivatives,^42^ which also feature five-coordinate E atoms, do not have the OTf groups attached to silver in solution.^43^

**Scheme 5:**
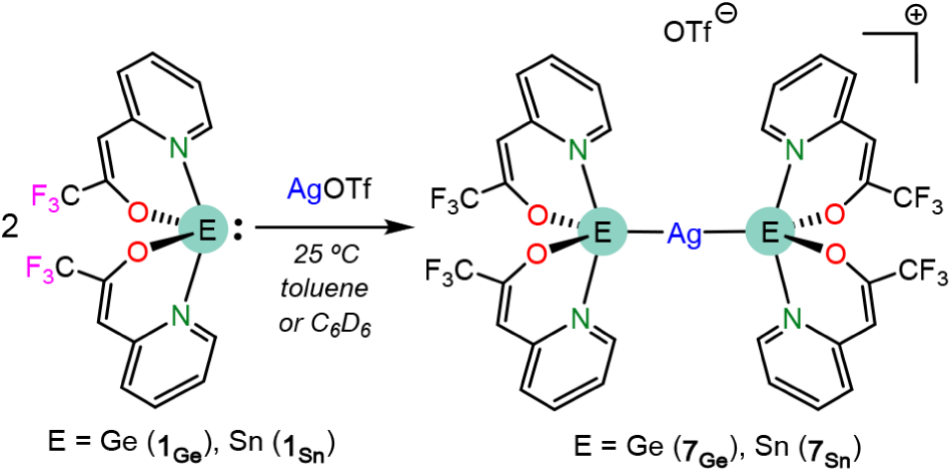
Reactions of 1_E_ with AgOTf

The air-stability of **7**_**Ge**_ and **7**_**Sn**_ was evaluated recording their ^1^H NMR spectra (in THF-*d*_8_) under argon and after standing in air for 1 day. In both cases, the hydrolytic product tfppOH was the major species observed after the air exposure (Figures S62 and 63). However, while for the experiment with **7**_**Ge**_ no traces of the complex were detected, a certain amount of **7**_**Sn**_, maybe in line with the higher air-stability shown by the free stannylene **1**_**Sn**_, survived (tfppOH and tppfO signals of **7**_**Sn**_ appeared in ca. 1:0.4 ratio). Similarly to previous tests, a dilution was observed in the NMR spectra after the air exposure, which was particularly evident for the complex **7**_**Sn**_, where decomposition to metallic silver also occurred.

## 3 Conclusions

This work, aimed at finding appropriate combinations to develop air-stable HT derivatives, has provided new insights, both experimentally and theoretically, into the real potential of the pyridylalkenolato fragment (tfppO), derived from 3,3,3-trifluoro-1-(2-pyridyl)­prop-1-en-2-ol, to render, either free or coordinated, air-stable HTs. This was evaluated on the homoleptic HTs E­(tfppO)_2_ (E = Ge (**1**_**Ge**_), Sn (**1**_**Sn**_)), which were previously reported by Mathur and coworkers,^13^ and on the novel heteroleptic chloro-HTs E­(tfppO)­Cl (E = Ge (**2**_**Ge**_), Sn (**2**_**Sn**_), showing that *(i)* the claimed^13^ high air-stability of **1**_**E**_ has not been confirmed in solution by the air- and water-stability experiments carried out in this work (however, they proved to be much more stable, particularly **1**_**Ge**_, in the solid state), *(ii)* a hydrolytic degradation (formation of tfppOH and/or HCl) is the main (not unique) decomposition pathway occurring (it has been studied by DFT calculations), *(iii)* the homoleptic germylene **1**_**Ge**_ is clearly less stable toward moisture but more stable against atmospheric oxygen than the stannylene **1**_**Sn**_ and *(iv)* the Cl–E bond hydrolysis (vs the tfppO hydrolysis) is only clearly operating for **2**_**Ge**_. DFT calculations have provided new insights into how hydrolytic degradation occurs on HTs, which, at least in the systems of study, starts demanding a simultaneous ambiphilic behavior of both reagents, since water nucleophilically attacks the acidic E atom and electrophilically interacts with the oxygen (of tfppO) or Cl atoms of the HTs. This initial process, which has always a lower activation barrier for tin, leads endergonically (except for **1**_**Ge**_) to hydrolysis and undetected intermediates, which must evolve to thermodynamically very stable products (further hydrolysis, oxidation, condensation, precipitation, etc. is reasonable)^25^ to explain the experimental observations. It should be noted that the participation of multiple equivalents of water, even during the first stages of the hydrolysis process, cannot be discarded.

Additionally, a preliminary study on the coordination chemistry of HTs **1**_**E**_ and **2**_**E**_ has shown that *(i)* the homoleptic derivatives **1**_**E**_ are prone to suffer ligand degradation upon tfppO-transfer (formation of complex **4**), *(ii)* the germylenes are more capable to produce clean reactions and isolable complexes than the stannylenes (**3**_**Ge**_, **5**_**Ge**_, **6**_**Ge**_ and **7**_**Ge**_ for E = Ge and only **7**_**Sn**_ for E = Sn), *(iii)* complex **6**_**Ge**_ is a rare example of a gold complex equipped with two heavy G14-donor ligands and a chloride group, since it does not feature the chlorine atom attached to one of the G14-ligands and *(iv)* different from previous reports in which air-unstable HTs were capable of rendering air-stable complexes,^5,6,27^ complexation of the herein studied HTs does not improve their air/water-stability. This is maybe related, as exemplified by the existence of the room temperature dissociation equilibrium **6**_**Ge**_ ↔ **5**_**Ge**_ + **1**_**Ge**_, with these tfppO-HTs being weakly bound to the metal centers.

## 4 Experimental Section

The entire experimental section is included in the Supporting Information, which is divided into the following sections: **1)** General Procedures, **2)** Synthetic Details and Characterization Data, **3)** Stability Studies of **1**_**E**_ and **2**_**E**_, **4)** Stability Studies **1**_**E**_ and **2**_**E**_ (in the presence of an internal standard), **5)** Stability Studies of the metal complexes, **6)** X-ray Diffraction Data, **7)** Theoretical Calculations and **8)** References. No uncommon hazards are noted derived from the experimental work carried out.

## Supplementary Material





## Notes

The Supporting Information^44^ is available free of charge at https://pubs.acs.org/doi/10.1021/acs.inorgchem.5c05924.

Atomic coordinates of DFT-optimized structures (XYZ)
General procedures, experimental details, characterization data, and NMR spectra (PDF)
