# Supplementary material for: Pyridylalkenolato-Stabilized Heavier Tetrylenes: Air-Stability Studies and Preliminary Coordination Chemistry
Source: Inorg Chem. 2026 May 18;65(21):11592–605. doi: 10.1021/acs.inorgchem.5c05924 (PMC13231423; doi:10.1021/acs.inorgchem.5c05924)
Supplement: Supplementary file 2 [file ic5c05924_si_002.pdf]

SUPPORTING INFORMATION

Pyridylalkenolato-Stabilized Heavier Tetrylenes: Air-Stability Studies and Preliminary Coordination Chemistry

Sergio García-Vega,[†] *Enrique Pérez-Carreño,*[‡] *Javier A. Cabeza,*[†] *and Pablo García-Álvarez**[,†]

†Departamento de Química Orgánica e Inorgánica, Centro de Innovación en Química Avanzada ORFEO-CINQA, Universidad de Oviedo, E-33071 Oviedo, Spain

‡Departamento de Química Física y Analítica, Universidad de Oviedo, E-33071 Oviedo, Spain

Corresponding author E-mail: pga@uniovi.es

Supporting Information Contents

1. **General Procedures** S3

2. **Synthetic Details and Characterization Data** S4

3. **Stability Studies of 1$_E$ and 2$_E$** S26

4. **Stability Studies 1$_E$ and 2$_E$ (in the presence of an internal standard)** S44

5. **Stability Studies of the metal complexes** S70

6. **X-ray Diffraction Data** S79

7. **Theoretical Calculations** S81

8. **References** S87

1. General Procedures

Unless otherwise stated, all reactions and product manipulations were carried out at room temperature under argon in an MBraun UNIlab Pro glove box or using Schlenk-vacuum line techniques. Solvents were dried over appropriate desiccating reagents and distilled under argon before being stored in the drybox in glass containers containing a bed of 4 Å molecular sieves. Compounds Ge(hmds)$_2$ (hmds = N(SiMe$_3$)$_2$),[S1] Sn(hmds)$_2$,[S1] E(tfppO)$_2$ (E = Ge(**1a**), Sn(**1b**)),[S2] 3,3,3-trifluoro-1-(2-pyridyl)prop-1-en-2-ol (tfppOH),[S3] [Ir$_2$Cl$_2$(μ-Cl)$_2$(η^5-Cp*)$_2$] (Cp* = 1,2,3,4,5-pentamethylcyclopentadienyl)[S4] and [AuCl(tht)][S5] were prepared following published procedures. All remaining reagents were purchased from commercial sources. All reagents and solvents were stored under argon in a drybox. All reaction products were vacuum-dried for several hours prior to being weighted and analyzed. NMR spectra were run on Bruker NAV-400, AV-400 and AV-300 instruments using as standard the residual protic solvent resonance for ^{1}H [δ(C*H*Cl$_3$) 7.26 ppm; δ(C$_6$*H*D$_5$) 7.16 ppm; δ(THF-d$_8$) 3.58 ppm] and the solvent resonance for ^{13}C [δ(*C*$_6$D$_6$) 128.1 ppm; δ(*C*DCl$_3$) 77.16 ppm; δ(*H*-THF-d$_8$) 67.21 ppm]. Microanalyses were obtained with a Thermo-Finnigan FlashEA112 microanalyzer. High-resolution mass spectra (HRMS) were obtained with a Bruker Impact II mass spectrometer operating in the ESI-Q-TOF positive mode; data given refer to the most abundant isotopomer of the observed species with the greatest mass. CHN microanalyses were not obtained for **5$_{Ge}$** and **7$_{Sn}$** due to their tendency to undergo degradation processes to, possibly, metallic gold and silver, respectively. No uncommon hazards are noted derived from the experimental work carried out.

2. Synthetic Details and Characterization Data

Ge(tfppO)Cl (2_{Ge}): A 4:1 toluene/Et_2O solvent mixture (10 mL) was added to a glass vial containing Li(hmds) (307 mg, 1.83 mmol) and tfppOH (350 mg, 1.85 mmol) and the resultant brownish solution was stirred for 2 h. Then, $GeCl_2$(dioxane) (423 mg, 1.83 mmol) was added and the resulting brown suspension was stirred for 18 h. An aliquot of the crude reaction mixture was evaporated and analyzed by ^1H NMR in $CDCl_3$, showing the quantitative formation of **2a**. The reaction crude suspension was allowed to settle and the supernatant cloudy solution was filtered through a glass fibre filter. The filtrate was vacuum-evaporated and the resulting residue was washed with hexane (2 x 2 mL) and vacuum-dried to give **2a** as a pale-orange solid (454 mg, 84% yield). Anal. (%) calcd. for $C_8H_5ClF_3GeNO$ (M = 296.19 amu): C, 32.44; H, 1.70; N, 4.73; found: C, 32.39; H, 1.73; N, 4.61. (+)-HRMS: *m/z* 261.9482. Calcd. for $C_8H_5F_3GeNO$: *m/z* 261.9566 $[M - Cl]^+$. ^1H NMR ($CDCl_3$, 300.1 MHz, 298 K, Figure S1): δ 8.39 (dd, J = 5.7, 0.9 Hz, 1 H), 8.02 (td, J = 7.8, 0.9 Hz, 1 H), 7.50 (m, 1 H), 7.42 (dd, J = 7.8, 0.9 Hz, 1 H), 6.33 (s, 1 H). $^{13}C\{^1H\}$ NMR ($CDCl_3$, 75.5 MHz, 298 K, Figure S1): δ 150.9 (q, J = 35 Hz, O*C*CF_3), 149.6 (s, *C*), 143.4 (s, *C*H), 142.1 (s, *C*H), 125.8 (s, *C*H), 123.0 (s, *CH*), 119.6 (q, J = 277 Hz, *C*F_3), 98.9 (s, *C*H). $^{19}F\{^1H\}$ NMR ($CDCl_3$, 282.4 MHz, 298 K, Figure S2): δ –74,02 (s).

Sn(tfppO)Cl (2_{Sn}): A 4:1 toluene/Et_2O solvent mixture (5 mL) was added to a glass vial containing Li(hmds) (168 mg, 1.01 mmol) and tfppOH (191 mg, 1.01 mmol). The resulting brownish solution was stirred for 2 h. Then, $SnCl_2$ (190 mg, 1.00 mmol) was added and the resulting brownish suspension was stirred for 18 h. An aliquot of the reaction crude was evaporated and analyzed by ^1H NMR in $CDCl_3$, showing the quantitative formation of **2b**. The reaction crude was extracted sequentially in 5 mL of a 4:1 toluene/Et_2O mixture (reaction solvents), in 10 mL of a 4:1 toluene/Et_2O mixture and in 30 mL of 10:1 toluene/THF mixture. The extraction procedure consisted in suspending the reaction crude solid in the aforementioned solvent mixture, allowing the suspension to settle and filtering the supernatant cloudy solution through a glass fibre filter. The volatiles of all combined filtrates were removed *in vacuo* and the resulting solid was washed with a 4:1 hexane/dichloromethane mixture (5 mL) and vacuum-dried to give **2b** as a pale-brownish/orange solid. (270 mg, 79% yield). Anal. (%) calcd. for $C_8H_5ClF_3NOSn$ (M = 342.29 amu): C, 28.07; H, 1.47; N, 4.09; found: C, 27.02; H, 1.58; N, 3.81 (this analysis provides the best values obtained to date). (+)-HRMS: *m/z* 343.9107.

Calcd. for $C_8H_6ClF_3NOSn$: *m/z* 343.9138 [*M* + H]$^+$. ^{1}H NMR ($CDCl_3$, 300.1 MHz, 298 K, Figure S3): δ 8.26 (d, *J* = 5.7, Hz, 1 H), 7.92 (t, *J* = 7.8, Hz, 1 H), 7.41–7.32 (m, 2 H), 6.10 (s, 1 H). ^{13}C{^{1}H} NMR ($CDCl_3$, 75.5 MHz, 298 K, Figure S3): δ 152.4 (s, *C*), 144.7 (s, *C*H), 141.3 (s, *C*H), 126.1 (s, *C*H), 122.0 (s, *CH*), 98.2 (s, *C*H). ^{19}F{^{1}H} NMR ($CDCl_3$, 282.4 MHz, 298 K, Figure S4): δ −74,14 (s). The low solubility of **2_{Sn}** in $CDCl_3$ prevented the observation of the quaternary carbons O*C*CF_3 and *C*F_3 even after long acquisition times.

[IrCl(η^5-Cp*){κ^1*Ge*-Ge(tfppO)Cl}] (3_{Ge}): Toluene (3 mL) was added to a glass vial containing **2_{Ge}** (59 mg, 0.2 mmol) and [Ir_2Cl_2(μ-Cl)$_2$(η^5-Cp*)$_2$] (80 mg, 0.1 mmol). The resulting orange suspension was stirred for 16 h (no visual changes after that period). An aliquot of the crude reaction mixture was evaporated and analyzed by ^{1}H NMR in $CDCl_3$, showing the quantitative formation of **3_{Ge}**. The solvent was vacuum-evaporated to give a yellow solid (134 mg, 99 %). Anal. (%) calcd. for $C_{18}H_{20}Cl_3F_3GeIrNO$ (*M* = 694.54 amu): C, 31.13; H, 2.90; N, 2.02; found: C, 31.28; H, 2.91; N, 1.98. (+)-HRMS: *m/z* 659.9646. Calcd. for $C_{18}H_{20}Cl_2F_3GeIrNO$: *m/z* 659.9722 [*M*]$^+$. ^{1}H NMR ($CDCl_3$, 300.1 MHz, 298 K, Figure S6): δ 9.58 (dd, *J* = 6.0, 1.6 Hz, 1 H), 8.06 (td, *J* = 7.7, 1.6 Hz, 1 H), 7.50 (m, 1 H), 7.44 (d, *J* = 8.1 Hz, 1 H), 6.36 (s, 1 H). ^{13}C{^{1}H} NMR ($CDCl_3$, 75.5 MHz, 298 K, Figure S6): δ 150.3 (q, *J* = 36 Hz, O*C*CF_3), 149.3 (s, *C*), 148.1 (s, *C*H), 143.4 (s, *C*H), 125.8 (s, *C*H), 123.7 (s, *CH*), 119.6 (q, *J* = 278 Hz, *C*F_3), 99.9 (s, *C*H), 91.8 (s, *C*, Cp*), 9.0 (s, *C*H_3, Cp*). ^{19}F{^{1}H} NMR ($CDCl_3$, 282.4 MHz, 298 K, Figure S7): δ −74.18 (s).

Reaction of 2_{Sn} with 0.5 equiv. of [Ir_2Cl_2(μ-Cl)$_2$(η^5-Cp*)$_2$]: Toluene (3 mL) was added to a glass vial containing **2_{Sn}** (69 mg, 0.2 mmol) and [Ir_2Cl_2(μ-Cl)$_2$(η^5-Cp*)$_2$] (80 mg, 0.1 mmol). The resulting orange suspension was stirred for 16 h (some sticky brownish solid was formed after that period). An aliquot of the crude reaction mixture was evaporated and analyzed by ^{1}H NMR in $CDCl_3$, showing a complex mixture of unidentified species.

Reaction of 1_{Ge} with 0.5 equiv. of [Ir_2Cl_2(μ-Cl)$_2$(η^5-Cp*)$_2$]: Toluene (3 mL) was added to a glass vial containing **1_{Ge}** (79 mg, 0.18 mmol) and [Ir_2Cl_2(μ-Cl)$_2$(η^5-Cp*)$_2$] (70 mg, 0.09 mmol). The resulting orange suspension was stirred for 16 h. An aliquot of the crude reaction mixture was evaporated and analyzed by ^{1}H NMR in $CDCl_3$, showing a complex mixture of products. The reaction mixture was stirred for 3 additional days. After that period, an aliquot of the reaction crude was analyzed by ^{1}H NMR in $CDCl_3$, showing a slow evolution to a major species, which could be accelerated heating up the $CDCl_3$

solution at 60 ºC (after solvent removal and transfer of the resultant brown/orange solid residue to a J.-Young sealed NMR tube). The major species (see Figure S8) was later identified as complex **4**.

Reaction of 1_{Sn} with 0.5 equiv. of [Ir_2Cl_2(μ-Cl)$_2$(η^5-Cp*)$_2$]: $CDCl_3$ (0.5 mL) was added to a glass vial containing **1_{Sn}** (50 mg, 0.1 mmol) and [Ir_2Cl_2(μ-Cl)$_2$(η^5-Cp*)$_2$] (40 mg, 0.05 mmol). The resulting orange suspension was then stirred for 16 h. The crude reaction mixture was analyzed by ^{1}H NMR in $CDCl_3$, showing a mixture of different species which contained some complex **4** (Figure S9).

[IrCl(η^5-Cp*){κ^2*N,O*-(tfppO)}] (4): A 4:1 toluene/Et_2O mixture (0.5 mL) was added to a glass vial containing Li(hmds) (8.4 mg, 0.05 mmol) and 2-PyCHC(CF_3)OH (9.5 mg, 0.05 mmol). The resulting brownish solution was stirred for 2 h. Then, [Ir_2Cl_2(μ-Cl)$_2$(η^5-Cp*)$_2$] (20 mg, 0.025 mmol) was added and the resulting orange suspension was stirred for 2 h. The initial orange color changed to yellow. Solvents were vacuum-evaporated and the resulting yellow solid residue was dissolved in $CDCl_3$ and analyzed by ^{1}H NMR, showing the quantitative formation of compound **4**. Then, $CDCl_3$ was removed *in vacuo* and the resulting solid was extracted in 1 mL of a 4:1 toluene/Et_2O mixture. The extraction procedure consisted in suspending the reaction crude solid in the aforementioned solvent mixture, allowing the suspension to settle and filtering the supernatant cloudy solution through a glass fibre filter. The solvents of the filtrate were removed *in vacuo* to give **4** as a yellow solid (21 mg, 85 %). ^{1}H NMR ($CDCl_3$, 300.1 MHz, 298 K, Figure S10): δ 8.43 (d, J = 5.9 Hz, 1 H), 7.54 (t, J = 7.7 Hz, 1 H), 6.93–6.81 (m, 2 H), 5.45 (s, 1 H), 1.50 (s, 15 H). Anal. (%) calcd. for $C_{18}H_{20}ClF_3IrNO$ (M = 551.02 amu): C, 39.24; H, 3.66; N, 2.54; found: C, 37.24; H, 3.69; N, 2.58 (this analysis provides the best values obtained to date). (+)-HRMS: *m/z* 552.0807. Calcd. for $C_{18}H_{21}ClF_3IrNO$: *m/z* 552.0870 [M + H]$^+$.

[AuCl{κ^1*Ge*-Ge(tfppO)$_2$}] (5_{Ge}): C_6D_6 (0.5 mL) was added to a glass vial containing **1_{Ge}** (22.5 mg, 0.05 mmol) and [AuCl(tht)] (16 mg, 0.05 mmol). The resulting yellowish suspension was stirred for 10 min (a small amount of dark-purple solid formed after that period). The crude reaction mixture was analyzed by ^{1}H NMR, showing the formation of **5_{Ge}** as unique product in solution. Solvents were vacuum-evaporated and the resultant brownish solid residue was suspended in toluene (0.5 mL). The resultant suspension was filtered through a glass fibre filter and the solvents of the filtrate were vacuum-evaporated

to give **5_{Ge}** as a pale-brown solid (31 mg, 91 %). (+)-HRMS: *m/z* 682.9321. Calcd. for $C_{16}H_{11}AuClF_6GeN_2O_2$: *m/z* 682.9321 [*M* + H]$^+$. ^{1}H NMR (C_6D_6, 400.5 MHz, 298K, Figure S11): δ 8.73 (d, *J* = 5.6 Hz, 1 H), 6.61 (t, *J* = 7.4 Hz, 1 H), 6.20 (t, *J* = 6.6 Hz, 1 H), 6.02 (d, *J* = 7.9 Hz, 1H), 5.73 (s, 1 H). ^{13}C{^{1}H} NMR ($CDCl_3$, 101.7 MHz, 298 K, Figure S11): δ 149.7 (s, *C*), 148.5 (q, *J* = 37 Hz, O*C*CF$_3$), 144.9 (s, *C*H), 140.0 (s, *C*H), 124.4 (s, *C*H), 123.1 (s, *CH*), 120.1 (q, *J* = 276 Hz, *C*F$_3$), 100.5 (s, *C*H), 31.66 (s, *C*H$_2$ THT), 30.91 (s, CH$_2$ THT). The ^{13}C{^{1}H} NMR data of **5_{Ge}** were extracted from the spectrum of the crude outcome of the reaction of **1_{Ge}** with [AuCl(tht)] due to the tendency of isolated **5_{Ge}** to undergo decomposition processes (precipitation of dark solids) during the required long acquisition time. ^{19}F{^{1}H} NMR ($CDCl_3$, 282.4 MHz, 298 K, Figure S12): δ −73.63 (s).

[AuCl{κ^1*Ge*-Ge(tfppO)$_2$}$_2$] (6_{Ge}): C_6D_6 (0.5 mL) was added to a glass vial containing **1_{Ge}** (22.5 mg, 0.05 mmol) and [AuCl(THT)] (8 mg, 0.025 mmol). The resulting yellow suspension was then stirred for 10 min. The crude reaction mixture was analyzed by ^{1}H NMR, showing the quantitative formation of **6_{Ge}**. The solvent was vacuum-evaporated to give **6_{Ge}** as a yellowish solid (27 mg, 96%). Anal. (%) calcd. for $C_{32}H_{20}AuClF_{12}Ge_2N_4O_4$ (*M* = 1130.15 amu): C, 34.01; H, 1.78; N, 4.96; found: C, 31.48; H, 2.07; N, 4.40 (this analysis provides the best values obtained to date). (+)-HRMS: The molecular ion (*M*$^+$) and/or its fragments could not be identified in the obtained spectra. ^{1}H NMR (C_6D_6, 300.1 MHz, 298 K, Figure S13): δ 8.52 (br s, 1 H), 6.70 (m, 1 H), 6.28 (m, 1 H), 6.13 (d, *J* = 7.6 Hz, 1 H), 5.82 (s, 1 H). ^{13}C NMR (100.7 MHz, $CDCl_3$, 298K, Figure S13): δ δ 150.8 (s, *C*), 149.4 (q, *J* = 34 Hz, O*C*CF$_3$), 145.1 (s, *C*H), 140.0 (s, *C*H), 124.4 (s, *C*H), 123.1 (s, *CH*), 119.5 (q, *J* = 287 Hz, *C*F$_3$), 100.0 (s, *C*H). ^{19}F{^{1}H} NMR ($CDCl_3$, 282.4 MHz, 298 K, Figure S14): δ −73.84 (s). Only the most intense signals of the quaternary carbons *C*CF$_3$ and *C*F$_3$ were observed even after prolonged acquisition times, possibly due to the low solubility of **6_{Ge}** in $CDCl_3$.

Reaction of 1_{Sn} with one equiv. of [AuCl(tht)]: C_6D_6 (0.5 mL) was added to a glass vial containing **1_{Sn}** (25 mg, 0.05 mmol) and [AuCl(tht)] (16 mg, 0.05 mmol). The resulting dark suspension (a considerable amount of dark-purple solid was instantly formed) was stirred for 10 min. The crude reaction mixture was analyzed by ^{1}H NMR, showing a highly diluted unidentified mixture.

Reaction of 1_{Sn} with 0.5 equiv. of [AuCl(tht)]: C_6D_6 (0.5 mL) was added to a glass vial containing **1_{Sn}** (25 mg, 0.05 mmol) and [AuCl(tht)] (8 mg, 0.025 mmol). The resulting dark suspension was stirred for 10 min. The crude reaction mixture was analyzed by ^{1}H NMR, showing a highly diluted unidentified mixture.

[Ag{κ^1*Ge*-Ge(tfppO)$_2$}$_2$]OTf (7_{Ge}): Toluene (0.5 mL) was added to a glass vial containing **1a** (22.5 mg, 0.05 mmol) and AgOTf (6.4 mg, 0.025 mmol), giving a yellow suspension that was stirred protected from light for 30 min. The solvent was vacuum-evaporated to give **6_{Ge}** as a yellow solid (24 mg, 83 %). Anal. (%) calcd. for $C_{33}H_{20}AgF_{15}Ge_2N_4O_7S$ (M = 1154.66 amu): C, 34.33; H, 1.75; N, 4.85; found: C, 32.81; H, 1.95; N, 4.47 (this analysis provides the best values obtained to date). (+)-HRMS: *m/z* 1154.8473. Calcd. for $C_{33}H_{21}AgF_{15}Ge_2N_4O_7S$: *m/z* 1154.8426 $[M + H]^+$. ^{1}H NMR (THF-d_8, 400.5 MHz, 298 K, Figure S15): δ 8.77 (d, J = 5.5 Hz, 1 H), 7.88 (t, J = 7.9 Hz, 1 H), 7.53–7.16 (m, 2 H), 6.13 (s, 1 H). ^{13}C NMR (THF-d_8, 100.7 MHz, 298 K, Figure S15): δ 151.4 (s, *C*), 149.5 (q, J = 33.7 Hz, O*C*CF$_3$), 145.8 (s, *C*H), 140.9 (s, *C*H), 124.9 (s, *C*H), 123.6 (s, *C*H), 121.1 (q, J = 277 Hz, *C*F$_3$), 100.4 (s, CH). ^{19}F{^{1}H} NMR (THF-d_8, 282.4 MHz, 298 K, Figure S16): δ −76.30 (s, C*F*$_3$), −80.71 (s, OTf).

[Ag{κ^1*Sn*-Sn(tfppO)$_2$}$_2$]OTf (7_{Sn}): C_6D_6 (0.5 mL) was added to a glass vial containing **1_{Sn}** (16 mg, 0.032 mmol) and AgOTf (4.1 mg, 0.016 mmol), giving an orange suspension that was stirred protected from light for 30 min. The crude reaction mixture was analyzed by ^{1}H NMR, showing a very diluted NMR spectrum as a consequence of the very low solubility of the reaction product in C_6D_6. The solvent was vacuum-evaporated to give **7_{Sn}** as a yellow solid (16 mg, 80 %). (+)-HRMS: The molecular ion (M^+) and/or its fragments could not be identified in the obtained spectra. ^{1}H NMR (THF-d_8, 401.5 MHz, 298 K, Figure S17): δ 8.66 (d, J = 5.6 Hz, 1 H), 7.90 (t, J = 7.8 Hz, 1 H), 7.46–7.28 (m, 2 H), 5.96 (s, 1 H). ^{13}C NMR (THF-d_8, 100.7 MHz, 298K, Figure S17): δ 153.5 (s, C), 152.1 (q, J = 34 Hz, O*C*CF$_3$), 145.6 (s, *C*H), 140.0 (s, *C*H), 124.4 (s, *C*H), 121.6 (s, *C*H), 119.2 (q, J = 289 Hz, *C*F$_3$), 97.5 (s, 1CH). ^{19}F{^{1}H} NMR (THF, 282.4 MHz, 298 K, Figure S18): δ −76.02 (s, C*F*$_3$), −76.72 (s, OTf).

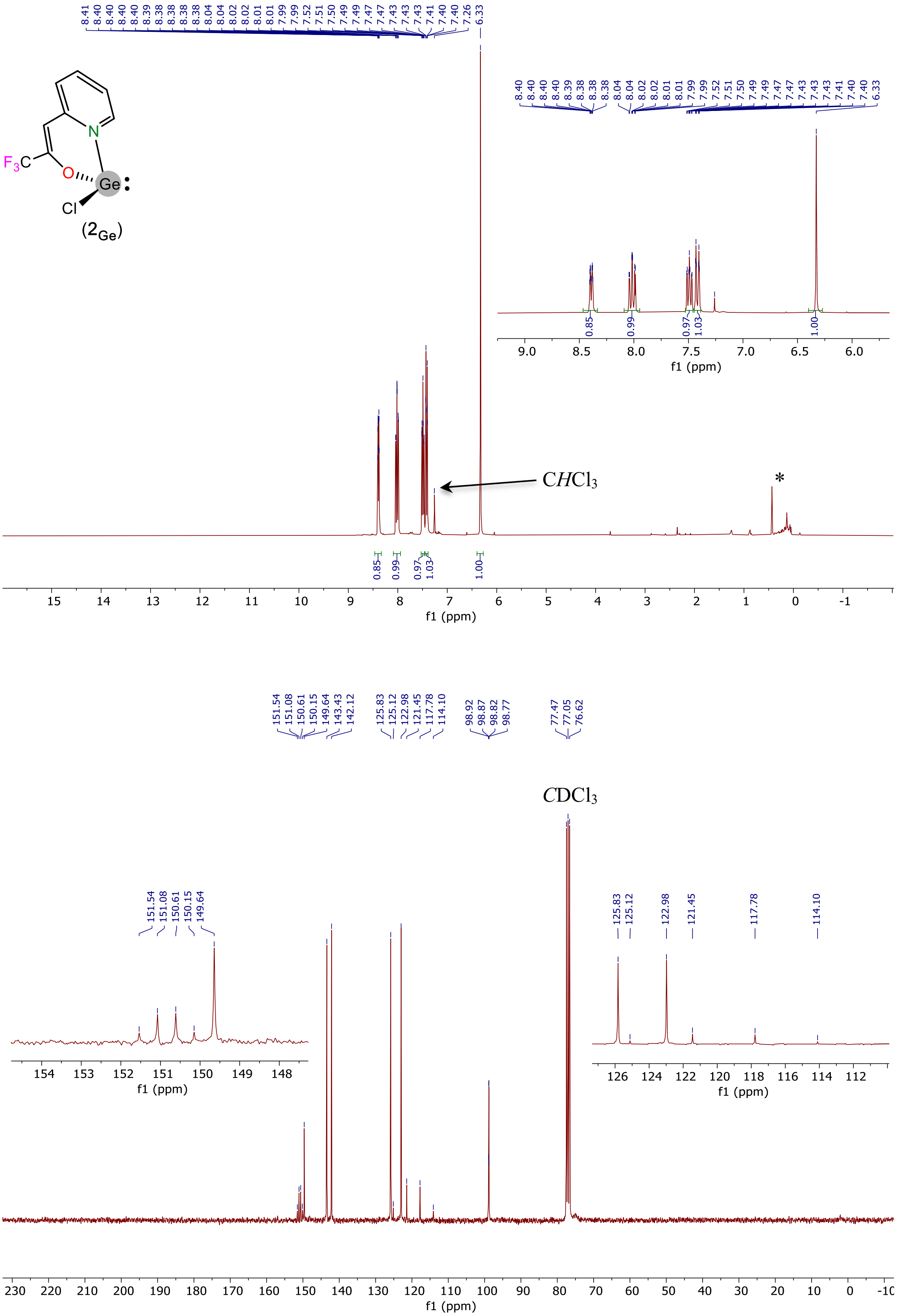

Figure S1. ^{1}H (top, 300.1 MHz) and $^{13}C\{^{1}H\}$(bottom, 75.5 MHz) NMR spectra ($CDCl_3$, 298 K) of $\mathbf{2_{Ge}}$ (* = impurities).

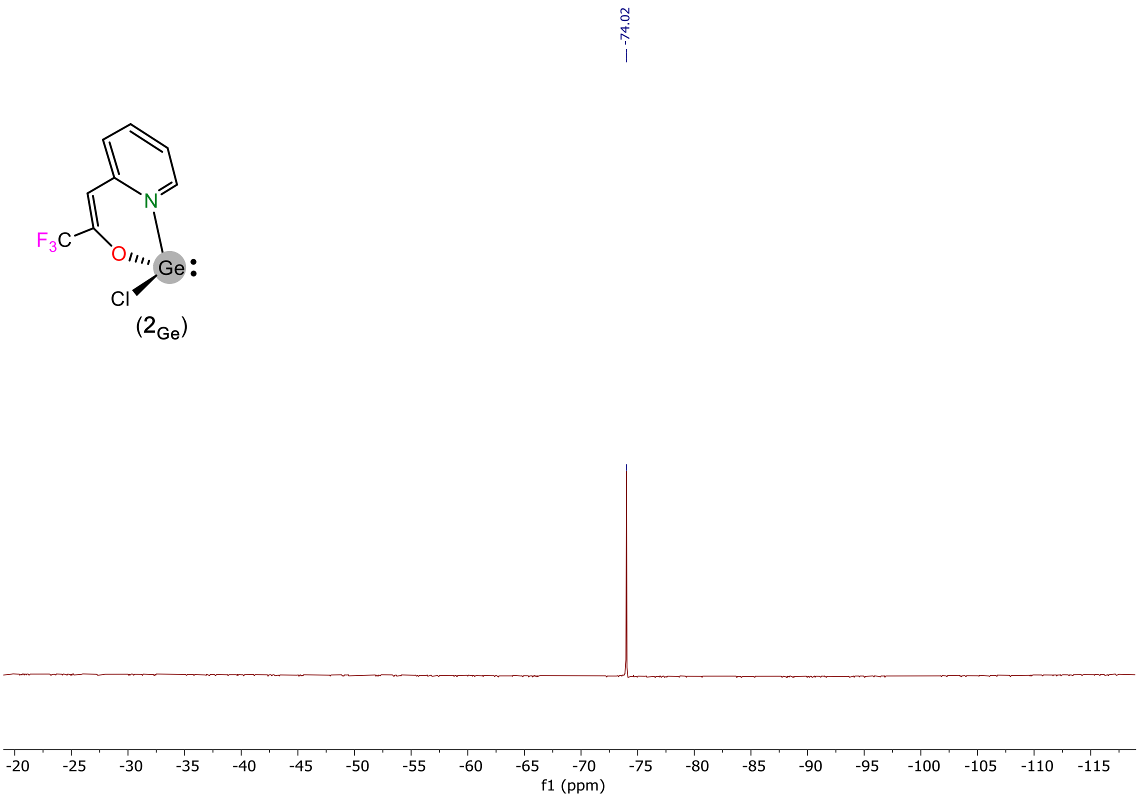

Figure S2. $^{19}F\{^{1}H\}$ (282.4 MHz) NMR spectrum ($CDCl_3$, 298 K) of $\mathbf{2_{Ge}}$.

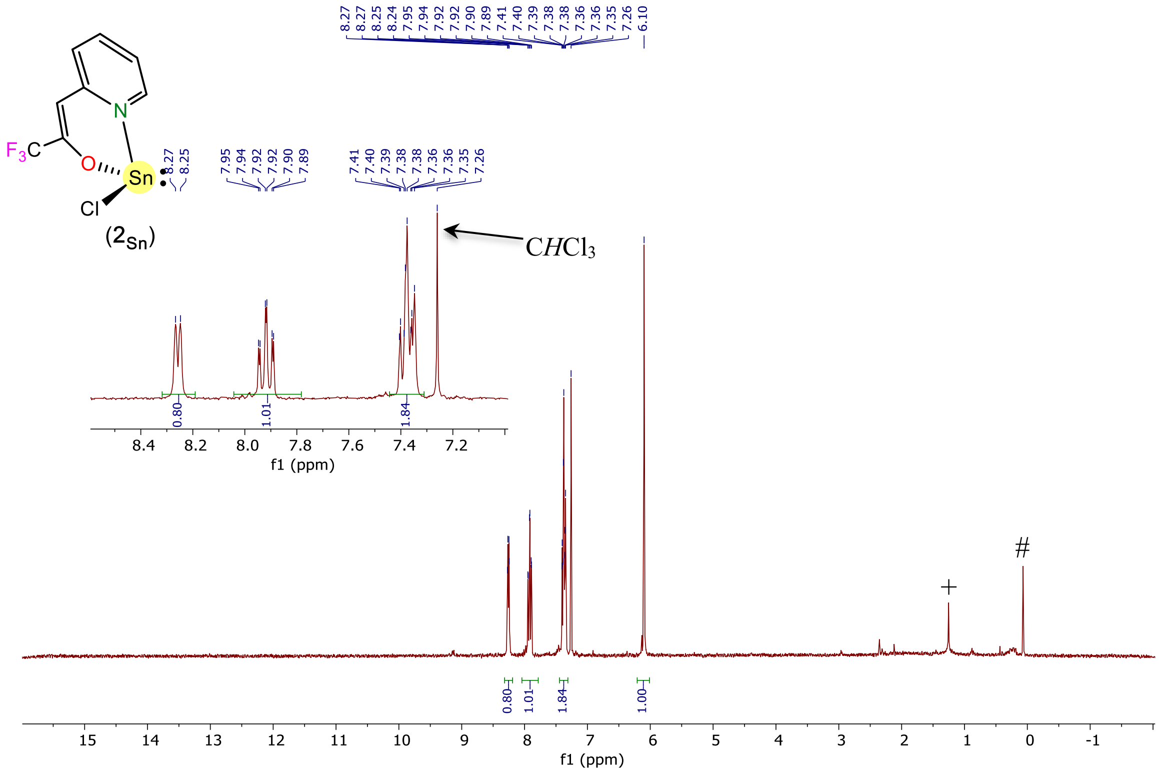

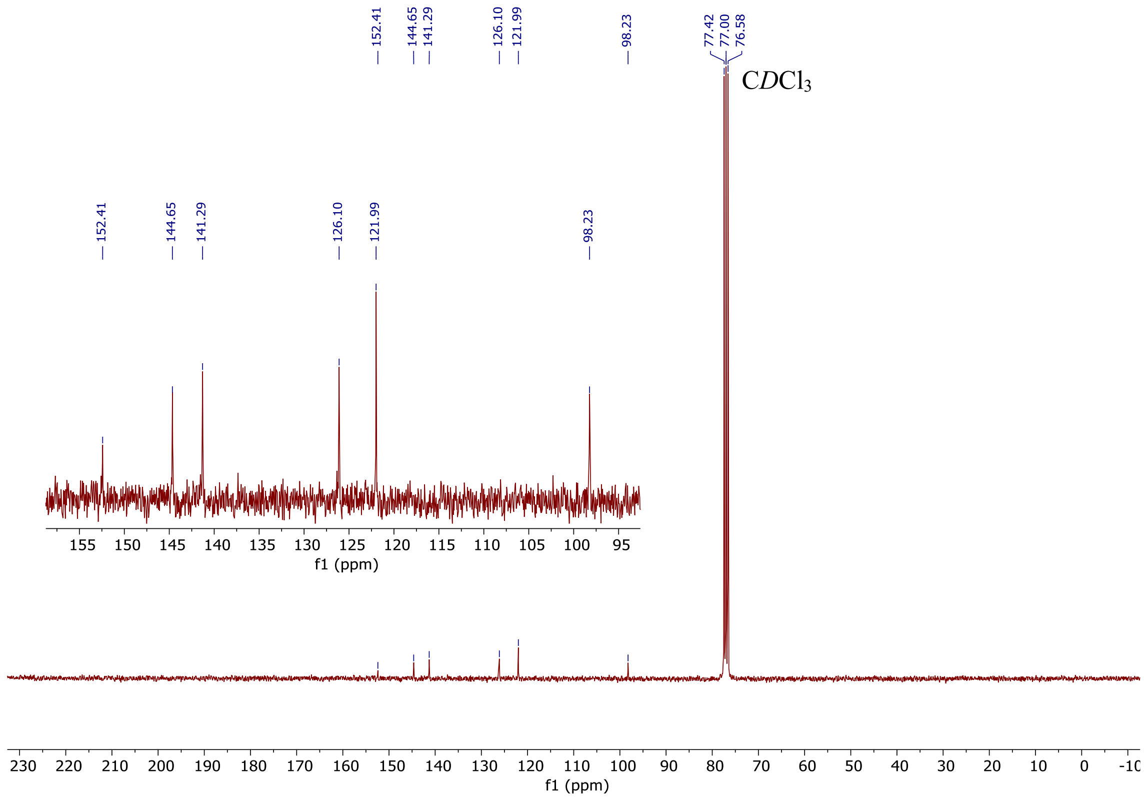

Figure S3. ^{1}H (top, 300.1 MHz) and $^{13}C\{^{1}H\}$ (bottom, 75.5 MHz) NMR spectra ($CDCl_3$, 298 K) of $\mathbf{2}_{Sn}$. (+ = paraffin grease, # = silicone grease).

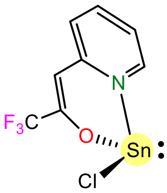

(2_{Sn})

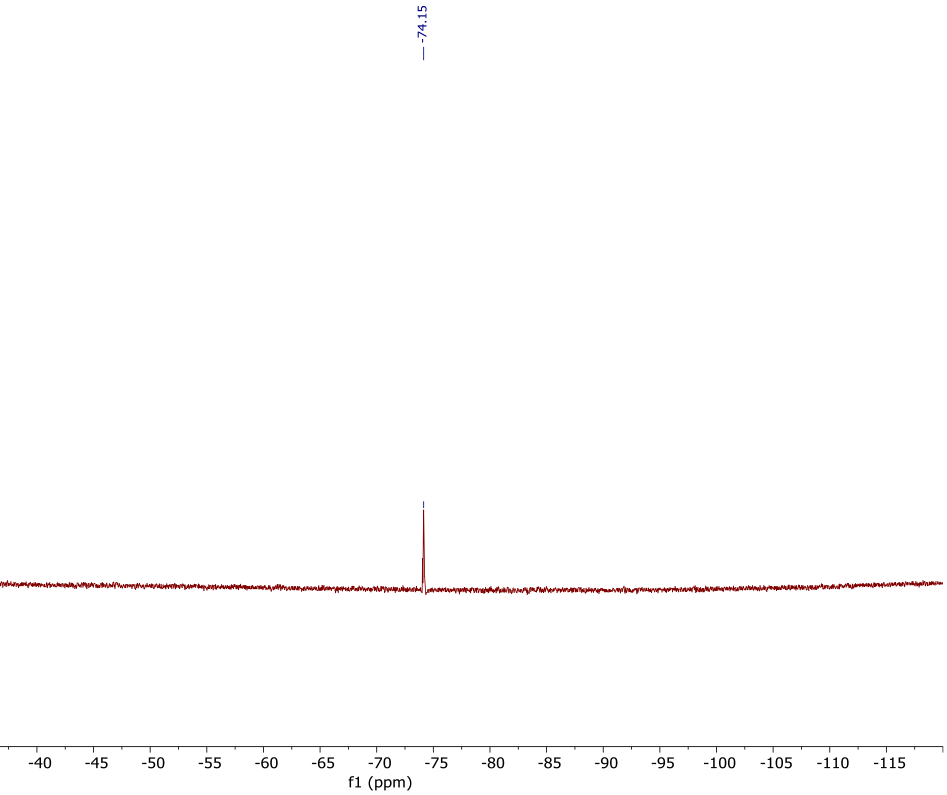

Figure S4. $^{19}F\{^1H\}$ (282.4 MHz) NMR spectrum ($CDCl_3$, 298 K) of $\mathbf{2_{Sn}}$.

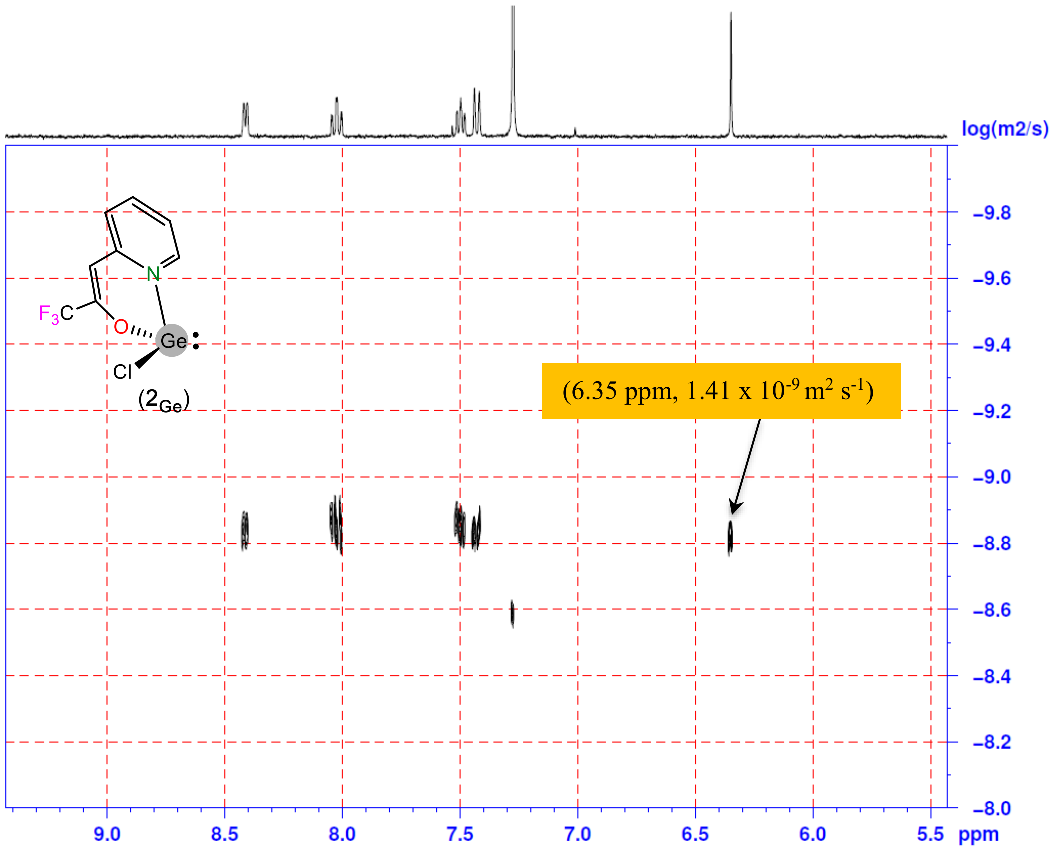

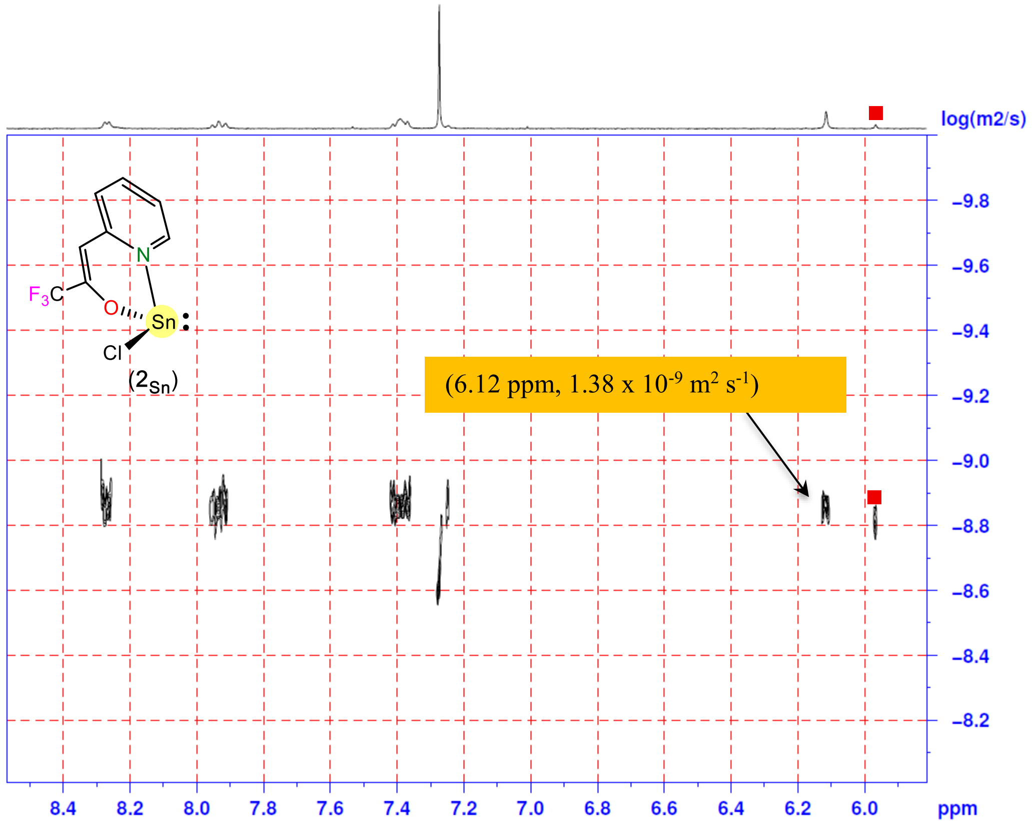

Figure S5. ^{1}H-DOSY NMR spectra ($CDCl_3$, 298 K) of 5.85 mM solutions of $\mathbf{2}_{\mathbf{Ge}}$ (top) and $\mathbf{2}_{\mathbf{Sn}}$ (bottom).

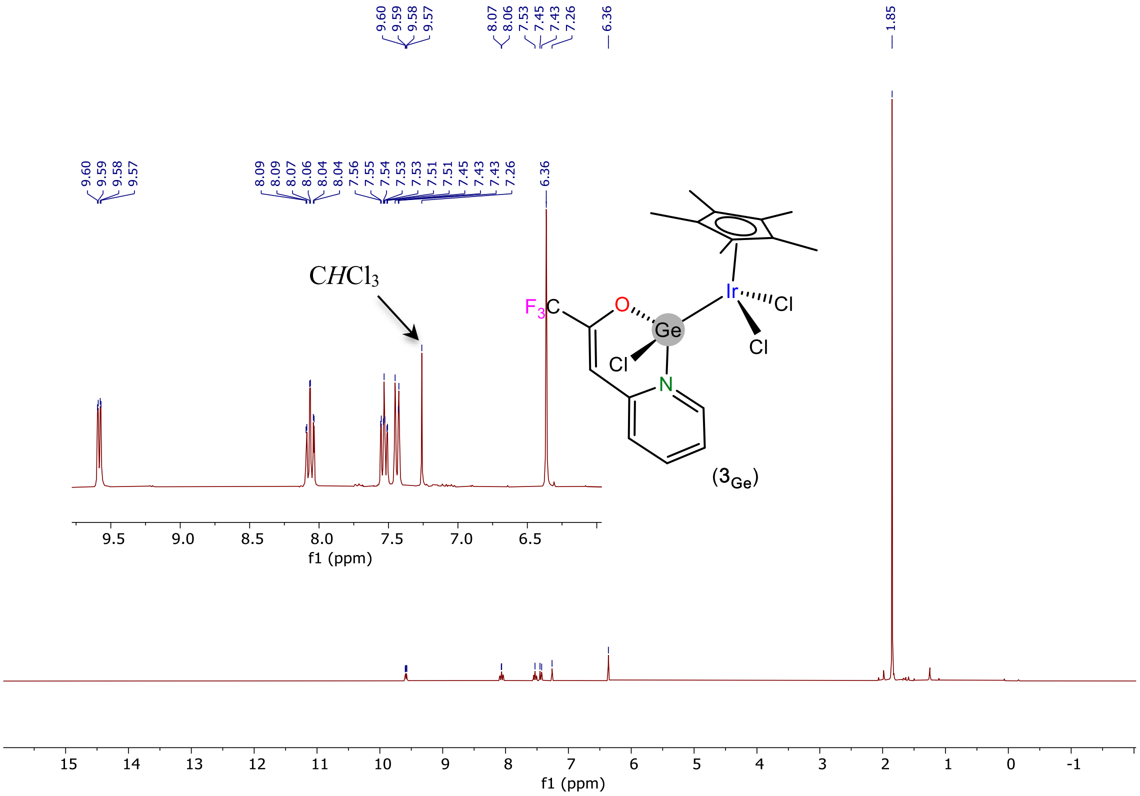

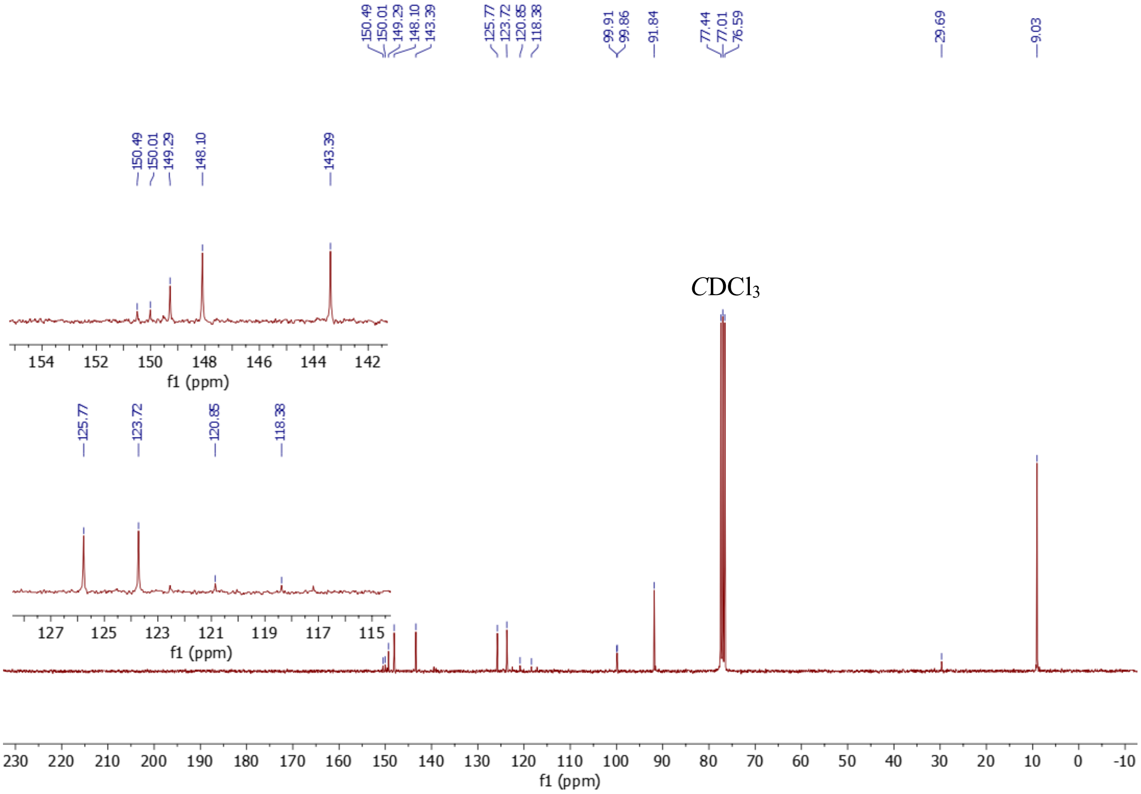

Figure S6. ^{1}H (top, 300.1 MHz) and $^{13}C\{^1H\}$(bottom, 75.5 MHz) NMR spectra ($CDCl_3$, 298 K) of **3**$_{\mathbf{Ge}}$.

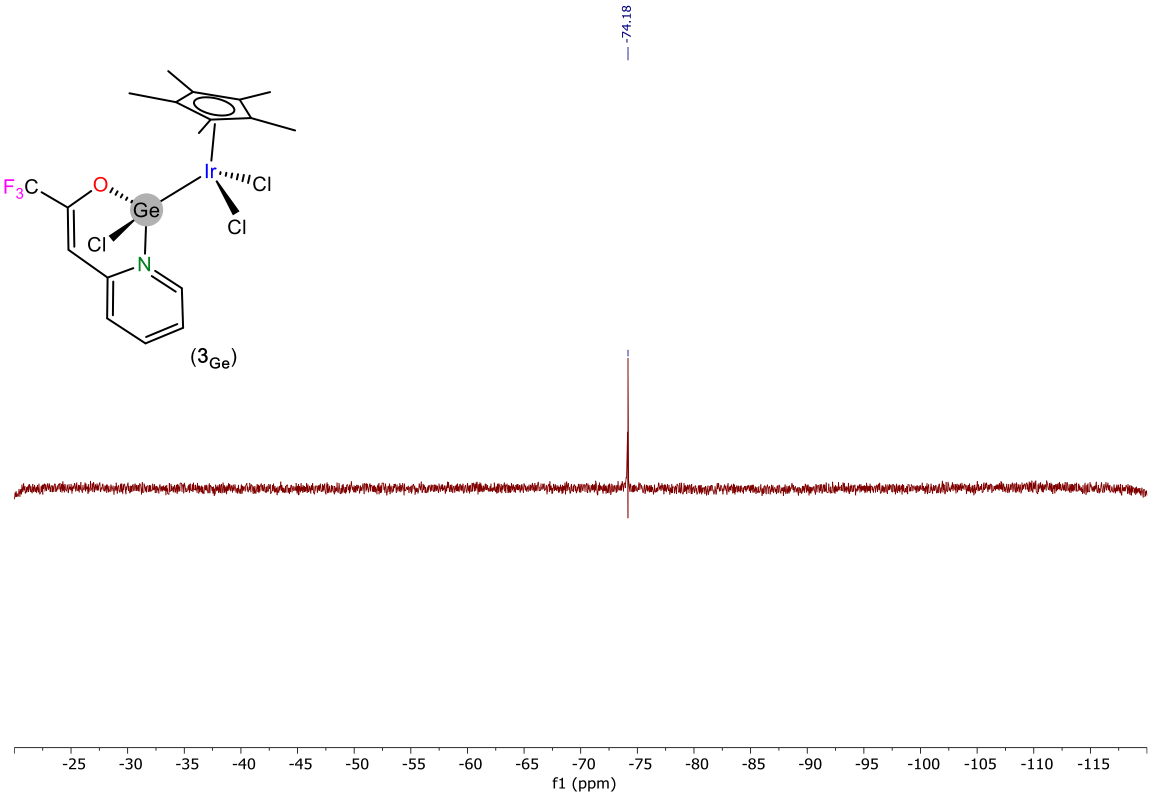

Figure S7. ^{19}F{^{1}H} (282.4 MHz) NMR spectrum ($CDCl_3$, 298 K) of **3_{Ge}**.

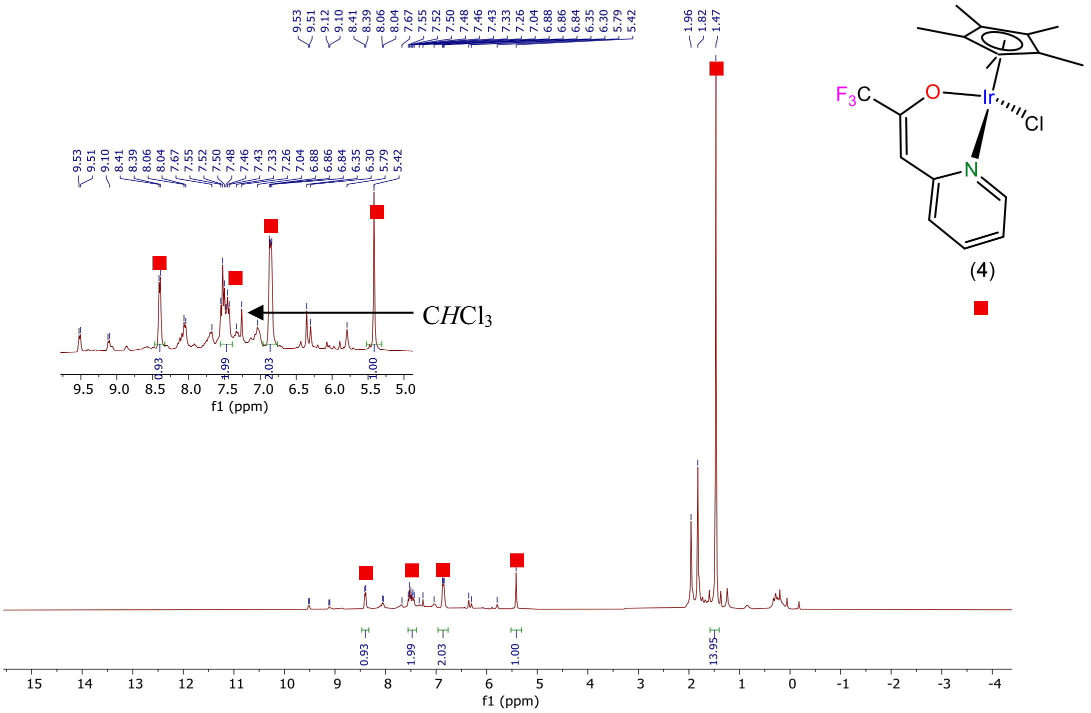

Figure S8. ^{1}H (300.1 MHz) NMR spectra ($CDCl_3$, 298 K) of the crude outcome of the reaction of $\mathbf{1}_{Ge}$ with 0.5 equiv. of [$Ir_2Cl_2(\mu\text{-}Cl)_2(\eta^5\text{-}Cp^*)_2$] after stirring at r.t. for ca. 4 days and 30 h at 60 ºC.

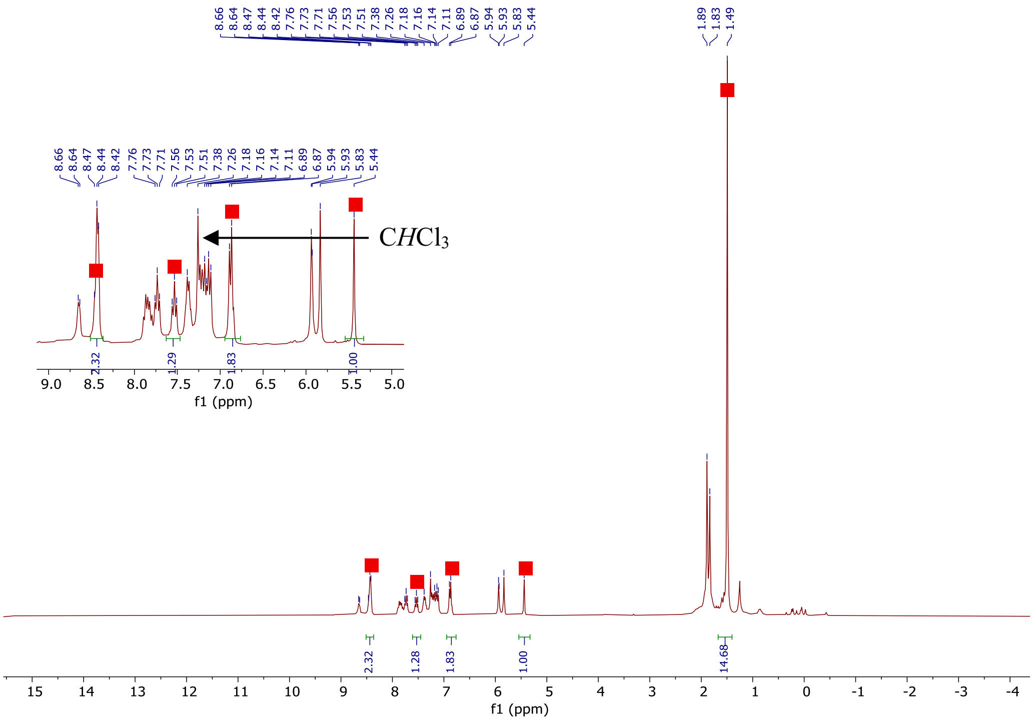

Figure S9. ^{1}H (300.1 MHz) NMR spectra ($CDCl_3$, 298 K) of the crude outcome of the reaction of $\mathbf{1}_{Sn}$ with 0.5 equiv. of [$Ir_2Cl_2(\mu\text{-}Cl)_2(\eta^5\text{-}Cp^*)_2$] after stirring at r.t. for 16 h.

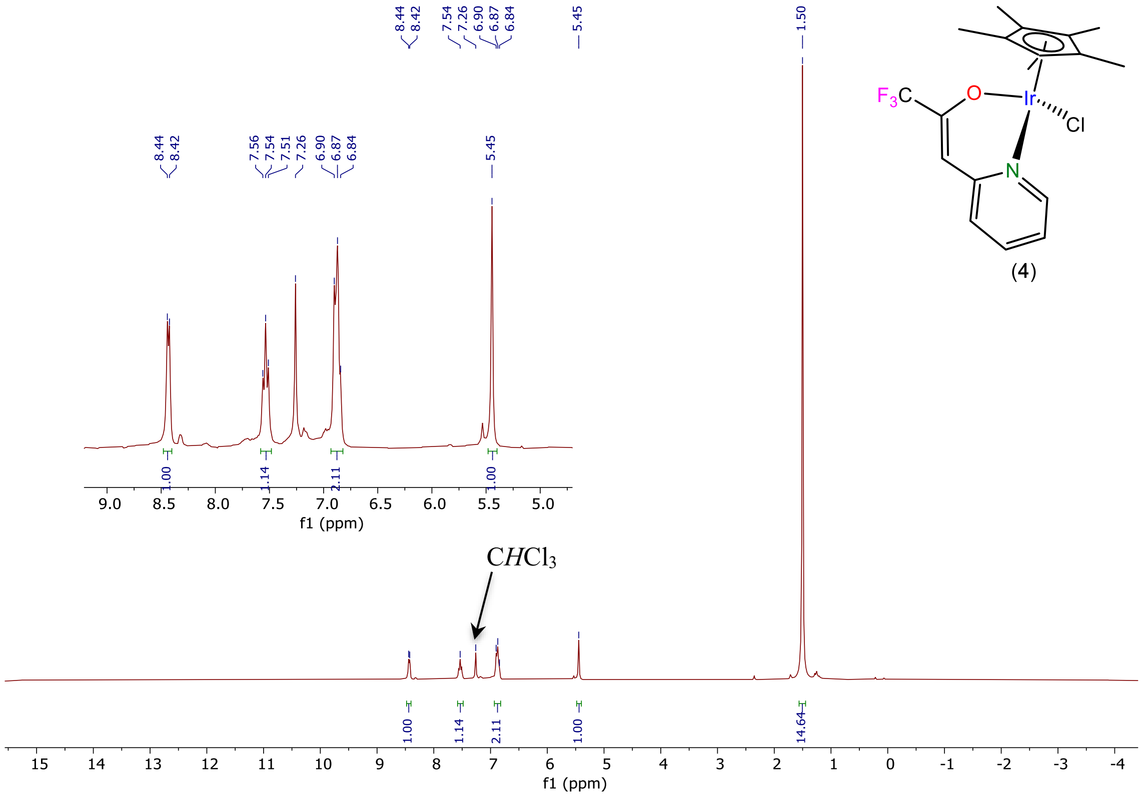

Figure S10. ^{1}H (300.1 MHz) NMR spectrum ($CDCl_3$, 298 K) of complex **4**.

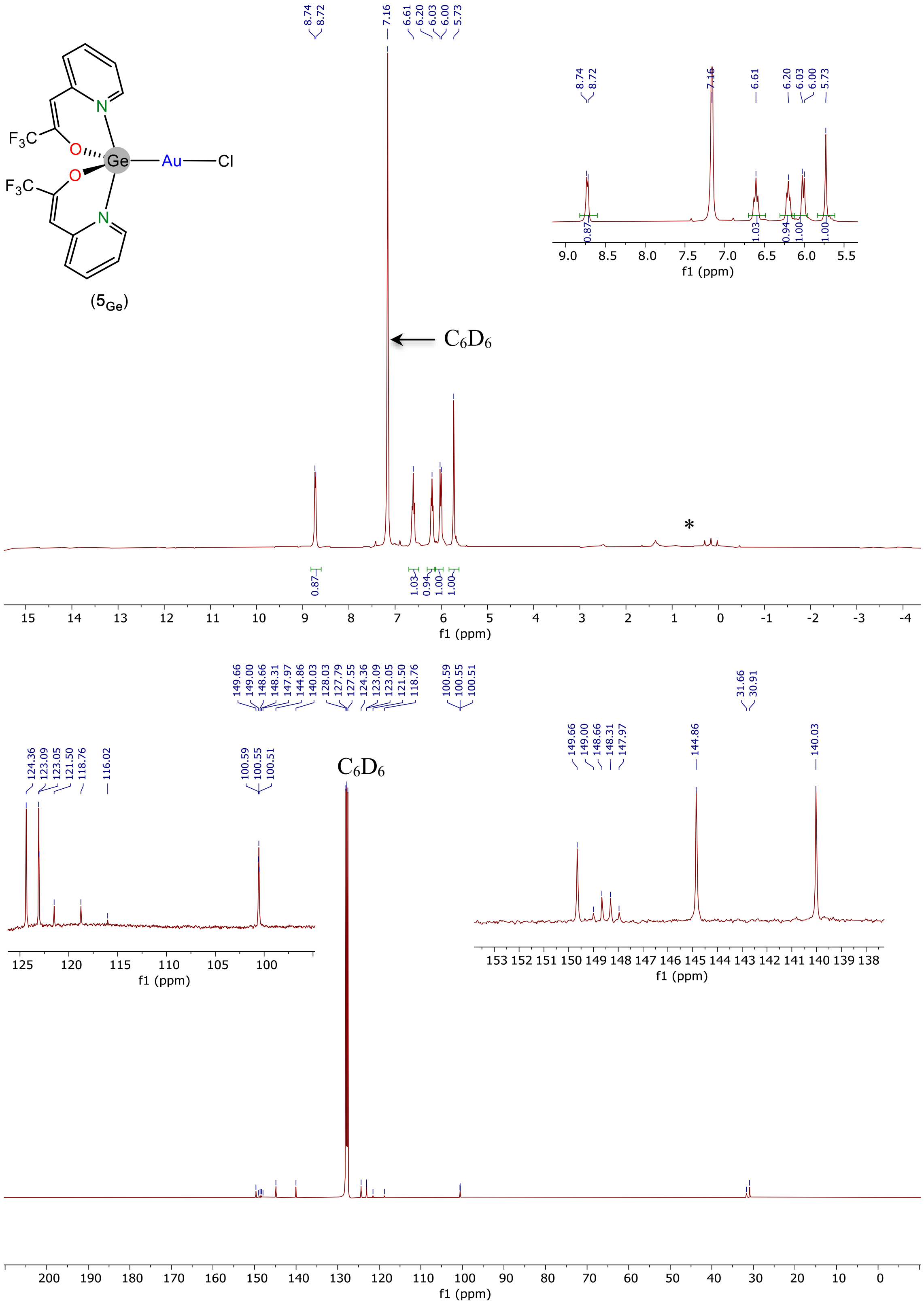

Figure S11. ^{1}H (top, 400.5 MHz) NMR spectrum (C_6D_6, 298 K) of **5**$_{\mathbf{Ge}}$ and ^{13}C{^{1}H}(bottom, 100.7 MHz) NMR spectrum (C_6D_6, 298 K) of the crude outcome of the reaction of **1**$_{\mathbf{Ge}}$ with [AuCl(tht)] after stirring at r.t. for ca. 10 min (* = impurities).

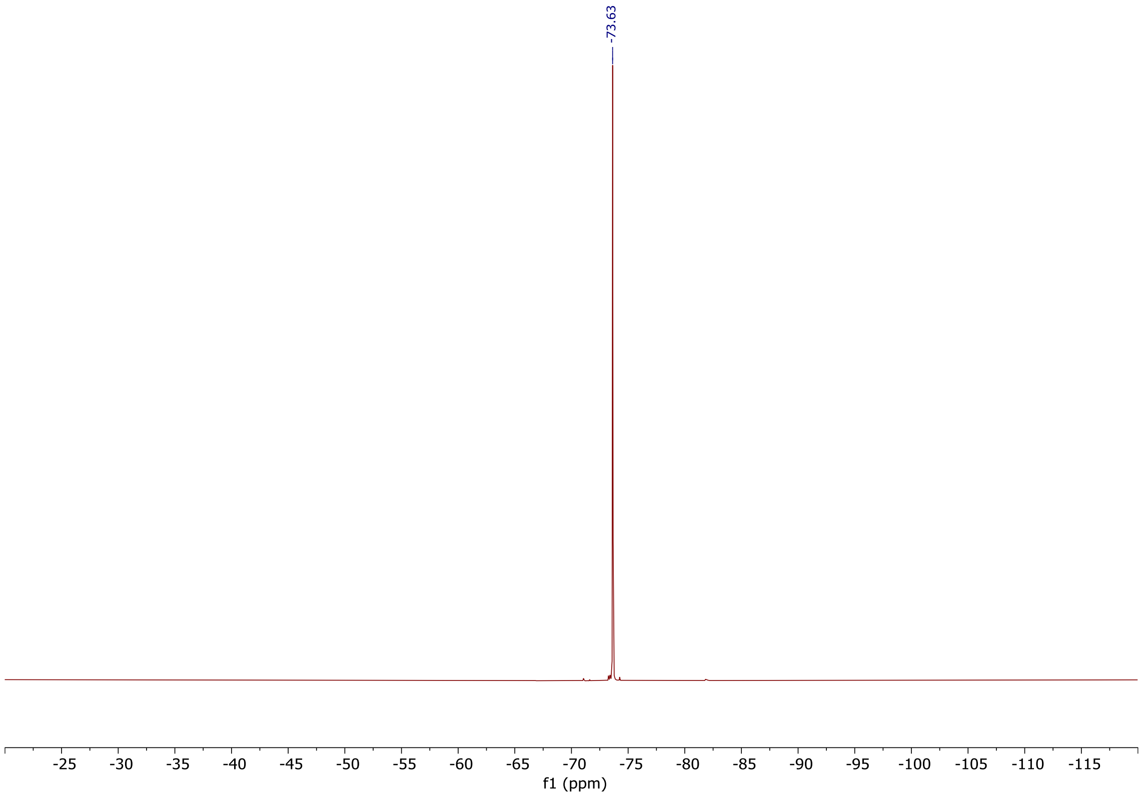

Figure S12. $^{19}F\{^{1}H\}$ (282.4 MHz) NMR spectrum (C_6D_6, 298 K) of $\mathbf{5_{Ge}}$.

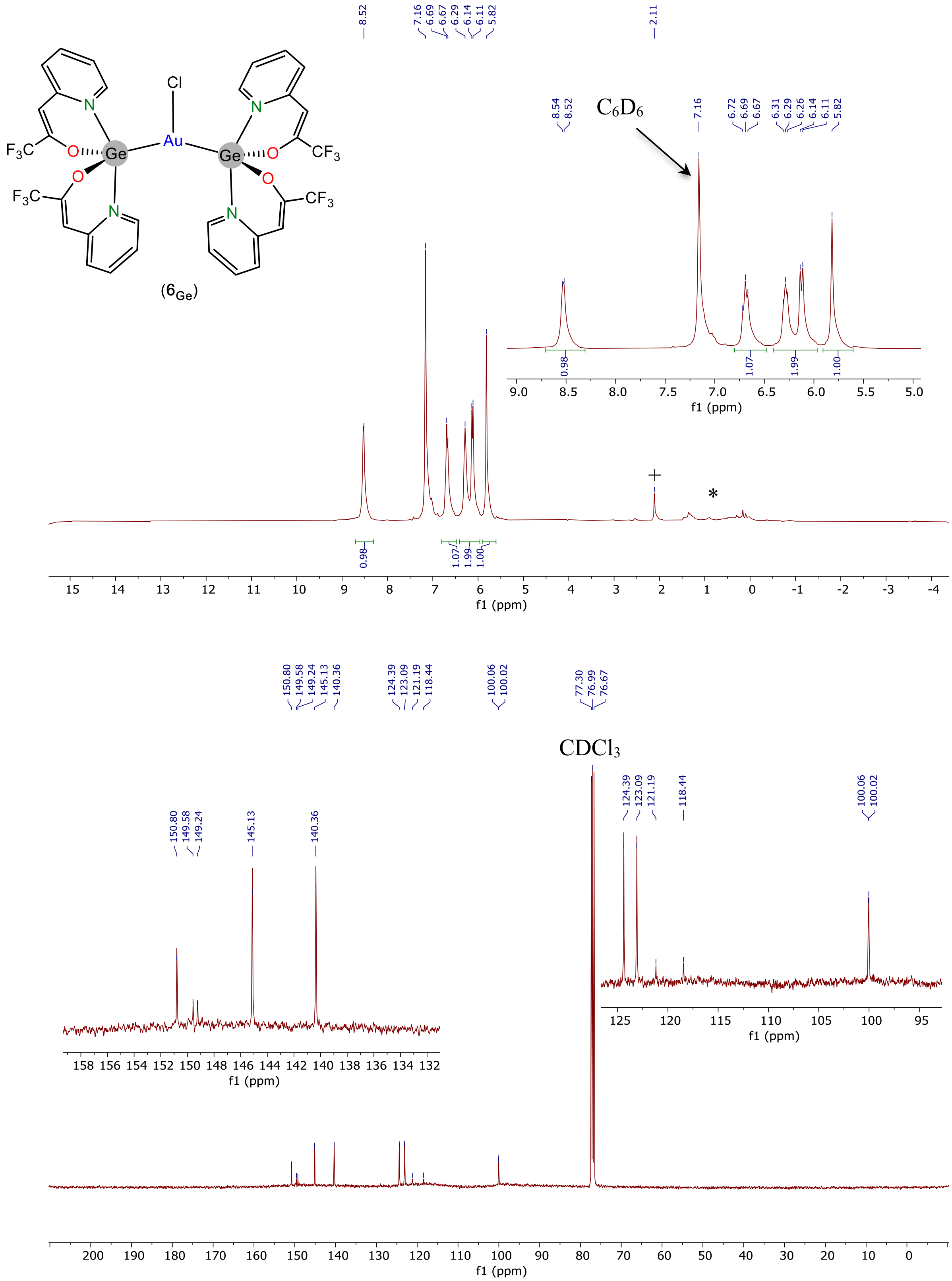

Figure S13. ^{1}H (top, 400.5 MHz, C_6D_6) and $^{13}C\{^{1}H\}$(bottom, 100.7 MHz) NMR spectra ($CDCl_3$, 298 K) of **6$_{Ge}$** (+ = toluene, * = impurities).

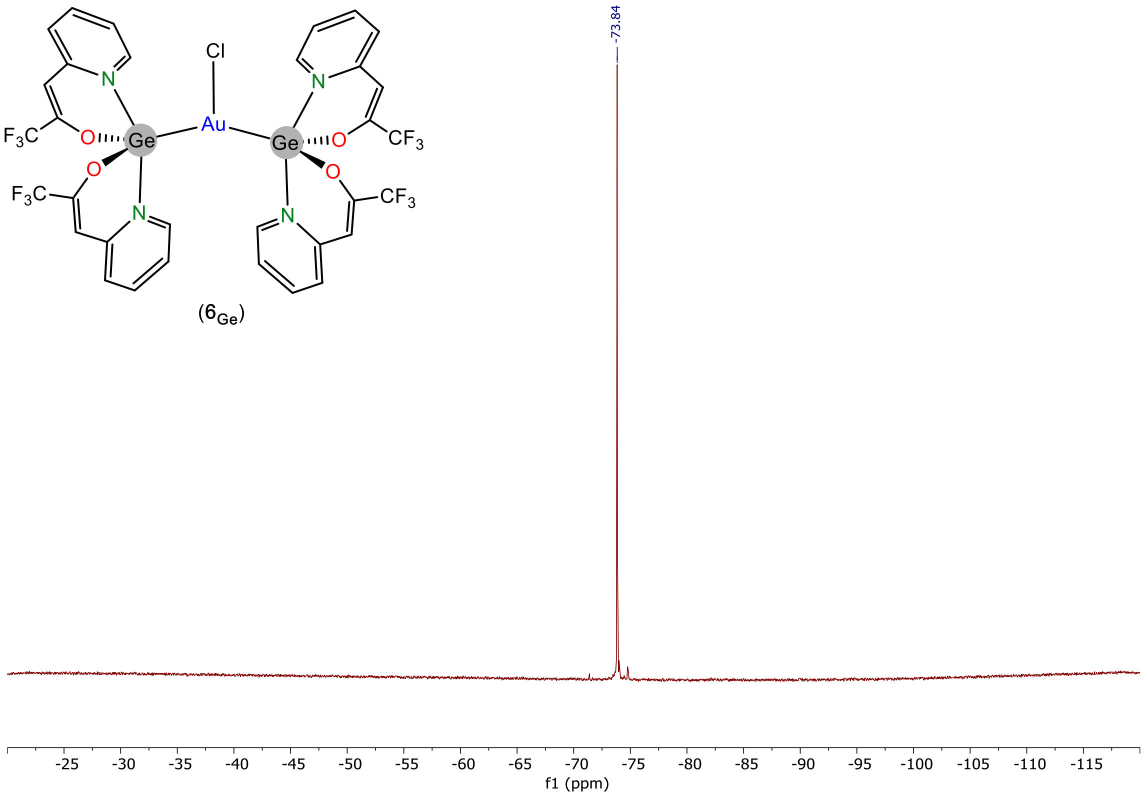

Figure S14. $^{19}F\{^{1}H\}$ (282.4 MHz) NMR spectrum ($CDCl_3$, 298 K) of $\mathbf{6_{Ge}}$.

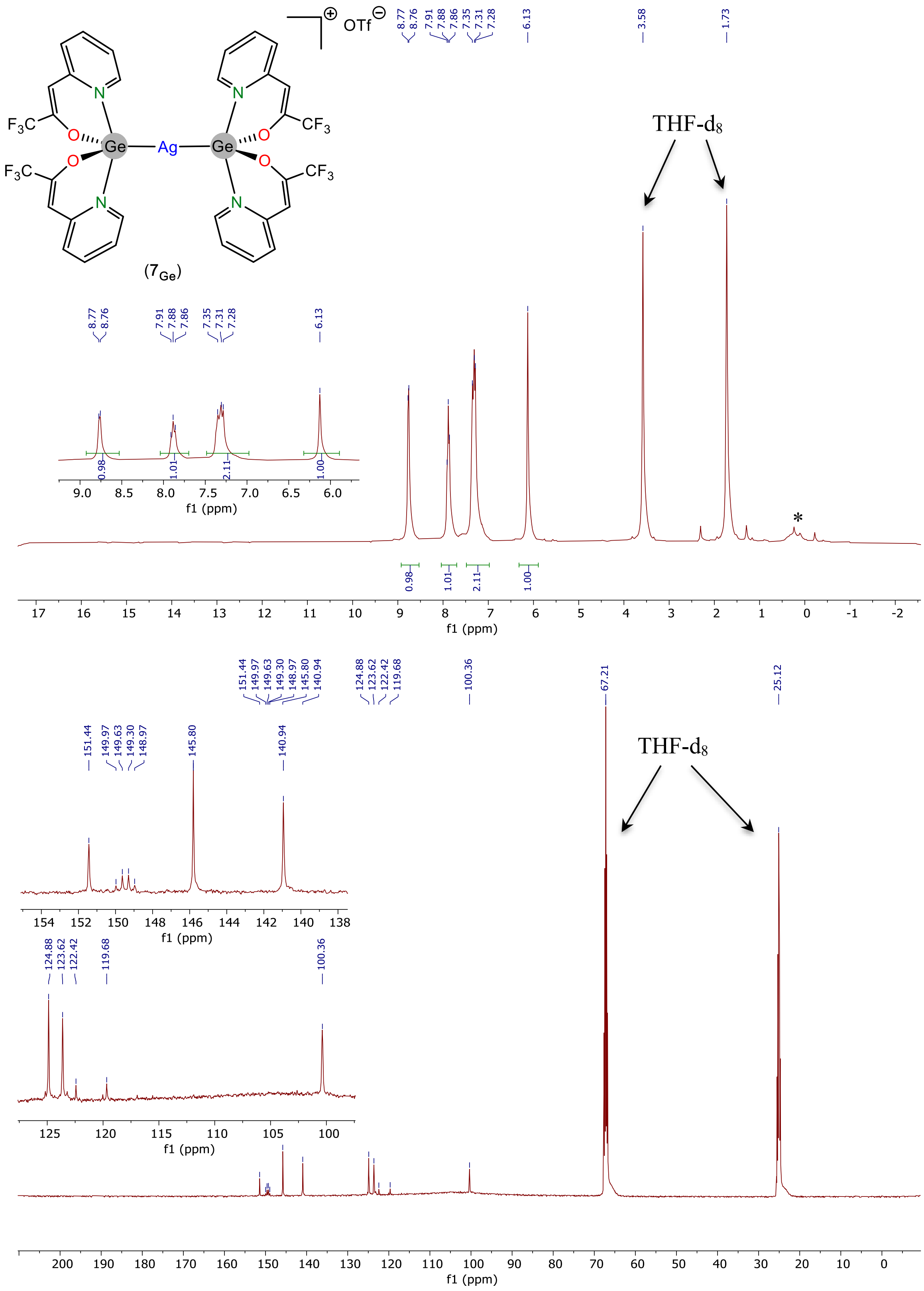

Figure S15. ^{1}H (top, 400.5 MHz) and $^{13}C\{^{1}H\}$(bottom, 100.7 MHz) NMR spectra (THF-d_8, 298 K) of $\mathbf{7_{Ge}}$ (* = impurities).

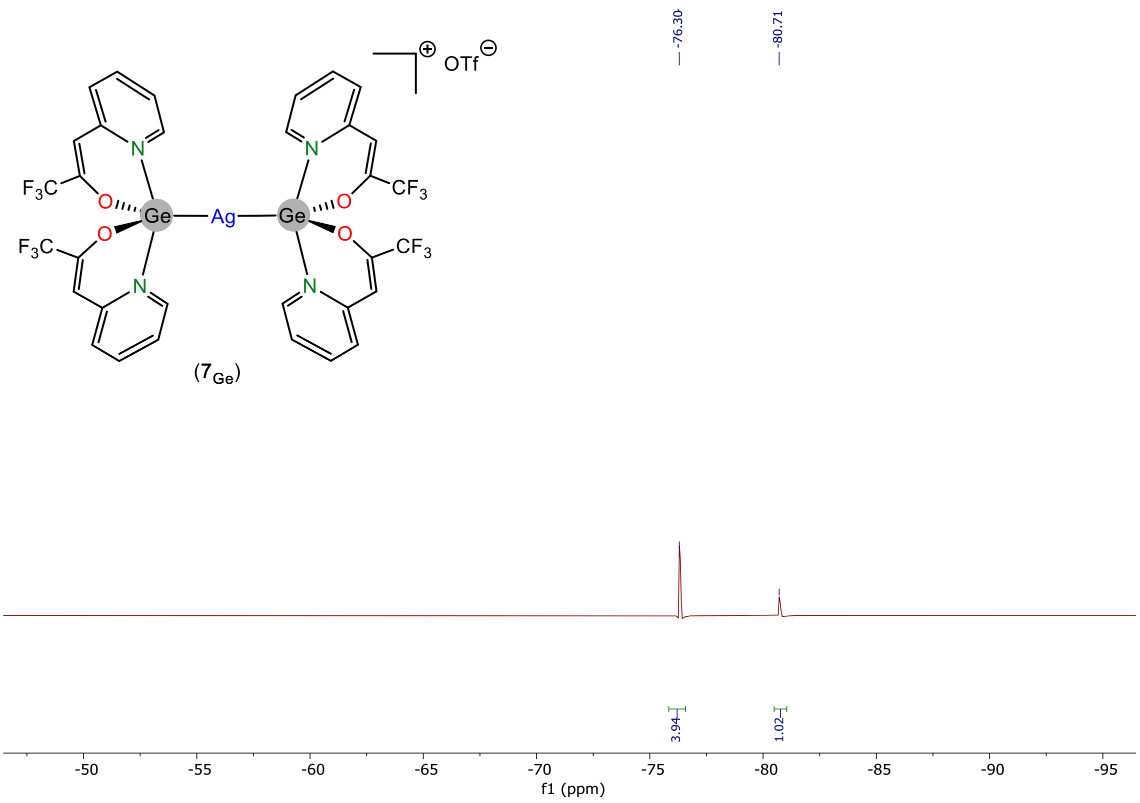

Figure S16. $^{19}F\{^{1}H\}$ (282.4 MHz) NMR spectrum (THF-d_8, 298 K) of **7_{Ge}**.

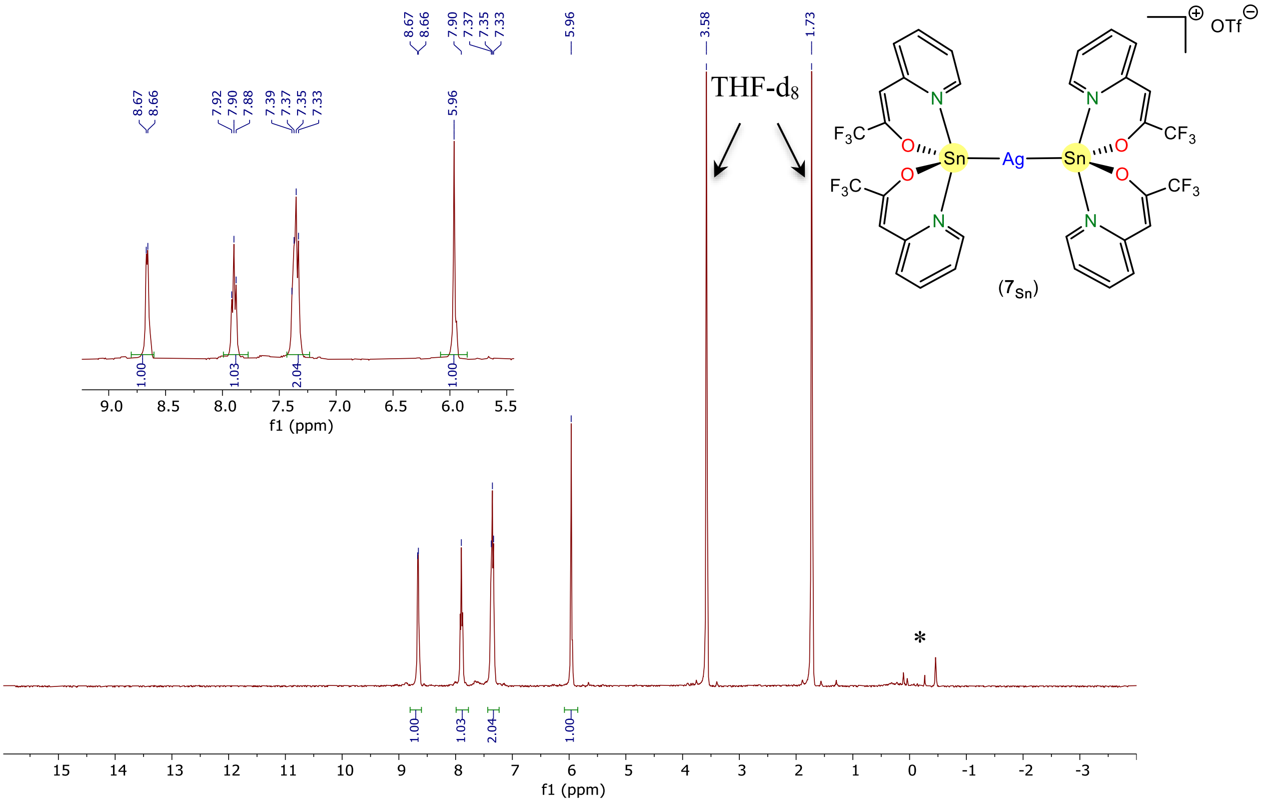

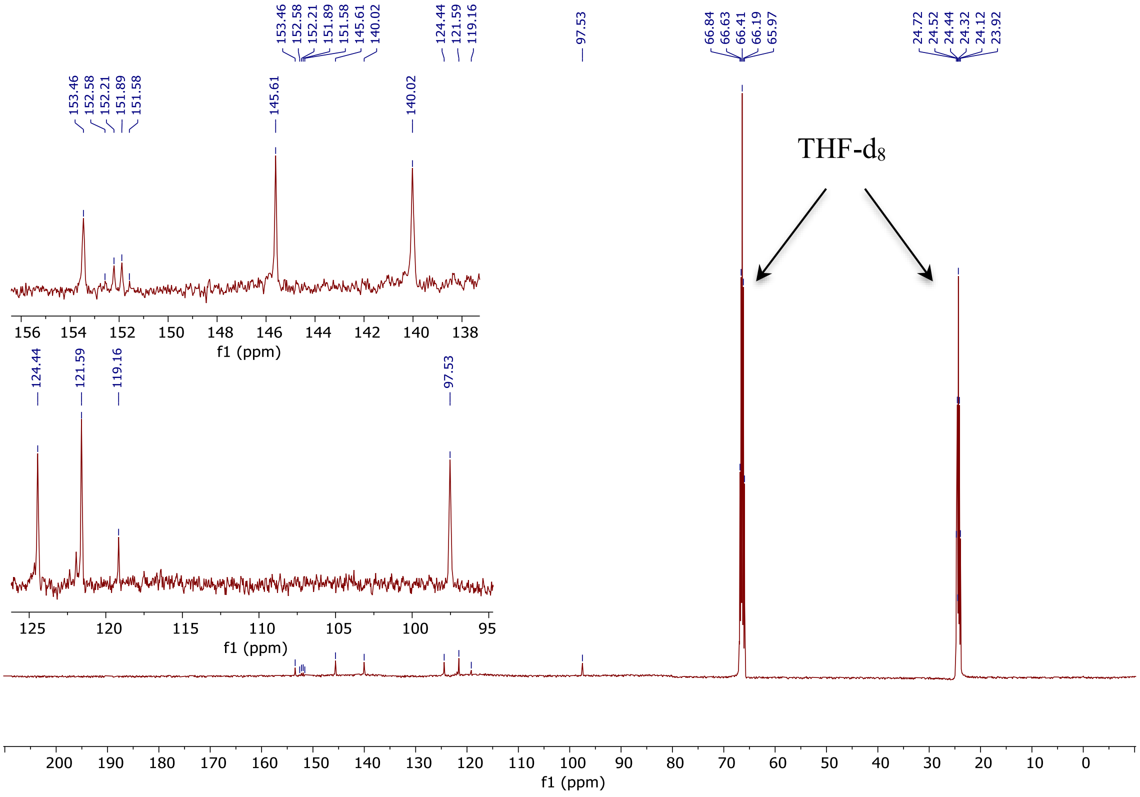

Figure S17. ^{1}H (top, 400.5 MHz) and $^{13}C\{^{1}H\}$ (bottom, 100.7 MHz) NMR spectra (THF-d_8, 298 K) of $\mathbf{7_{Sn}}$ (* = impurities).

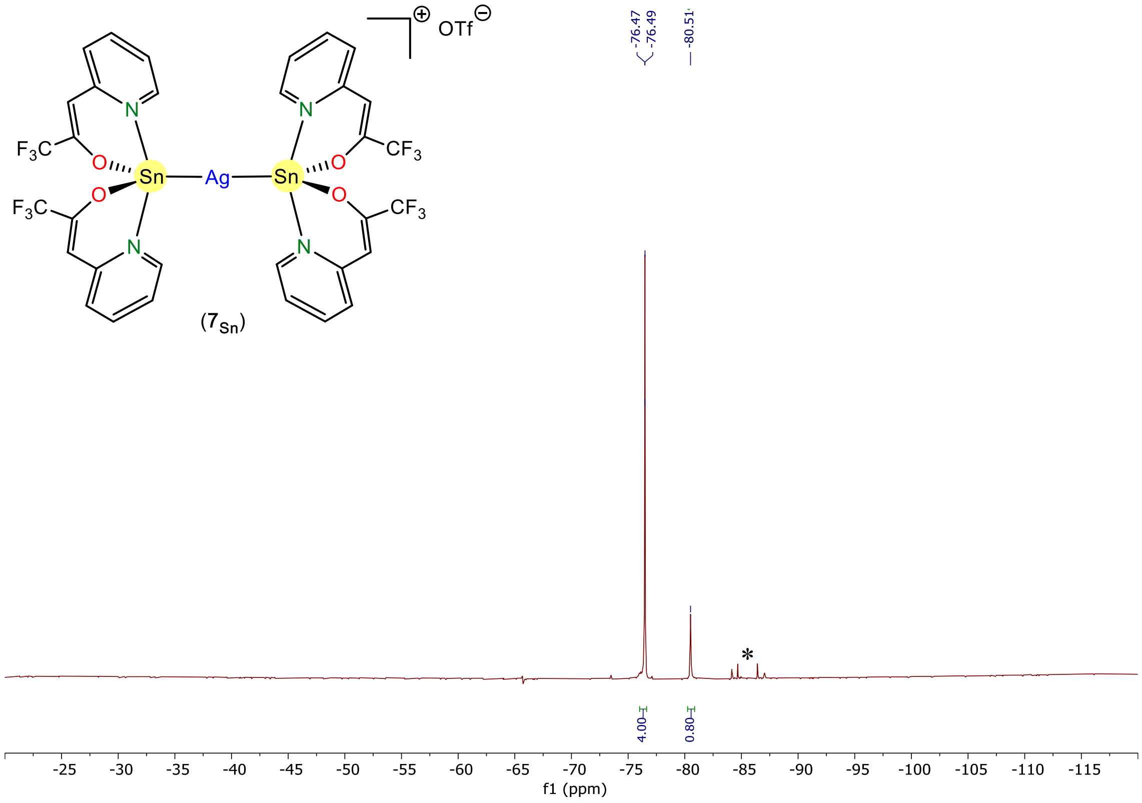

Figure S18. $^{19}F\{^{1}H\}$ (282.4 MHz) NMR spectrum (THF-d_8, 298 K) of $\mathbf{7_{Sn}}$ (* = impurities).

3. Stability Studies of $\mathbf{1_E}$ and $\mathbf{2_E}$

Air-stability of Ge(tfppO)$_2$ ($\mathbf{1_{Ge}}$): Yellow solutions of $\mathbf{1_{Ge}}$ in C_6D_6 and $CDCl_3$ were prepared in NMR tubes inside the glove box by adding a small amount of $\mathbf{1_{Ge}}$ (22 mg, 0.05 mmol; for the $CDCl_3$ solution) to *ca*. 0.3 mL of the corresponding solvent. After acquiring their ^{1}H NMR spectra under argon, the NMR tubes were opened and left to stand under air recording new ^{1}H NMR spectra after 1 (Figures S21 and S22) and 3 days (Figure S34). The air exposure led to no clear visual changes for the C_6D_6 sample and to the precipitation of a little amount of a brownish solid for the $CDCl_3$ sample.

Water-stability of Ge(tfppO)$_2$ ($\mathbf{1_{Ge}}$): Deoxygenated water (0.9 µL, 0.05 mmol) was added under argon to an NMR tube containing a yellow solution of $\mathbf{1_{Ge}}$ (prepared in the glove box by adding $\mathbf{1_{Ge}}$ (22 mg, 0.05 mmol) to *ca*. 0.3 mL of $CDCl_3$) and the resulting mixture was analyzed by ^{1}H NMR (Figure S23). The water addition led to no clear visual changes.

Air-stability of Sn(tfppO)$_2$ ($\mathbf{1_{Sn}}$): C_6D_6 and $CDCl_3$ solutions of $\mathbf{1_{Sn}}$ (yellow and orange, respectively) were prepared in NMR tubes inside the glove box by adding a small amount of $\mathbf{1_{Sn}}$ (25 mg, 0.05 mmol; for the $CDCl_3$ solution) to *ca*. 0.3 mL of the corresponding solvent. After acquiring their ^{1}H NMR spectra under argon, the NMR tubes were opened and left to stand under air recording new ^{1}H NMR spectra after 1 (Figures S24 and S25) and 3 days (Figure S34). The air exposure led to no clear visual changes for the C_6D_6 sample and to the precipitation of a little amount of a brownish solid for the $CDCl_3$ sample

Water-stability of Sn(tfppO)$_2$ ($\mathbf{1_{Sn}}$): Deoxygenated water (0.9 µL, 0.05 mmol) was added under argon to an NMR tube containing a yellow solution of $\mathbf{1_{Ge}}$ (prepared in the glove box by adding $\mathbf{1_{Sn}}$ (25 mg, 0.05 mmol) to *ca*. 0.3 mL of $CDCl_3$) and the resulting mixture was analyzed by ^{1}H NMR (Figure S26). The water exposure led to the precipitation of a certain amount of a brownish solid.

Air-stability of $\mathbf{2_{Ge}}$: Yellow solutions of $\mathbf{2_{Ge}}$ in C_6D_6 and $CDCl_3$ were prepared in NMR tubes inside the glove box by adding a small amount of $\mathbf{2_{Ge}}$ (15 mg, 0.05 mmol; for the $CDCl_3$ solution) to *ca*. 0.3 mL of the corresponding solvent. After acquiring their ^{1}H NMR spectra under argon, the NMR tubes were opened and left to stand under air recording new ^{1}H NMR spectra after 1 (Figures S27 and S28) and 3 days (Figure S34). The air exposure led to no clear visual changes for the $CDCl_3$ sample and to the precipitation of a little amount of an orange solid for the C_6D_6 sample.

Water-stability of 2_{Ge}: Deoxygenated water (0.9 μL, 0.05 mmol) was added under argon to an NMR tube containing a yellow solution of **1_{Ge}** (prepared in the glove box by adding **2_{Ge}** (15 mg, 0.05 mmol) to *ca*. 0.3 mL of $CDCl_3$) and the resulting mixture was analyzed by ^{1}H NMR (Figure S29). The water exposure led to the precipitation of an orange solid.

Air-stability of 2_{Sn}: Solutions of **2_{Sn}** in C_6D_6 and $CDCl_3$ were prepared in NMR tubes inside the glove box by adding a small amount of **2_{Sn}** (17 mg, 0.05 mmol; for the $CDCl_3$ solution) to *ca*. 0.3 mL of the corresponding solvent. Some brownish solid, remained undissolved. After acquiring their ^{1}H NMR spectra under argon, the NMR tubes were opened and left to stand under air recording new ^{1}H NMR spectra after 1 (Figures S30 and S31) and 3 days (Figure S34). The air exposure led to the precipitation of additional brownish solid in both cases.

Water-stability of 2_{Sn}: Deoxygenated water (0.9 μL, 0.05 mmol) was added under argon to an NMR tube containing a yellow solution of **2_{Sn}** (prepared in the glove box by adding **2_{Sn}** (17 mg, 0.05 mmol) to *ca*. 0.3 mL of $CDCl_3$) and the resulting mixture was analyzed by ^{1}H NMR (Figure S32). The water exposure led to the precipitation of additional orange-brownish solid.

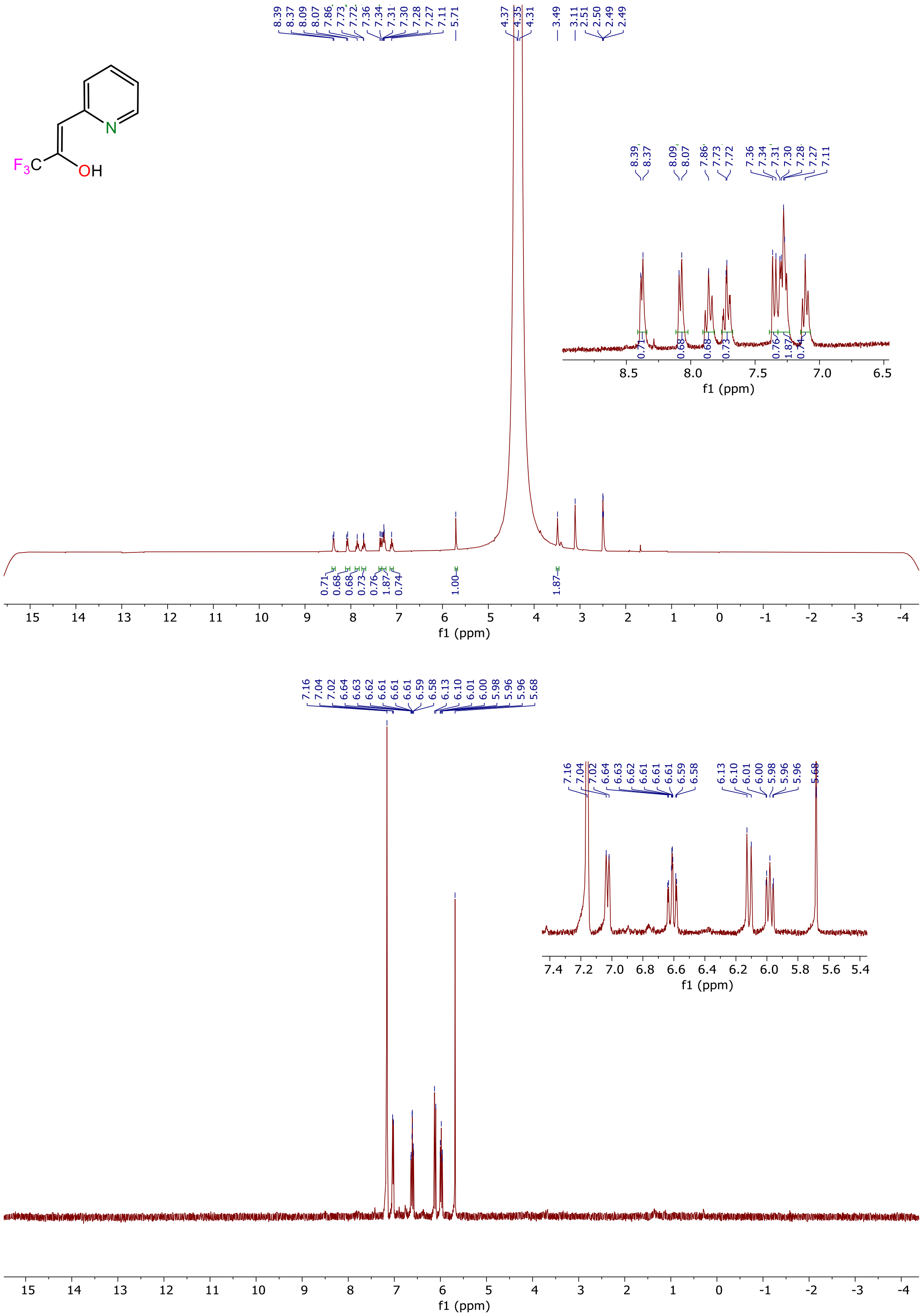

Figure S19. ^{1}H (300.1 MHz, 298 K) NMR spectra of tfppOH in dmso-d_6 (top) and in C_6D_6 (bottom).

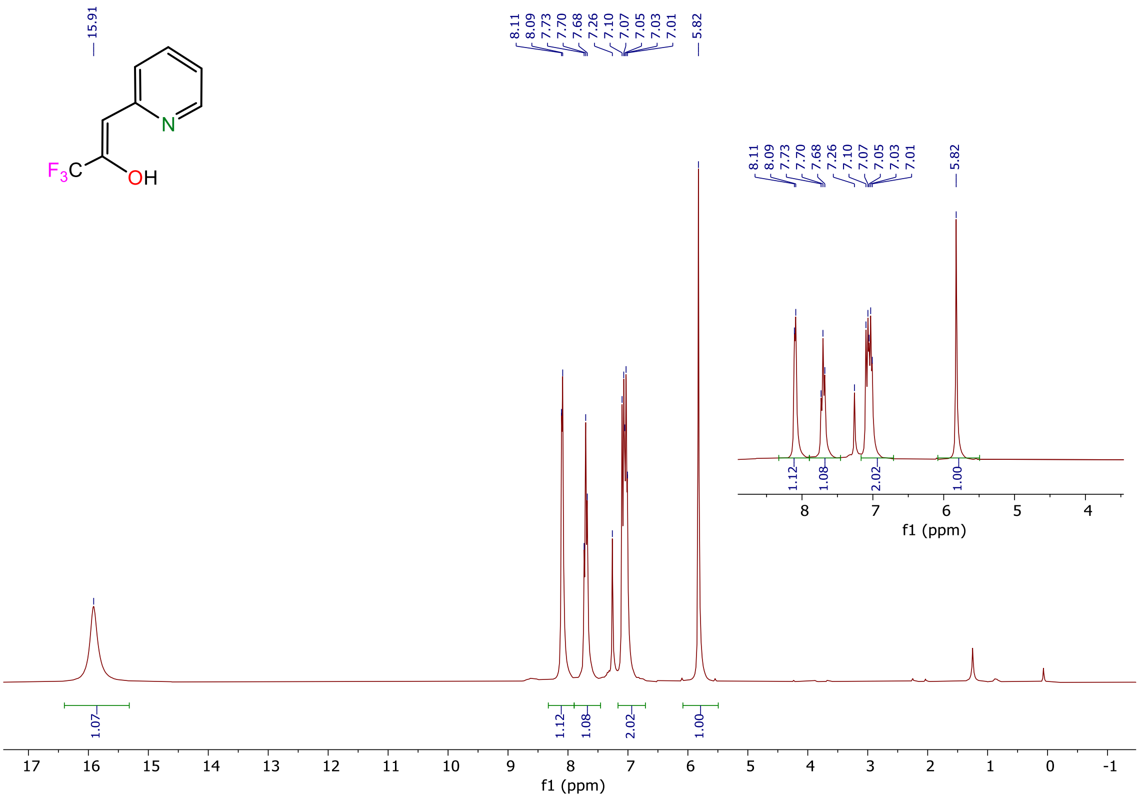

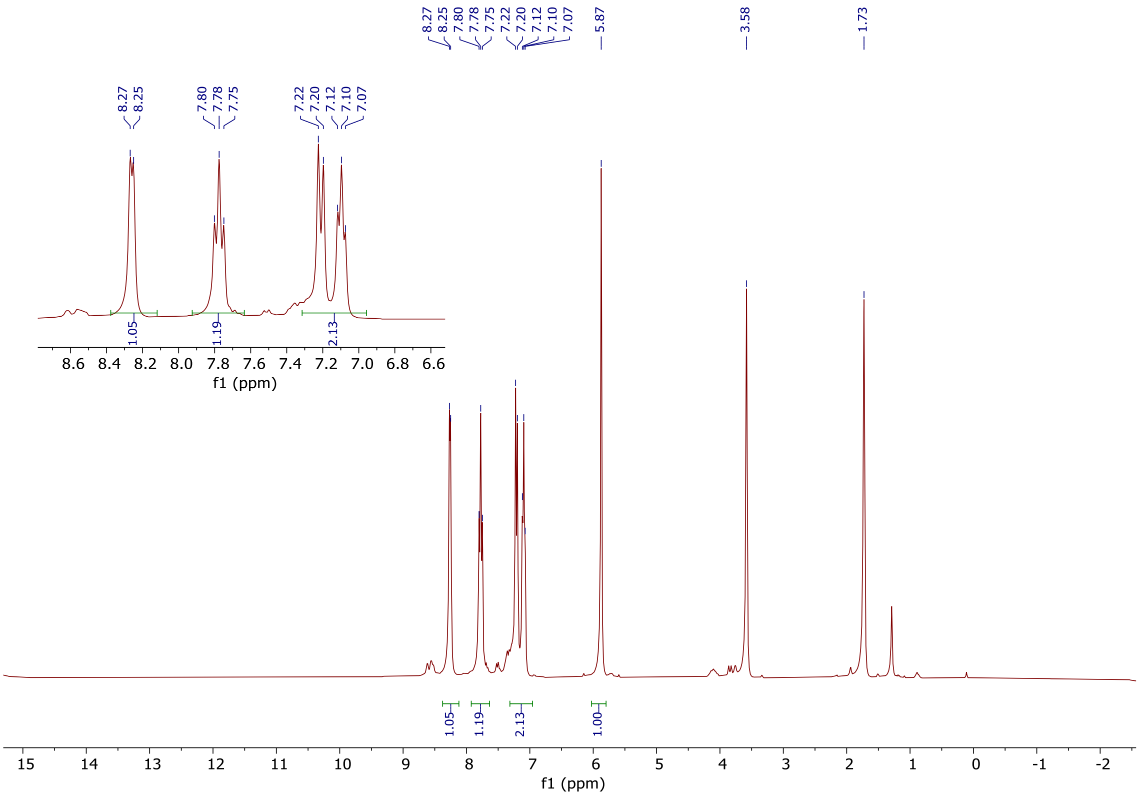

Figure S20. ^{1}H (300.1 MHz, 298 K) NMR spectra of tfppOH in $CDCl_3$ (top) and in THF-d_8 (bottom).

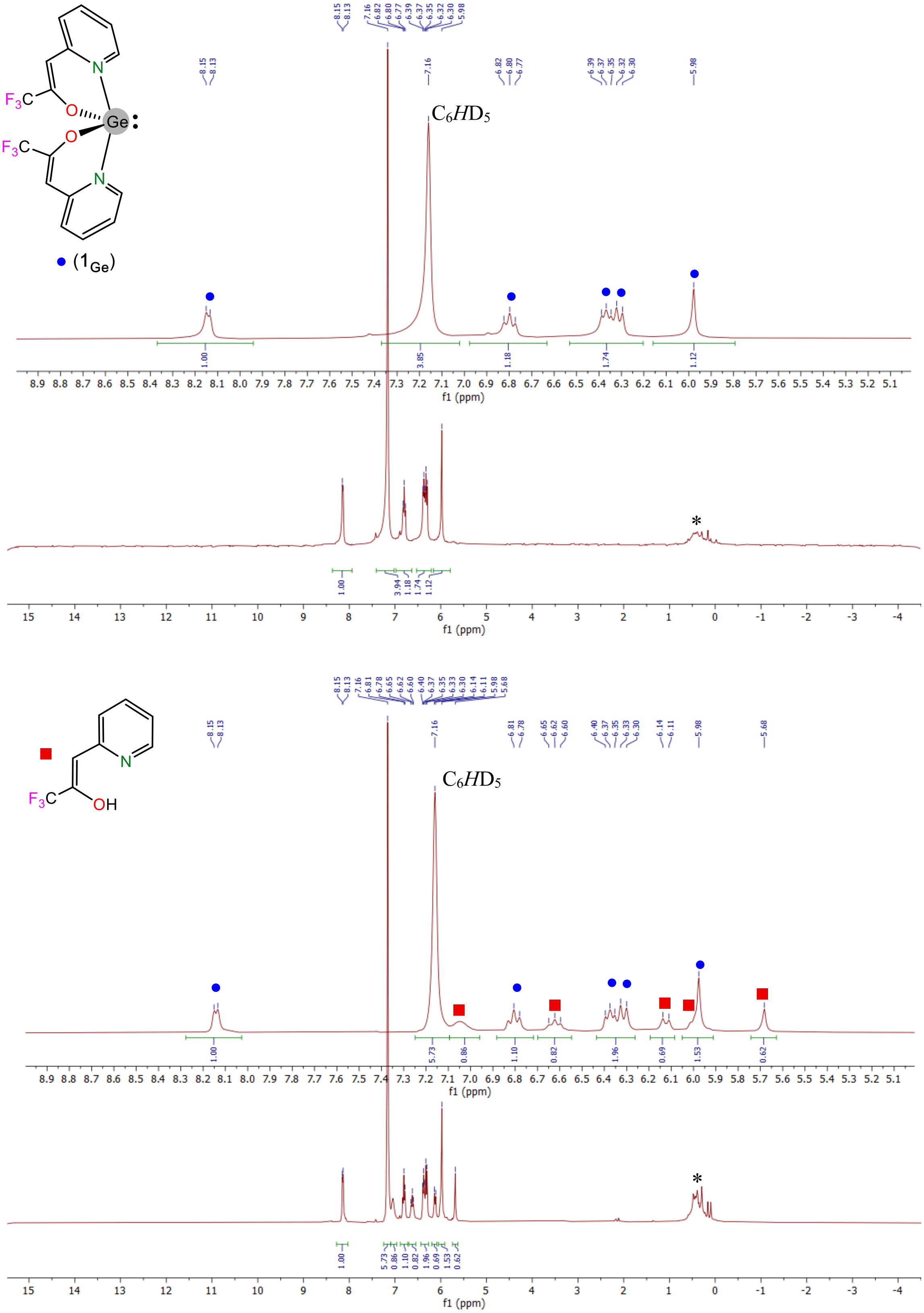

Figure S21. ^{1}H (top, 300.1 MHz) NMR spectra (C_6D_6, 298 K) of $\mathbf{1_{Ge}}$ under argon (top) and after standing in air for one day (bottom). (● = $\mathbf{1_{Ge}}$, ■ = tfppOH, * = impurities).

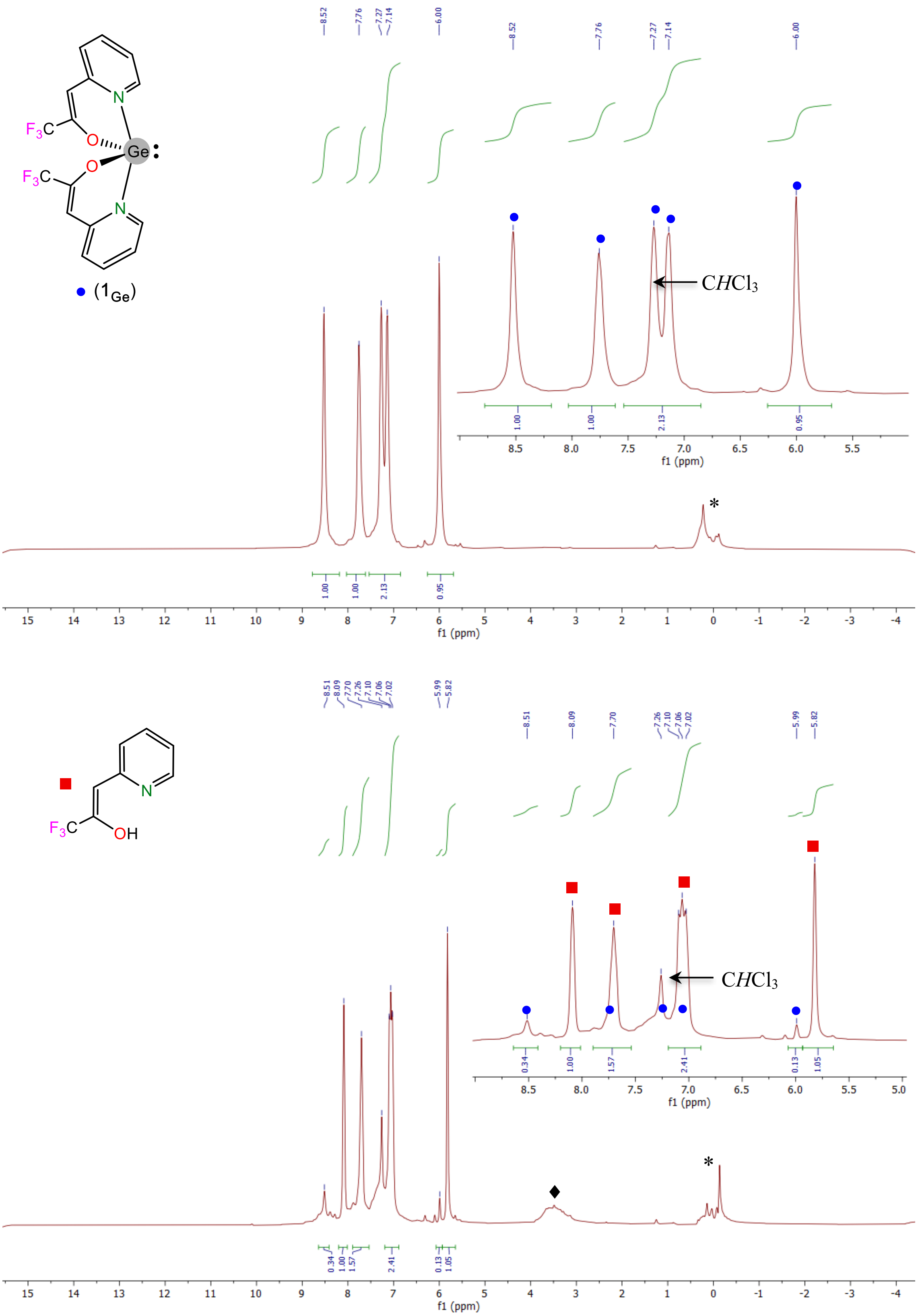

Figure S22. ^{1}H (top, 300.1 MHz) NMR spectra ($CDCl_3$, 298 K) of $\mathbf{1}_{\mathbf{Ge}}$ under argon (top) and after standing in air for one day (bottom). (● = $\mathbf{1}_{\mathbf{Ge}}$, ■ = tfppOH, ♦ = unknown new species, * = impurities).

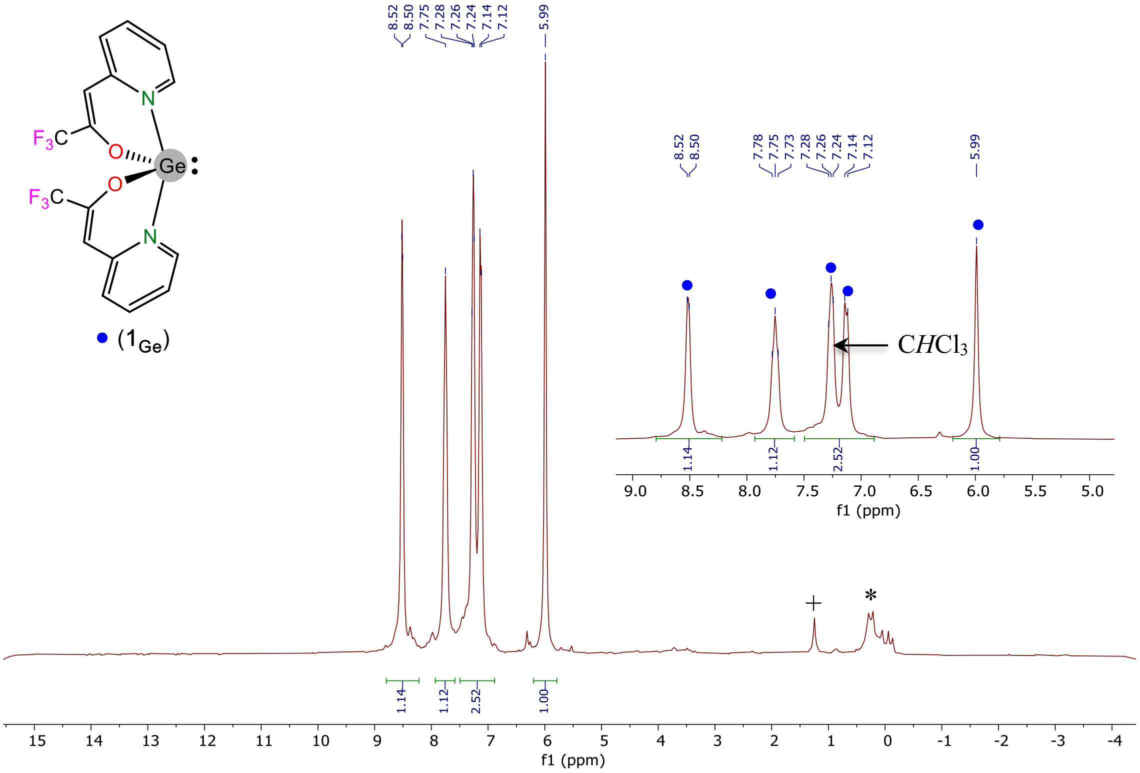

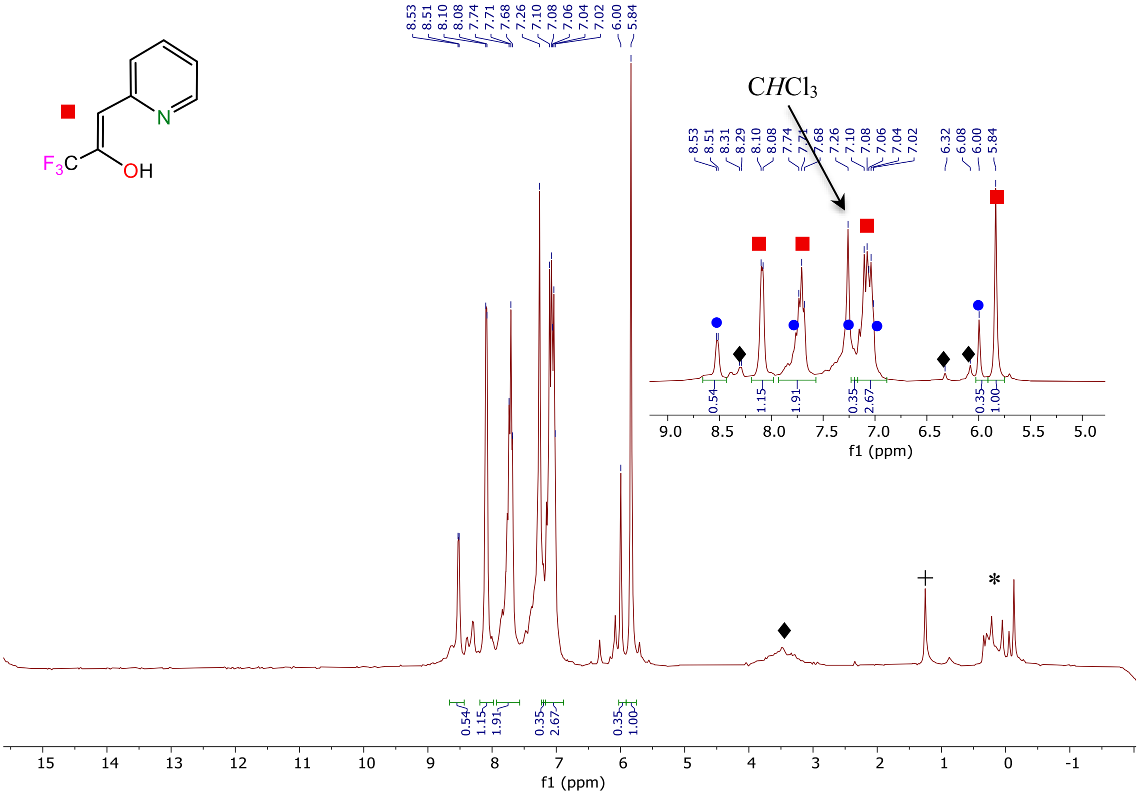

Figure S23. ^{1}H (top, 300.1 MHz) NMR spectra ($CDCl_3$, 298 K) of $\mathbf{1_{Ge}}$ before (top) and after the addition of 1 equivalent of deoxygenated water (bottom) (● = $\mathbf{1_{Ge}}$, ■ = tfppOH, ♦ = unknown new species, (+ = paraffin grease, * = impurities).

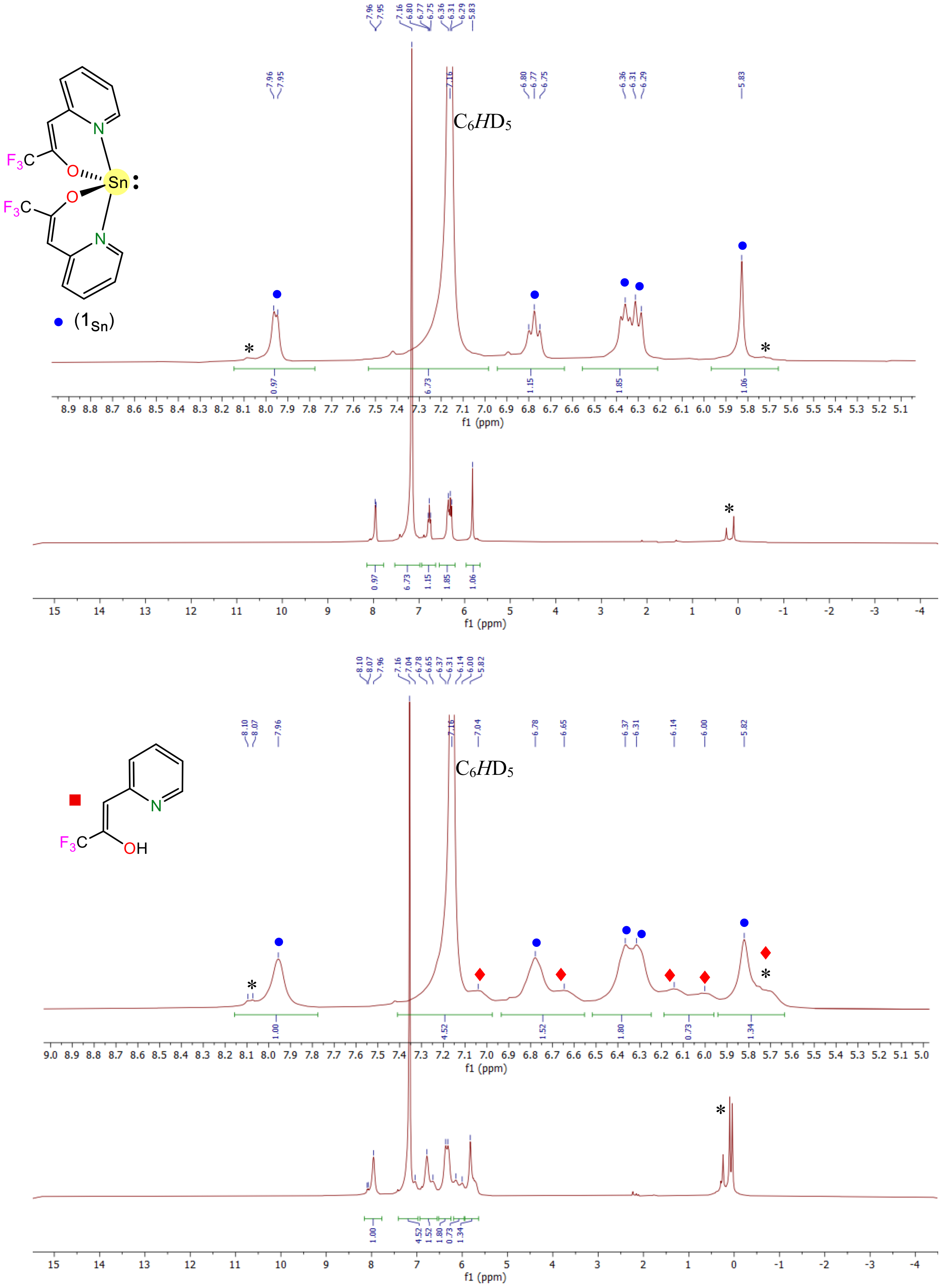

Figure S24. ^{1}H (top, 300.1 MHz) NMR spectra (C_6D_6, 298 K) of **1**$_{\mathbf{Sn}}$ under argon (top) and after standing in air for one day (bottom). (● = **1**$_{\mathbf{Sn}}$, ■ = tfppOH, * = impurities).

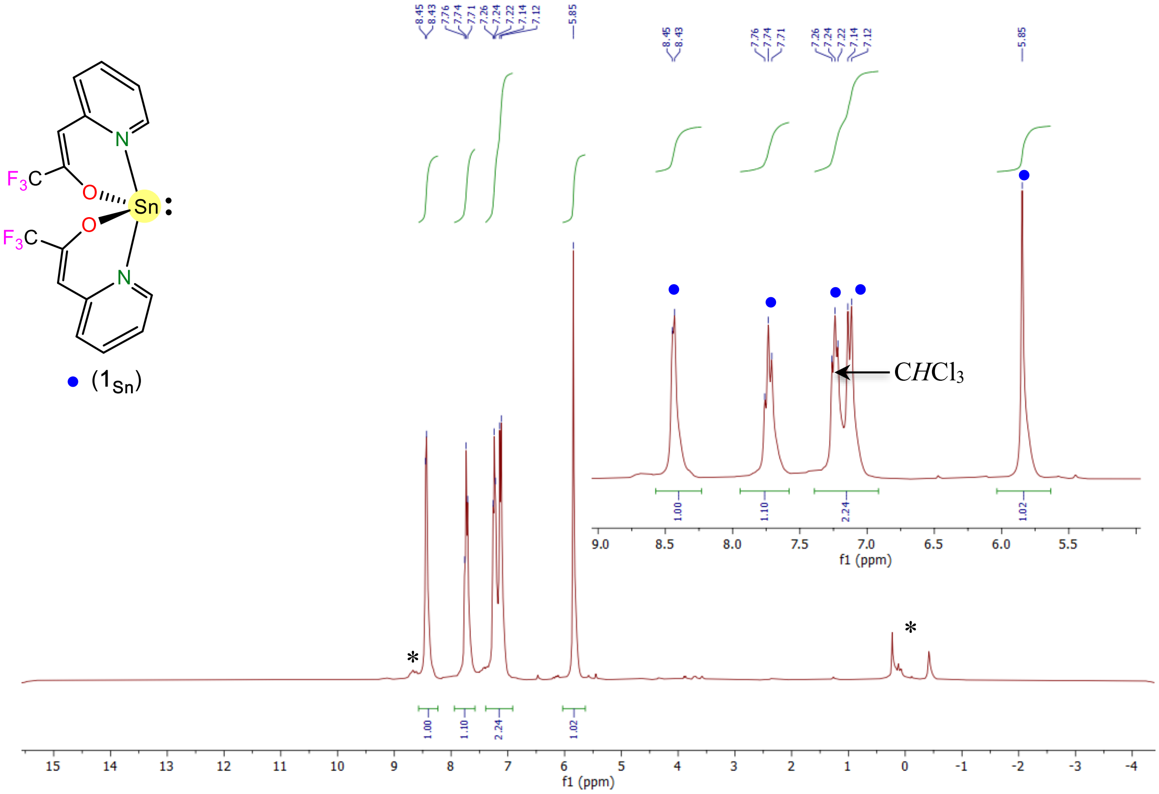

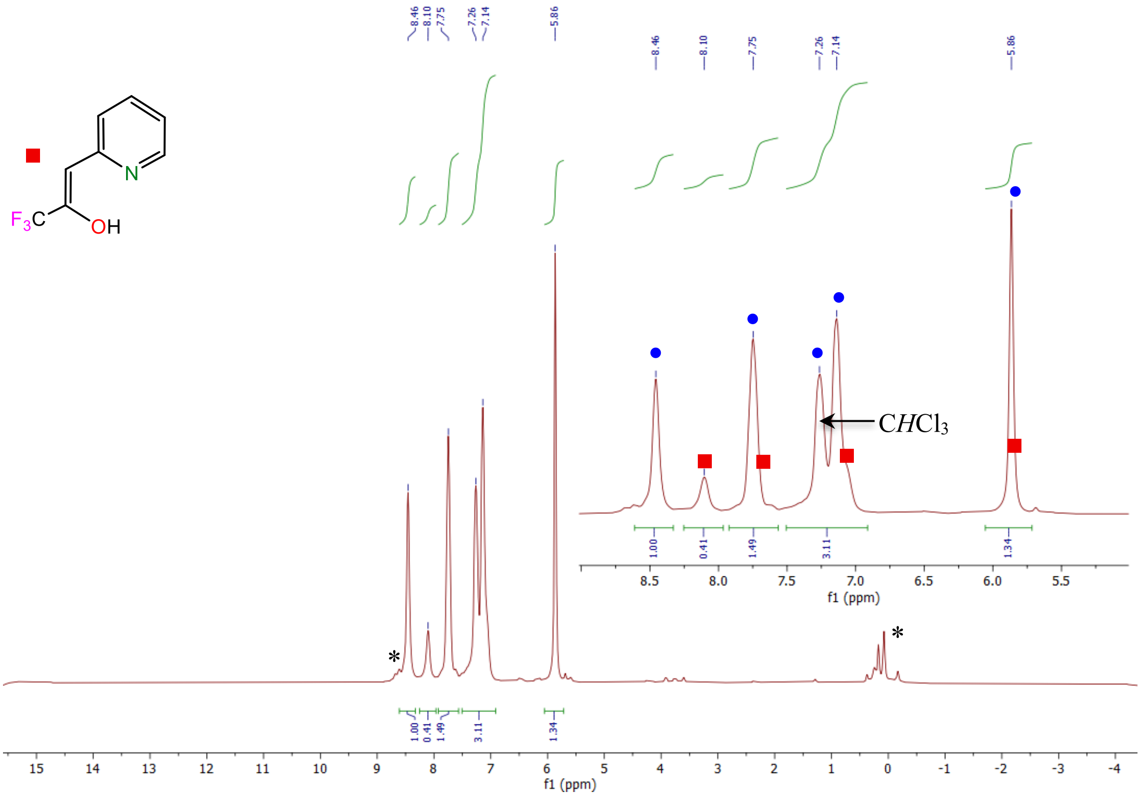

Figure S25. ^{1}H (top, 300.1 MHz) NMR spectra ($CDCl_3$, 298 K) of $\mathbf{1_{Sn}}$ under argon (top) and after standing in air for one day (bottom). (● = $\mathbf{1_{Sn}}$, ■ = tfppOH, * = impurities).

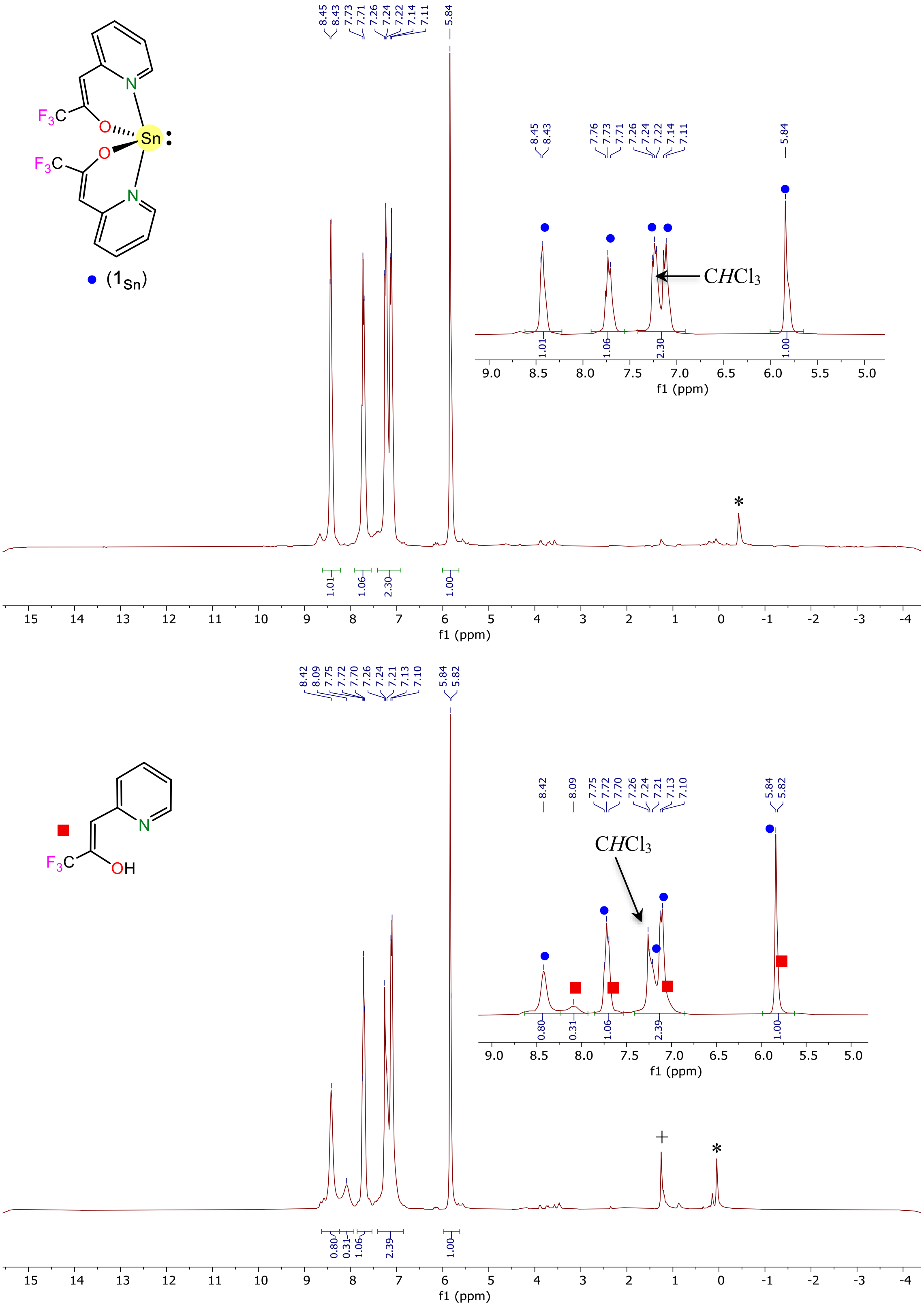

Figure S26. ^{1}H (top, 300.1 MHz) NMR spectra ($CDCl_3$, 298 K) of $\mathbf{1_{Sn}}$ before (top) and after the addition of 1 equivalent of deoxygenated water (bottom) (● = $\mathbf{1_{Sn}}$, ■ = 2-PyCHC(CF_3)OH, + = paraffin grease, * = impurities).

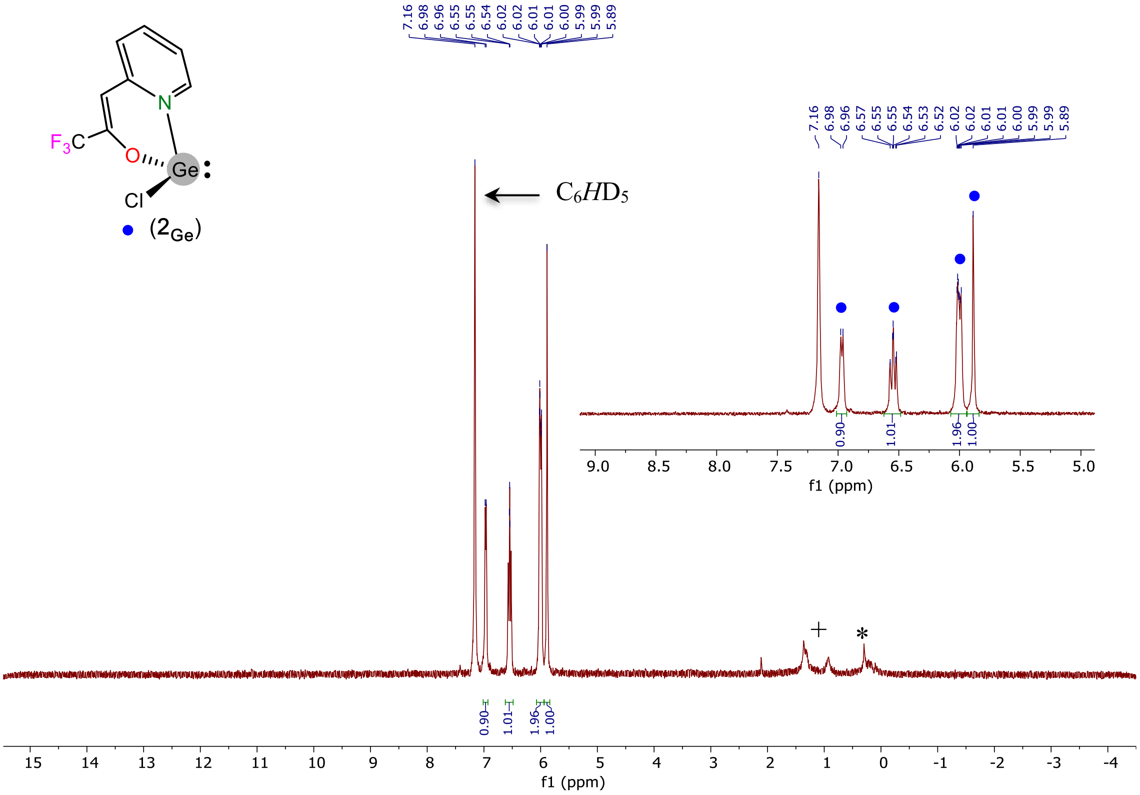

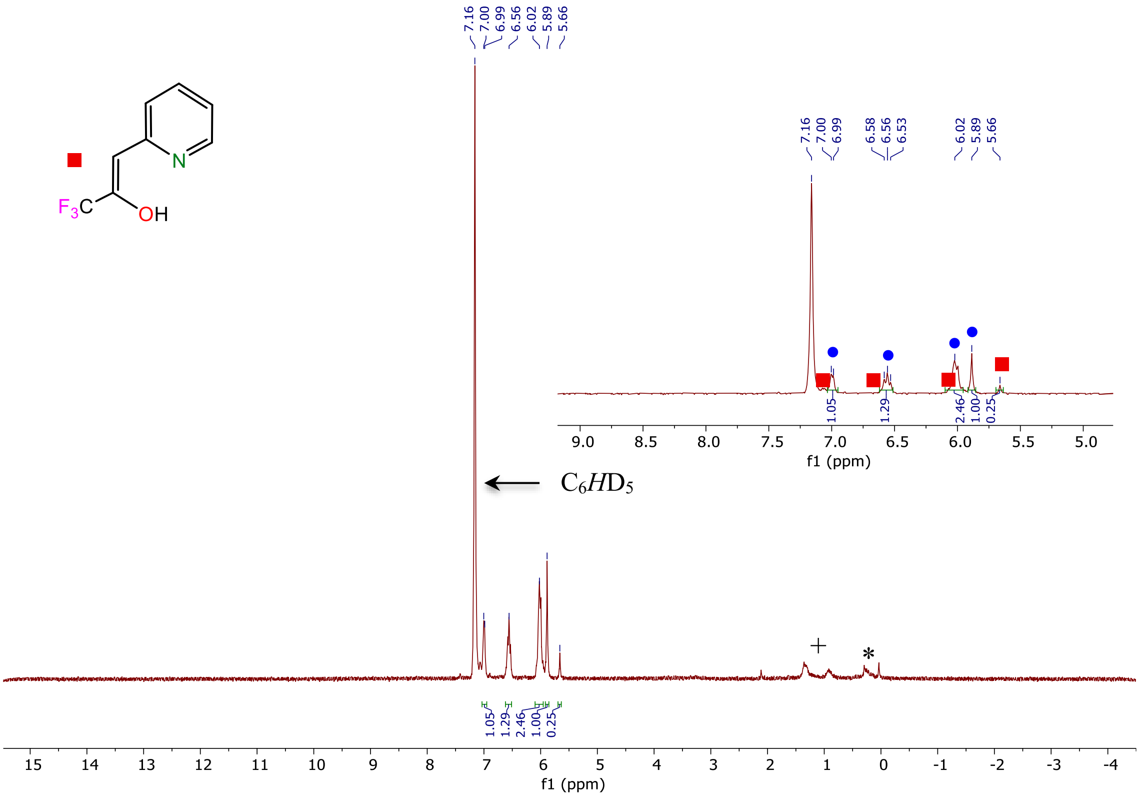

Figure S27. ^{1}H (top, 300.1 MHz) NMR spectra (C_6D_6, 298 K) of $\mathbf{2}_{\mathbf{Ge}}$ under argon (top) and after standing in air for one day (bottom). (● = $\mathbf{2}_{\mathbf{Ge}}$, ■ = tfppOH, (+ = hexane, * = impurities).

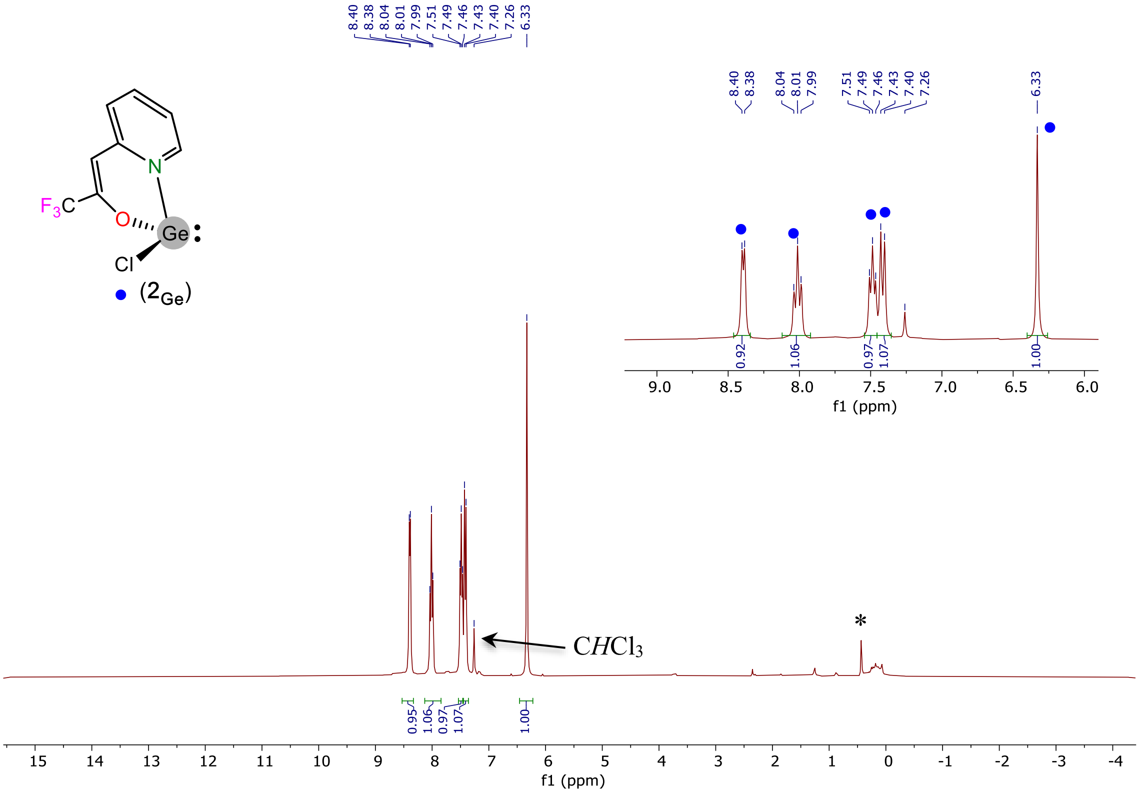

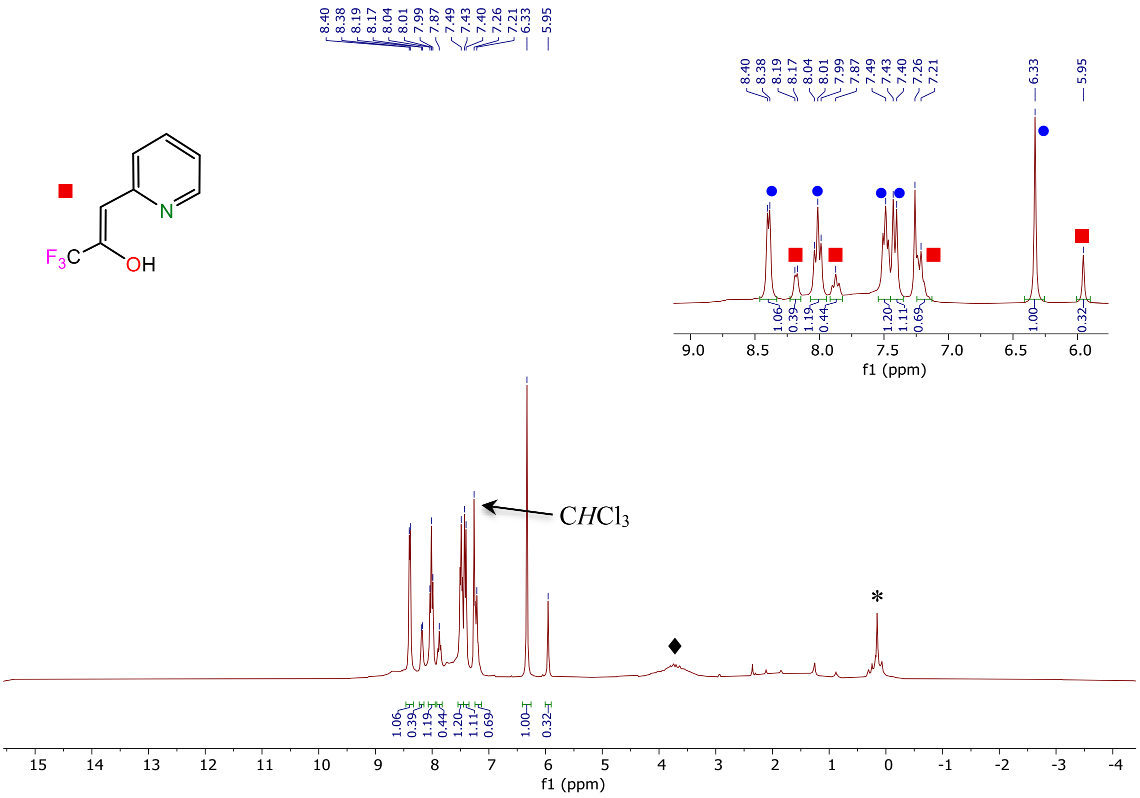

Figure S28. ^{1}H (top, 300.1 MHz) NMR spectra ($CDCl_3$, 298 K) of **2**$_{\mathbf{Ge}}$ under argon (top) and after standing in air for one day (bottom). (● = **2**$_{\mathbf{Ge}}$, ■ = tfppOH, ♦ = unknown new species, * = impurities).

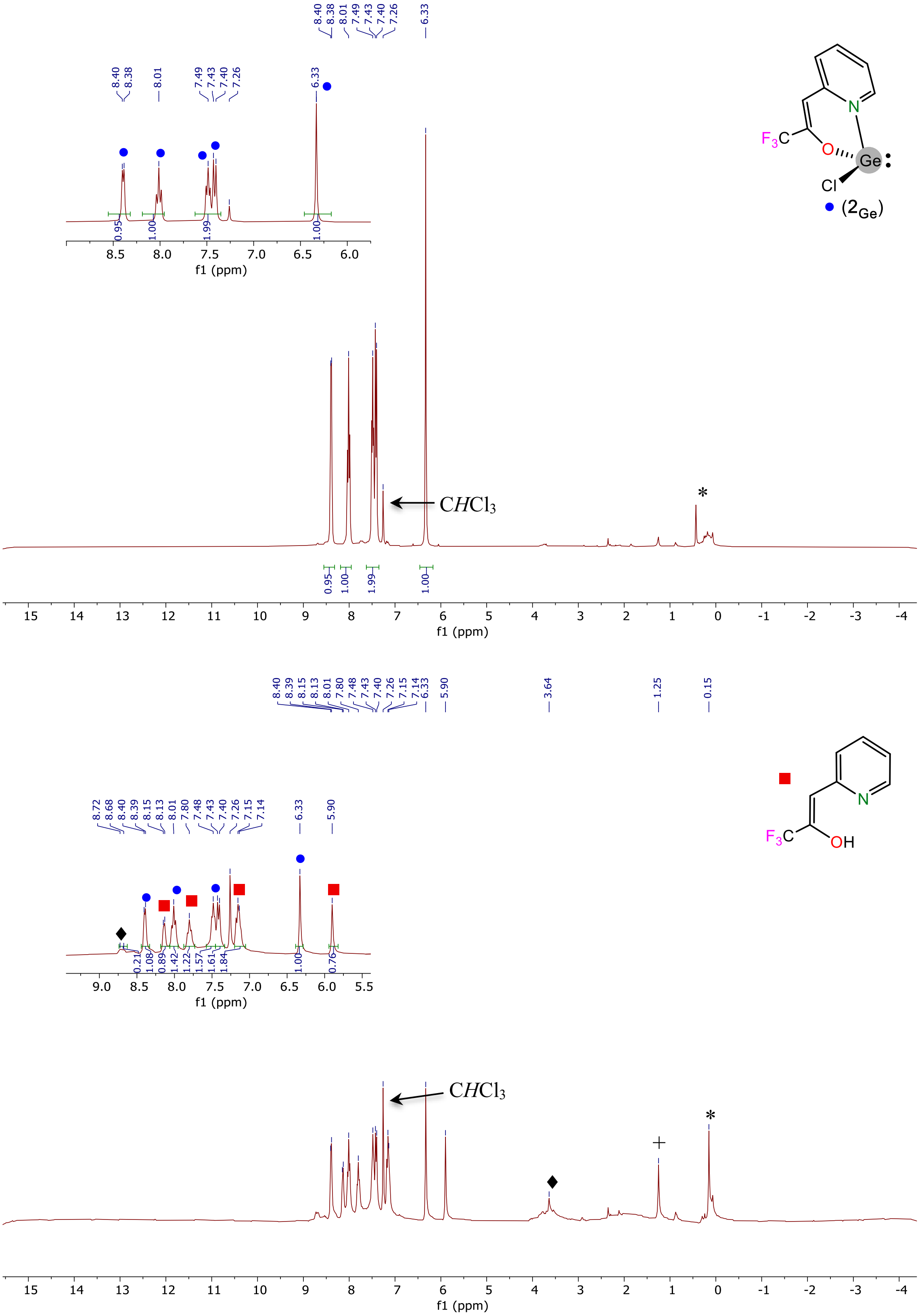

Figure S29. ^{1}H (top, 300.1 MHz) NMR spectra ($CDCl_3$, 298 K) of $\mathbf{2_{Ge}}$ before (top) and after the addition of 1 equivalent of deoxygenated water (bottom). (● = $\mathbf{2_{Ge}}$, ■ = tfppOH, ♦ = unknown new species, + = paraffin grease, * = impurities).

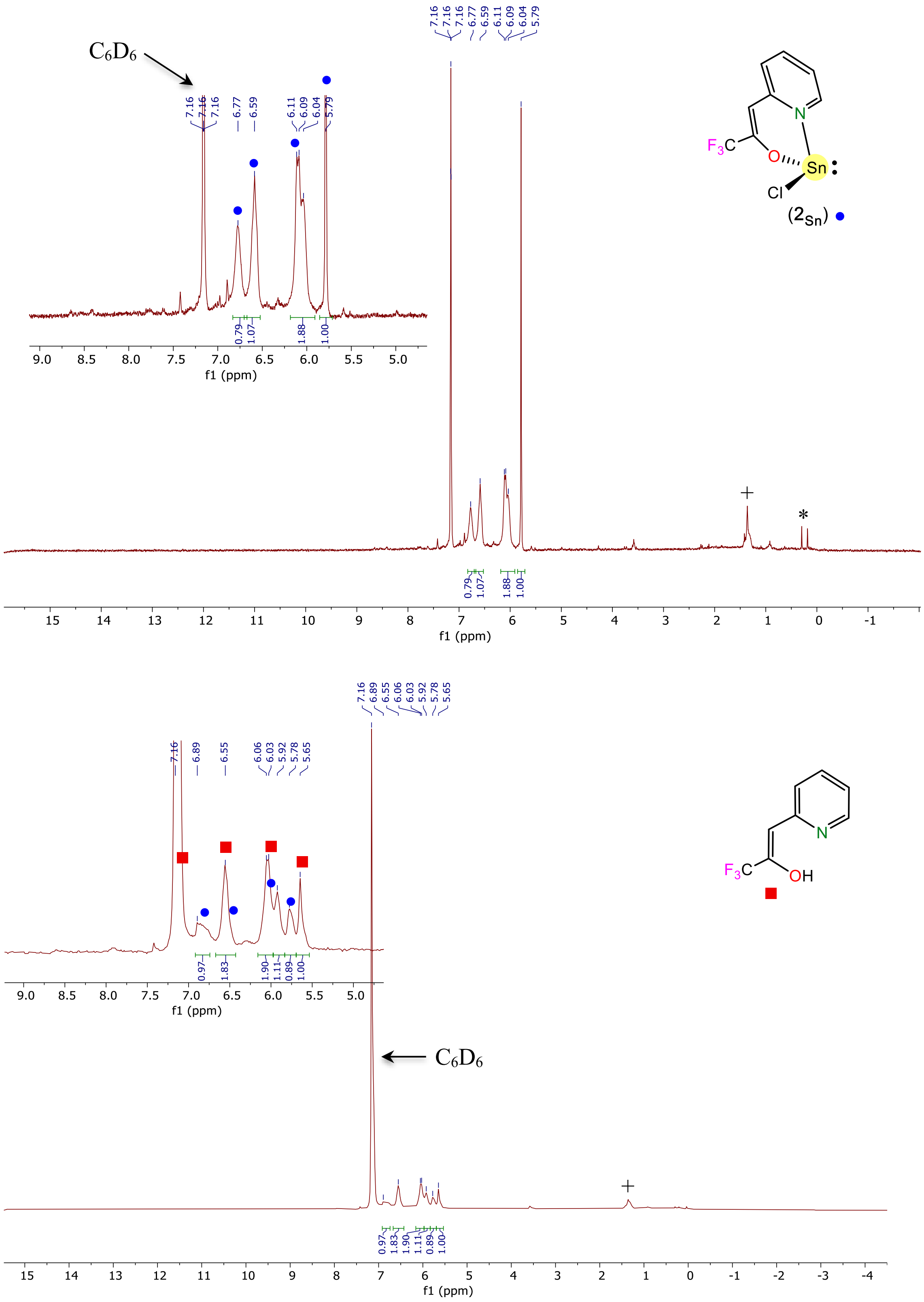

Figure S30. ^{1}H (top, 300.1 MHz) NMR spectra (C_6D_6, 298 K) of $\mathbf{2_{Sn}}$ under argon (top) and after standing in air for one day (bottom). (● = $\mathbf{2_{Sn}}$, ■ = tfppOH, + = paraffin grease, * = impurities).

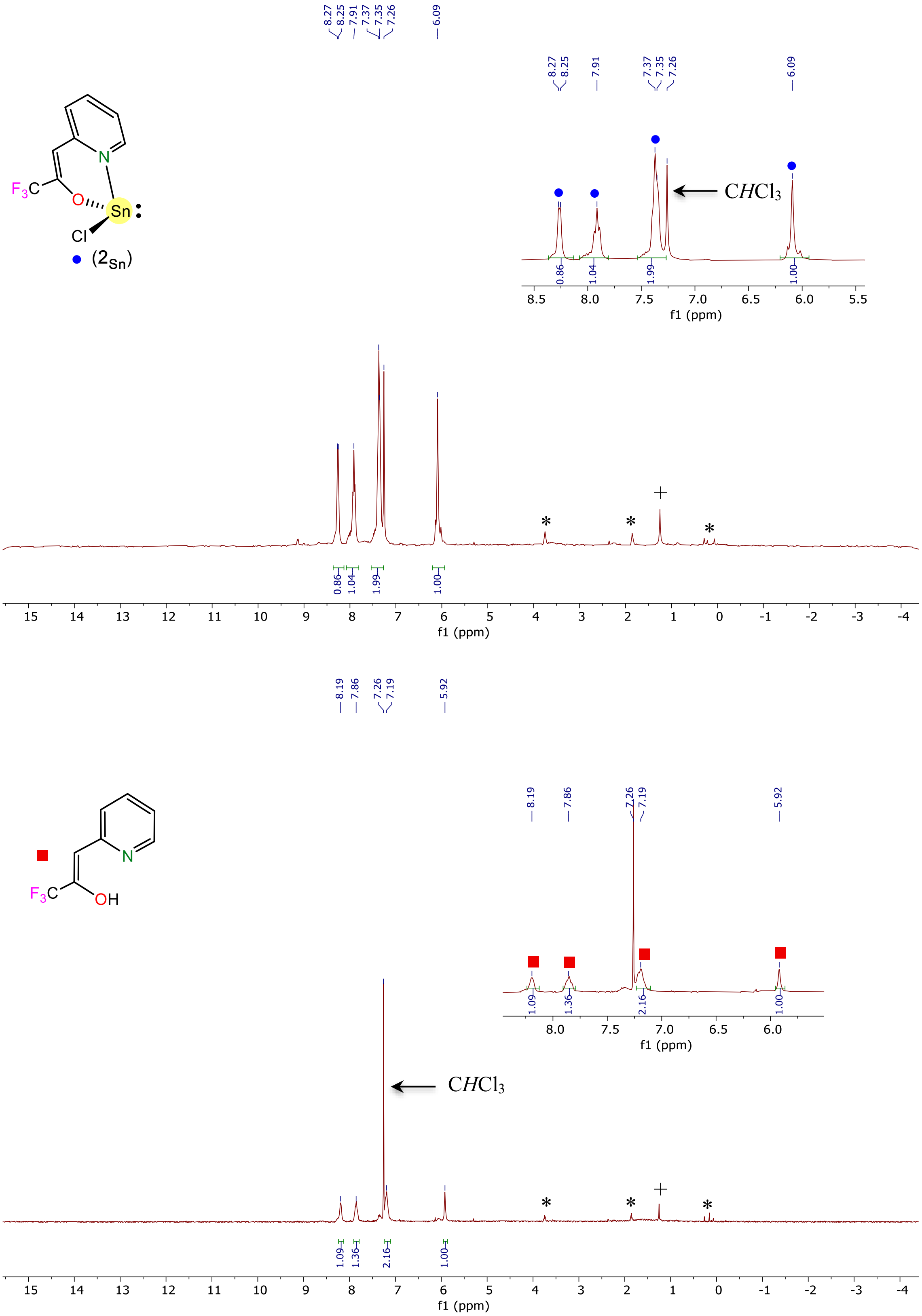

Figure S31. ^{1}H (top, 300.1 MHz) NMR spectra ($CDCl_3$, 298 K) of **2**$_{\mathbf{Sn}}$ under argon (top) and after standing in air for one day (bottom). (● = **2**$_{\mathbf{Sn}}$, ■ = tfppOH, + = paraffin grease, * = impurities).

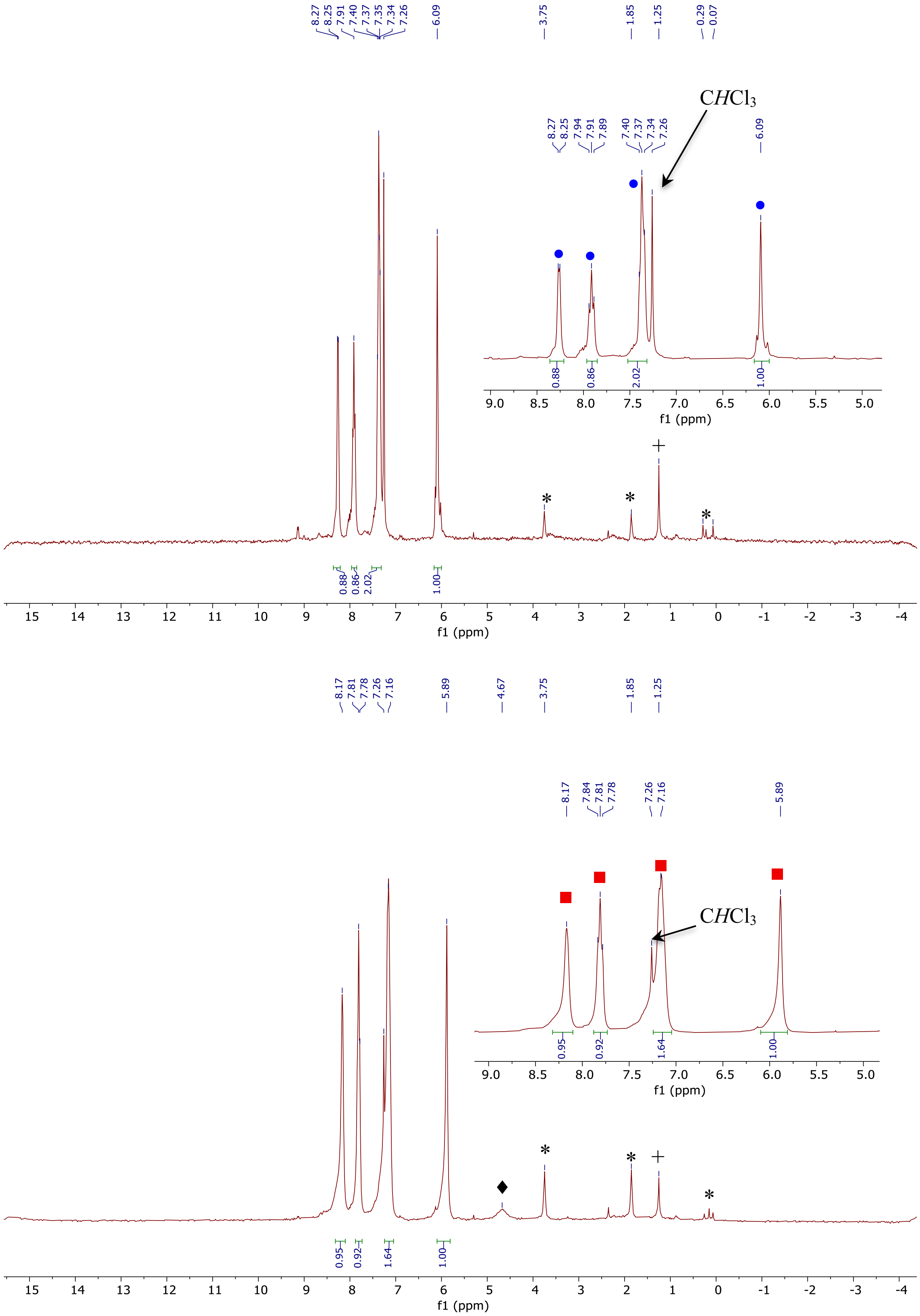

Figure S32. ^{1}H (top, 300.1 MHz) NMR spectra ($CDCl_3$, 298 K) of $\mathbf{2_{Sn}}$ before (top) and after the addition of 1 equivalent of deoxygenated water (bottom). (● = $\mathbf{2_{Sn}}$, ■ = tfppOH, ♦ = unknown new species, + = paraffin grease, * = impurities).

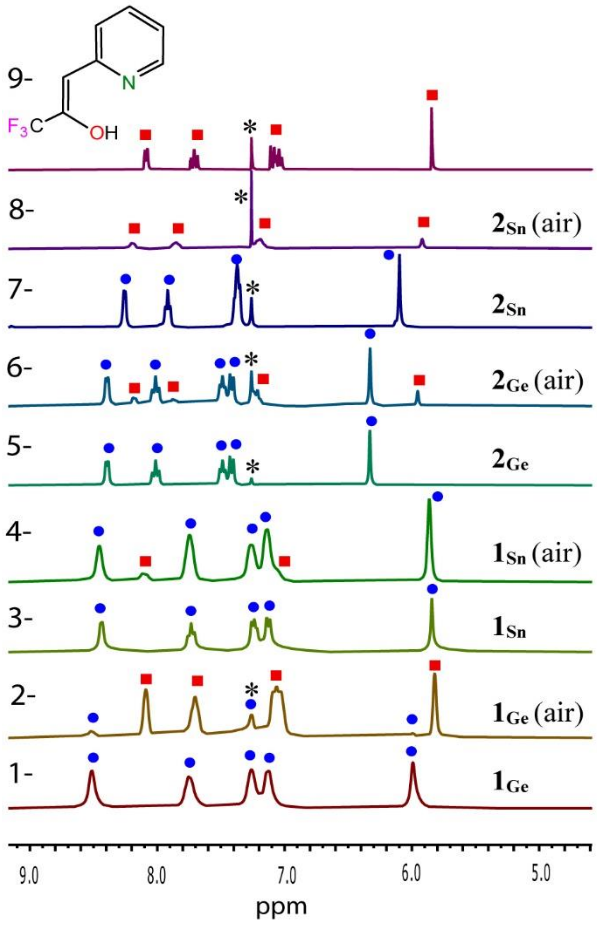

Figure S33. ^{1}H NMR spectra (298 K) of $CDCl_3$ solutions of $\mathbf{1_E}$ and $\mathbf{2_E}$ under argon (entries 1, 3, 5 and 7) and after standing in air for one day (entries 2, 4, 6 and 8). The ^{1}H NMR spectrum ($CDCl_3$, 298 K) of tfppOH is shown in entry 9. Signals belonging to $\mathbf{1_E}$ and $\mathbf{2_E}$ are marked with blue circles. Signals belonging to tfppOH are marked with red squares. * = solvent residual signal

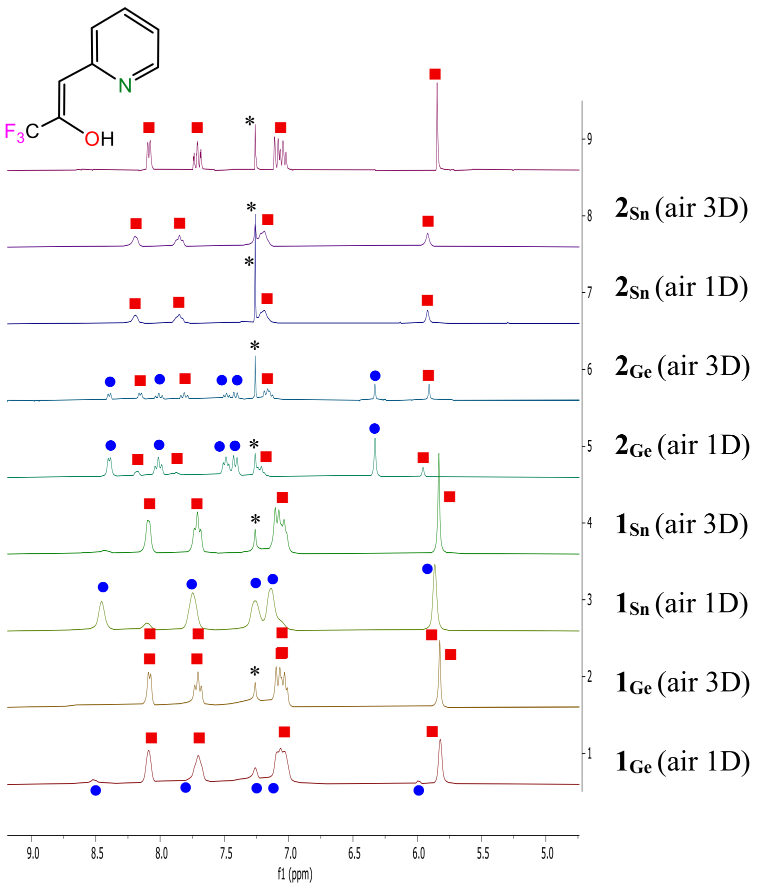

Figure S34. ^{1}H NMR spectra ($CDCl_3$, 298 K) of $\mathbf{1}_E$ and $\mathbf{2}_E$ (blue dots) after standing in air for one day (entries 1, 3, 5 and 7) and for 3 days (entries 2, 4, 6 and 8). The ^{1}H NMR spectrum ($CDCl_3$, 298 K) of tfppOH is shown in entry 9. Signals belonging to $\mathbf{1}_E$ and $\mathbf{2}_E$ are marked with blue circles. Signals belonging to tfppOH are marked with red squares. * = solvent residual signal

4. Stability Studies of 1_E and 2_E (in the presence of an internal standard)

General procedure to study the atmospheric air-stability of 1_E and 2_E in $CDCl_3$ solution in the presence of an internal standard: Solutions of the corresponding HT in $CDCl_3$ were prepared in NMR tubes inside the glove box by adding 0.05 mmol of each HT (22 mg (**1_{Ge}**), 25 mg (**1_{Sn}**), 15 mg (**2_{Ge}**) or 17 mg (**2_{Sn}**)) and fluorene as internal standard (8 mg, 0.05 mmol) to *ca.* 0.3 mL of $CDCl_3$. After acquiring their ^1H NMR spectra under argon, the NMR tubes were opened and left to stand under air recording new ^1H NMR spectra after 1,3 and 7 days (Figures S35 and 36 (**1_{Ge}**), 40 and 41 (**1_{Sn}**), 45 and 46 (**2_{Ge}**) and 50 and 51(**2_{Sn}**)). The NMR tubes were shaken to ensure homogeneity of the resultant solutions ca. 1 h before each NMR acquisition. The data utilized to calculate the percentages of remaining HT and of the formed hydrolytic product tfppOH are given in Tables S1 and S2. The air exposure led to the precipitation of a little amount of a brownish solid for the **1_{Ge}**, **1_{Sn}** and **2_{Ge}** samples and to a color change, from colorless to yellowish, of the supernatant solution of the **2_{Sn}** sample.

General procedure to study the dry air-stability of 1_E and 2_E in $CDCl_3$ solution in the presence of an internal standard: Solutions of the corresponding HT in $CDCl_3$ were prepared in NMR tubes inside the glove box by adding 0.05 mmol of each HT (22 mg (**1_{Ge}**), 25 mg (**1_{Sn}**), 15 mg (**2_{Ge}**) or 17 mg (**2_{Sn}**)) and fluorene (8 mg, 0.05 mmol) to *ca.* 0.3 mL of $CDCl_3$. After acquiring their ^1H NMR spectra under argon, the four NMR tubes were opened inside of an argon purged Schlenk flask containing a bed of fresh P_2O_5. Then, the flask was open to air connecting a drying tube filled with fresh P_2O_5 to the flask mouth (see Figure S55) recording new ^1H NMR spectra after 1, 4 and 7 days (Figures S37 and 38 (**1_{Ge}**), 42 and 43 (**1_{Sn}**), 47 and 48 (**2_{Ge}**) and 52 and 53(**2_{Sn}**)). No phosphoric acid formation was observed on top of the P_2O_5 contained inside of the Schlenk flask. The NMR tubes were shaken to ensure homogeneity of the resultant solutions ca. 1 h before each NMR acquisitions. The data utilized to calculate the percentages of remaining HT and of the formed hydrolytic product tfppOH are given in Tables S1 and S2. The air exposure led to no clear visual changes for the **2_{Sn}** sample and to the precipitation of a little amount of a brownish solid for the **1_{Sn}**, **1_{Ge}** and **2_{Ge}** samples.

General procedure to study the air-stability of 1_E and 2_E as solid-samples in the presence of an internal standard: Solid samples of the corresponding HT were weighed in glass vials inside the glove box by adding 0.05 mmol of each HT (22 mg (**1_{Ge}**), 25 mg

(**1**$_{\mathbf{Sn}}$), 15 mg (**2**$_{\mathbf{Ge}}$) or 17 mg (**2**$_{\mathbf{Sn}}$)). The vials were taken outside of the glove box, opened and left to stand under air for 1 day. The air exposure led to no clear visual changes for the solid samples. Then, inside of the glove box, fluorene (8 mg, 0.05 mmol) and *ca*. 0.3 mL of $CDCl_3$ were added to each vial and the resulting solutions/suspensions were placed inside of NMR tubes to acquire a ^{1}H NMR spectrum for each sample (Figures S39 (**1**$_{\mathbf{Ge}}$), 44 (**1**$_{\mathbf{Sn}}$), 49 (**2**$_{\mathbf{Ge}}$) and 54(**2**$_{\mathbf{Sn}}$)). The NMR tubes were shaken to ensure homogeneity of the resultant solutions ca. 1 h before each NMR acquisitions. The data utilized to calculate the percentages of remaining HT and of the formed hydrolytic product tfppOH are given in Tables S1 and S2.

Reaction of tfppOH with aqueous HCl: HCl (37 % aqueous solution, 4.2 μL, 0.05 mmol) was added to an NMR tube containing a yellow solution of tfppOH (prepared by adding tfppOH (9.5 mg, 0.05 mmol) to *ca*. 0.3 mL of $CDCl_3$) and the resulting mixture was analyzed by ^{1}H NMR (Figure S56). The reaction led to the precipitation of a considerable amount of an orange solid.

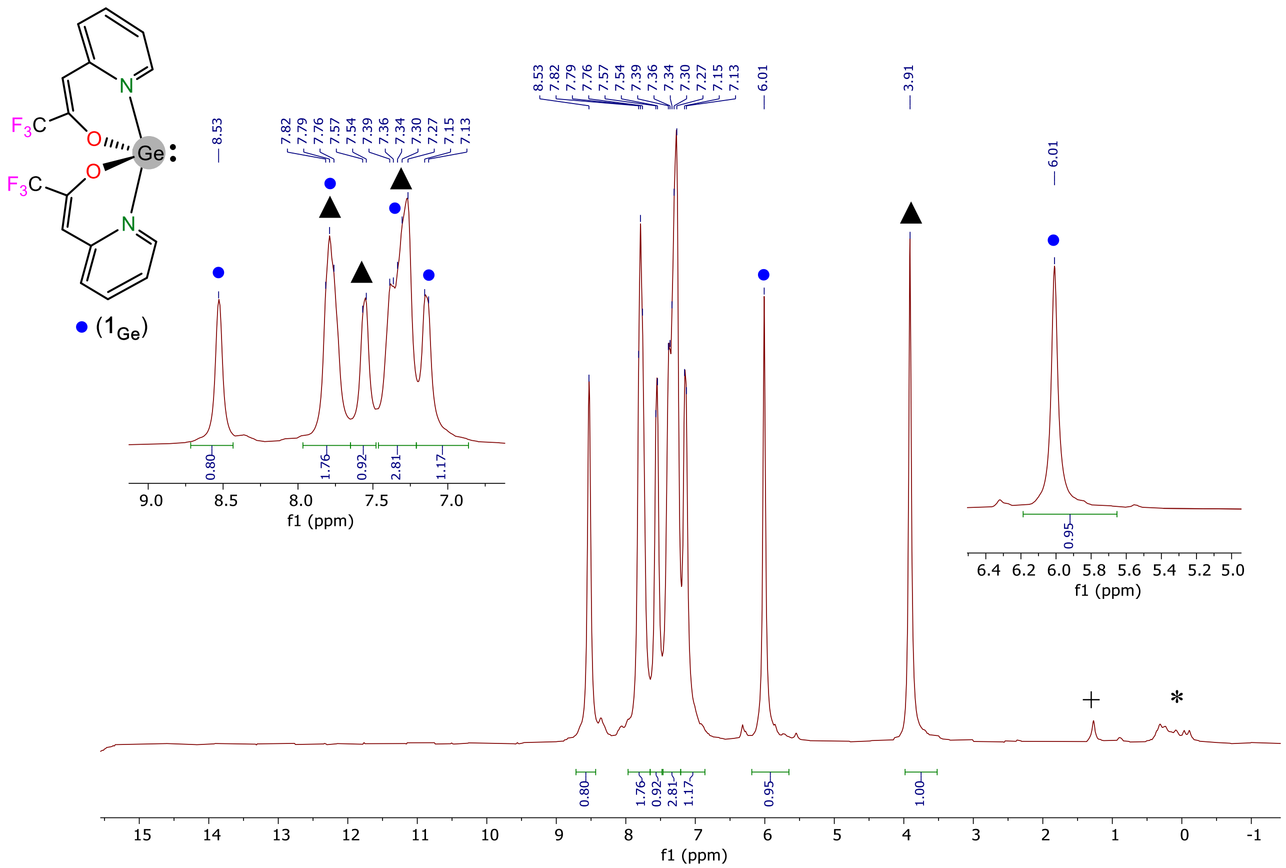

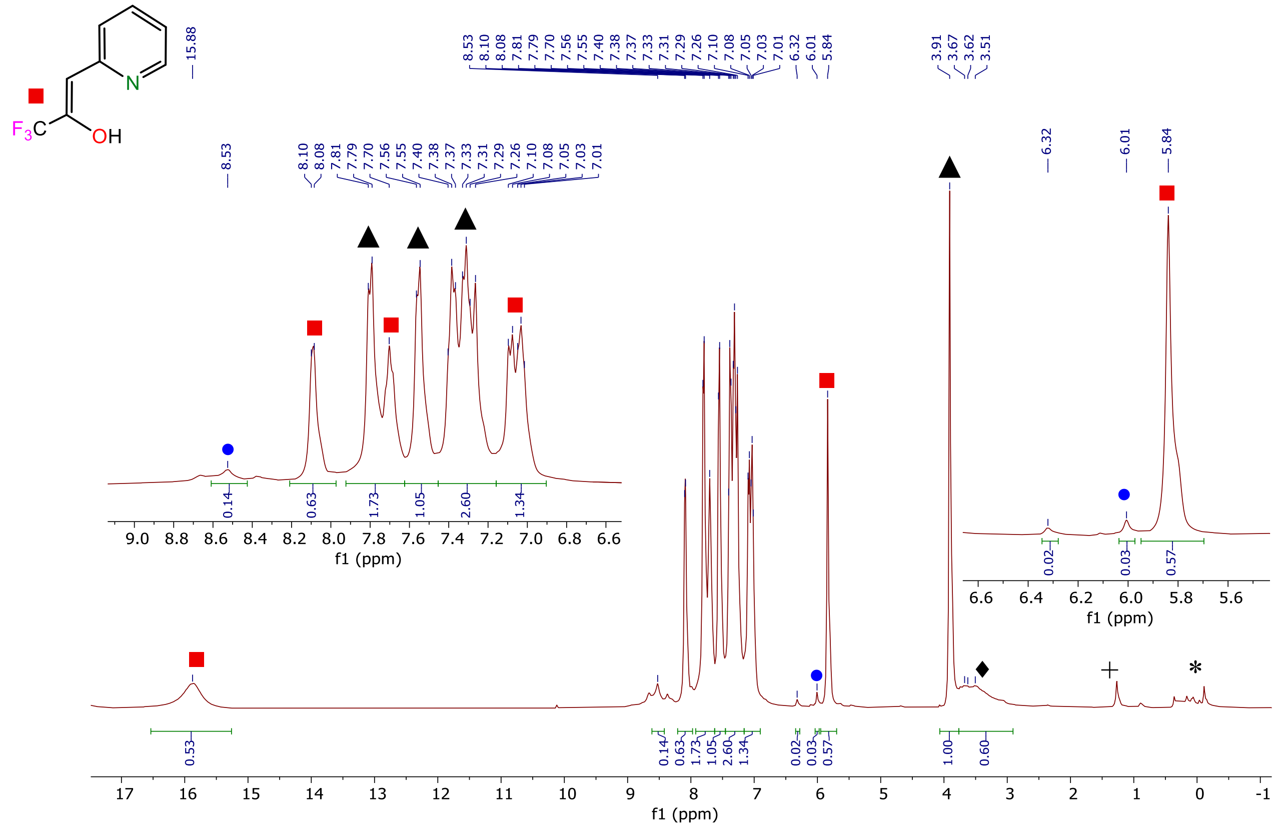

Figure S35. ^{1}H (300.1 MHz) NMR spectra ($CDCl_3$, 298 K) of **1Ge** and fluorene as standard under argon (top) and after standing in air for one day (bottom). (● = **1Ge**, ▲= fluorene, ■ = tfppOH, ♦ = unknown new species, + = paraffin grease, * = impurities).

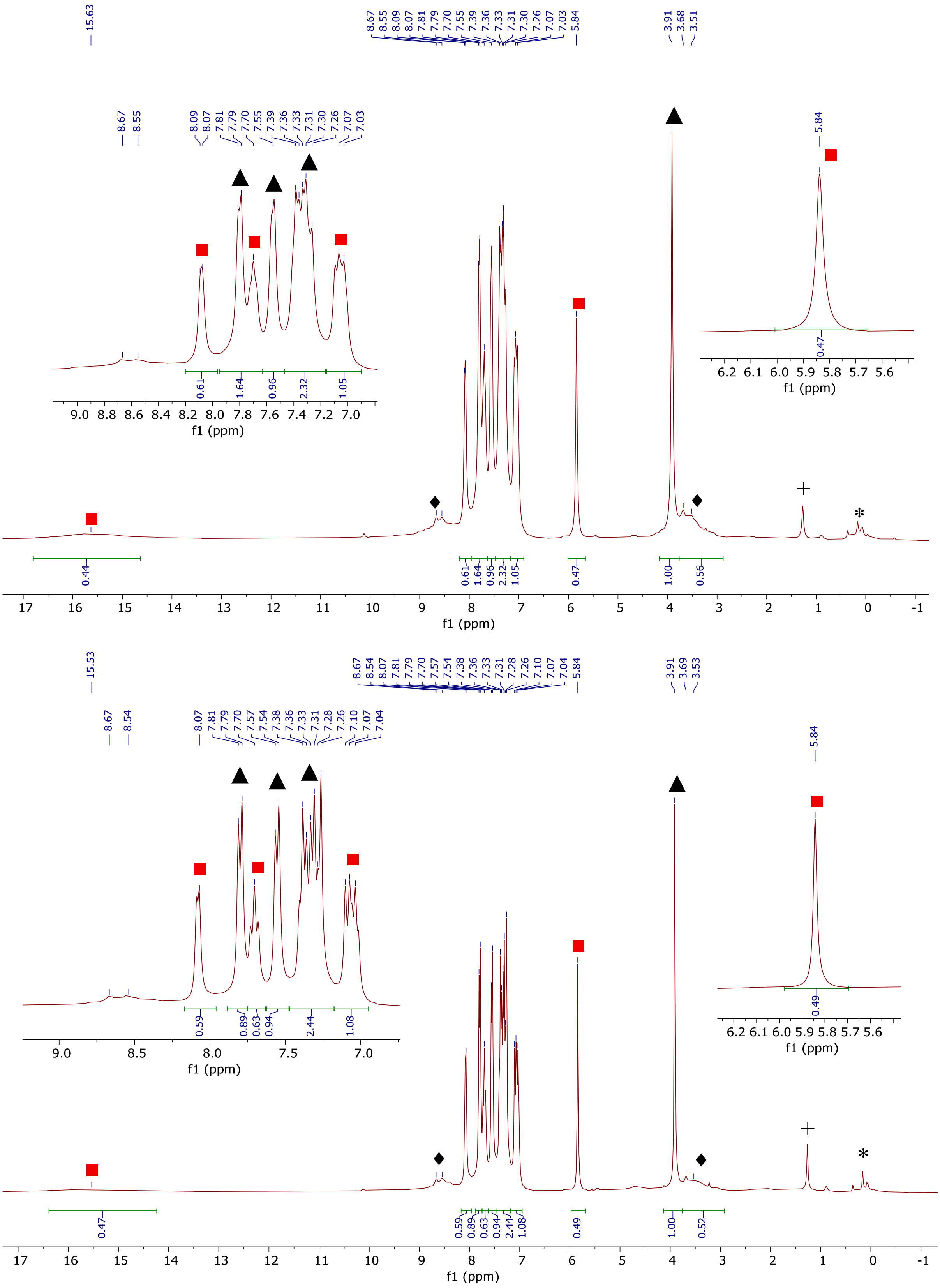

Figure S36. ^{1}H (300.1 MHz) NMR spectra ($CDCl_3$, 298 K) of **1**$_{Ge}$ and fluorene as standard after standing in air for three days (top) and seven days (bottom). (▲= fluorene, ■ = tfppOH, ♦ = unknown new species, + = paraffin grease, * = impurities).

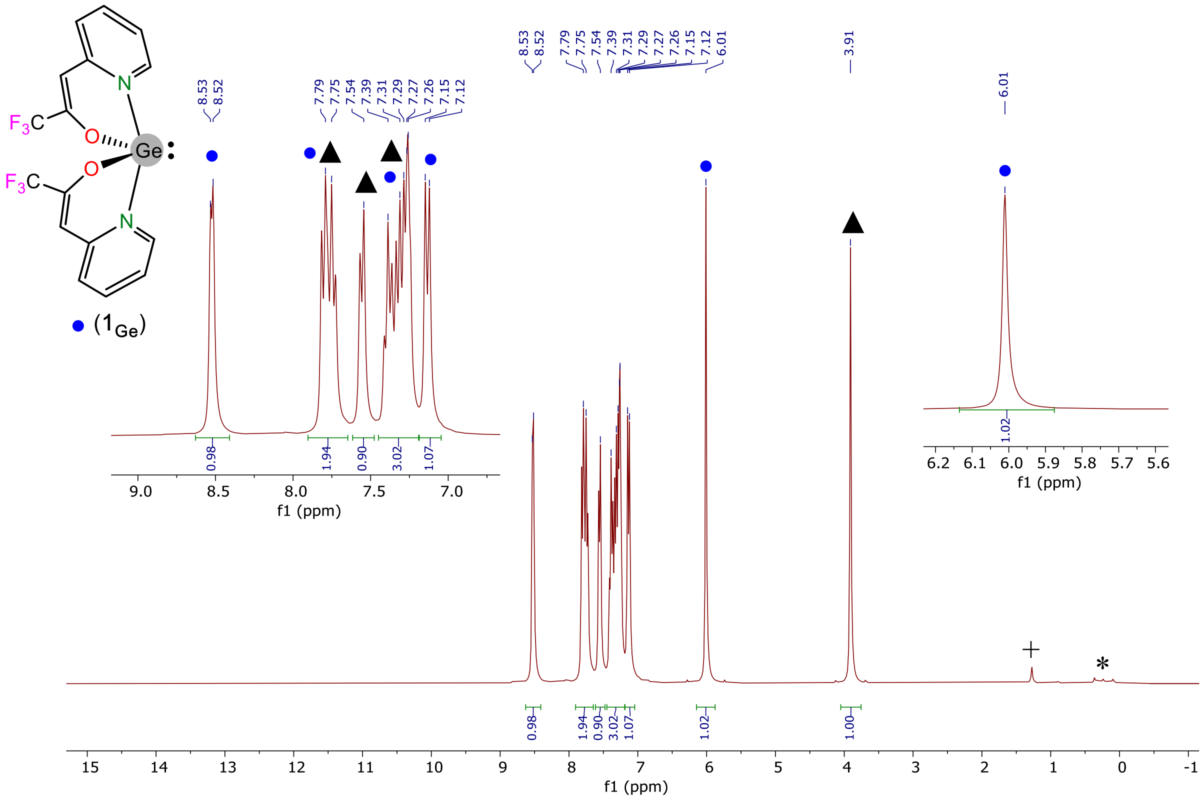

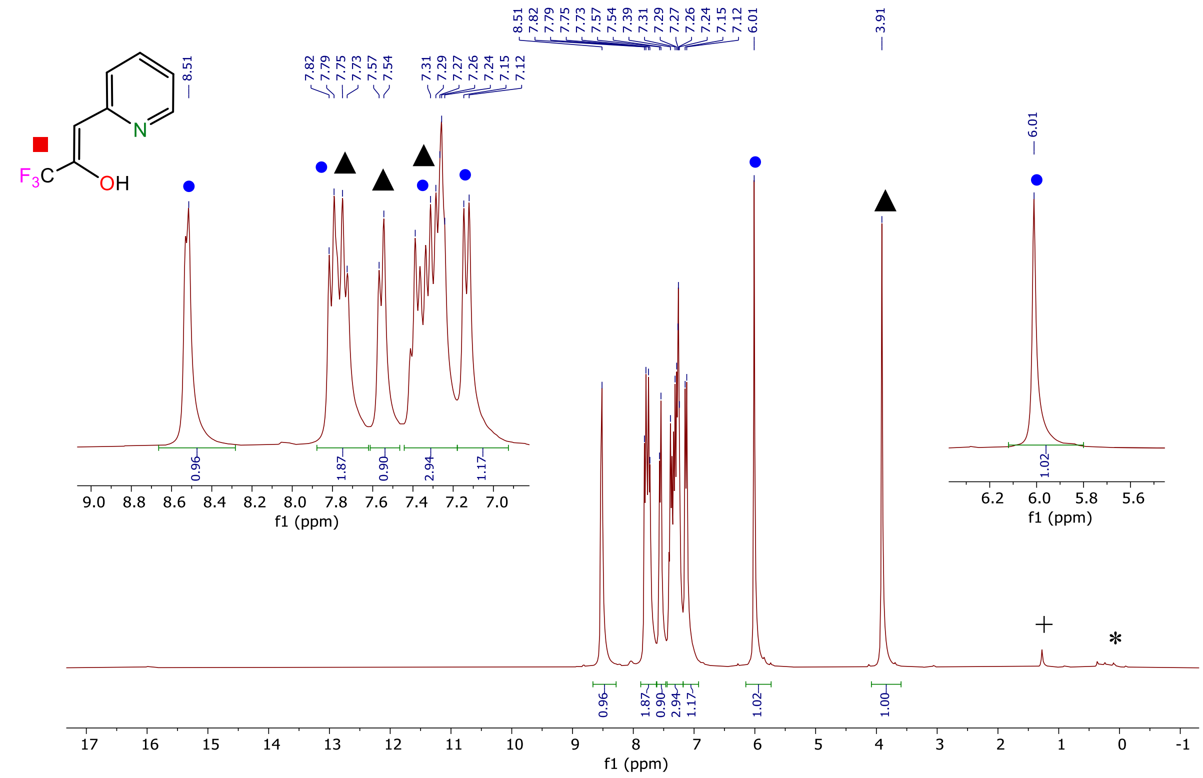

Figure S37. ^{1}H (300.1 MHz) NMR spectra ($CDCl_3$, 298 K) of **1Ge** and fluorene as standard under argon (top) and after standing in dry air for one day (bottom). (● = **1Ge**, ▲ = fluorene, + = paraffin grease, * = impurities).

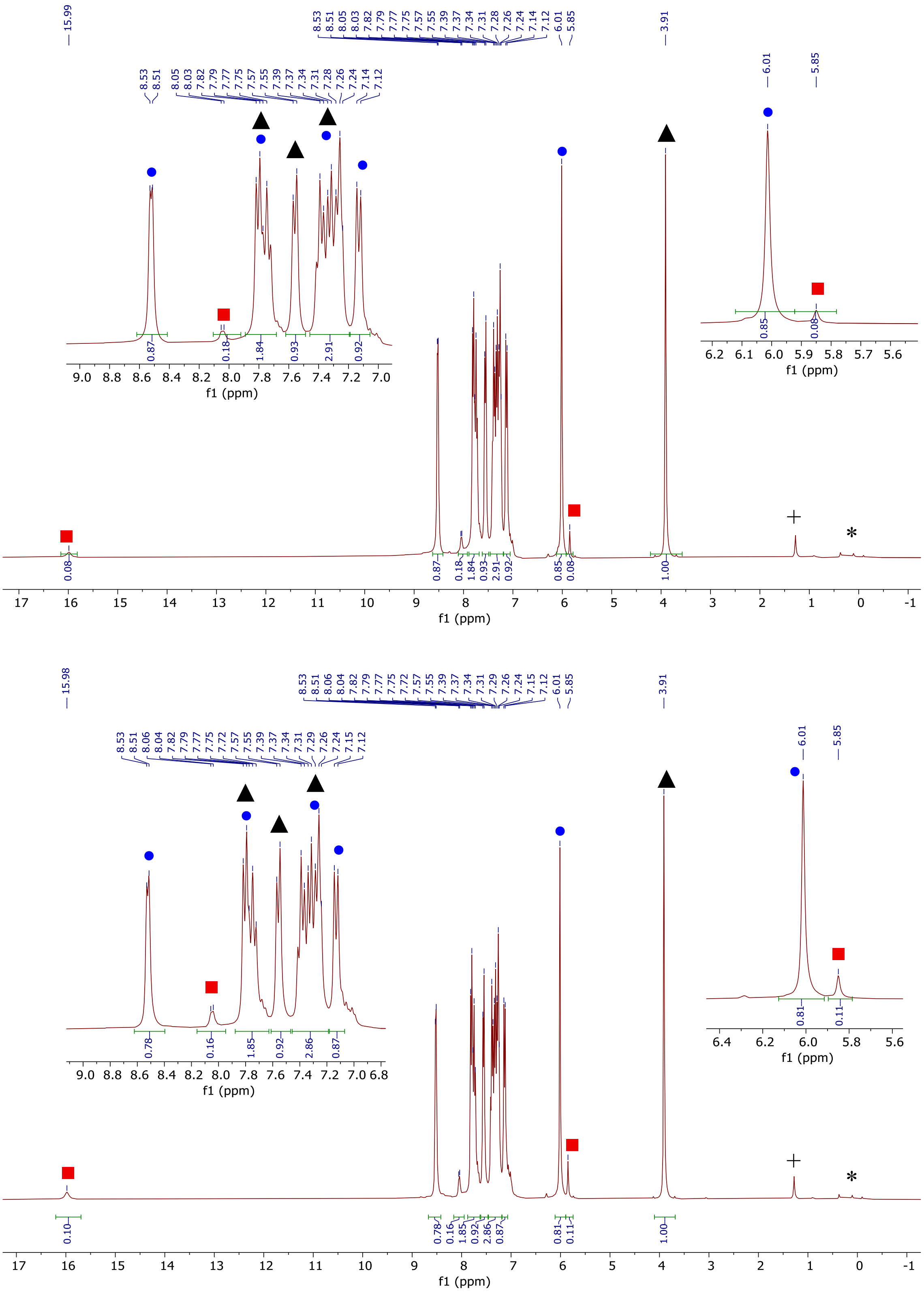

Figure S38. ^{1}H (300.1 MHz) NMR spectra ($CDCl_3$, 298 K) of $\mathbf{1}_{\mathbf{Ge}}$ and fluorene as standard after standing in dry air for four days (top) and seven days (bottom). (● = $\mathbf{1}_{\mathbf{Ge}}$, ▲ = fluorene, ■ = tfppOH, + = paraffin grease, * = impurities).

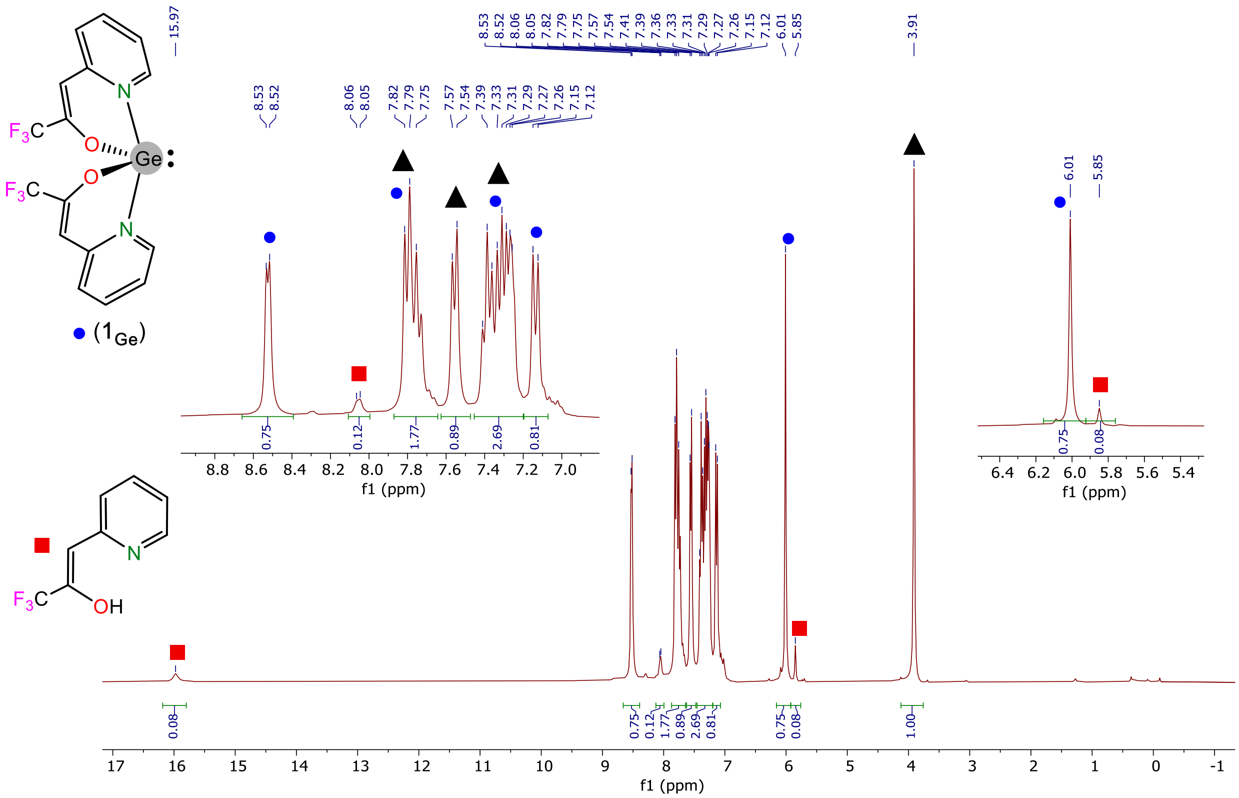

Figure S39. ^{1}H (300.1 MHz) NMR spectrum ($CDCl_3$, 298 K) of $\mathbf{1_{Ge}}$ and fluorene as standard after standing in air for one day as a solid. (● = $\mathbf{1_{Ge}}$, ▲= fluorene, ■ = tfppOH).

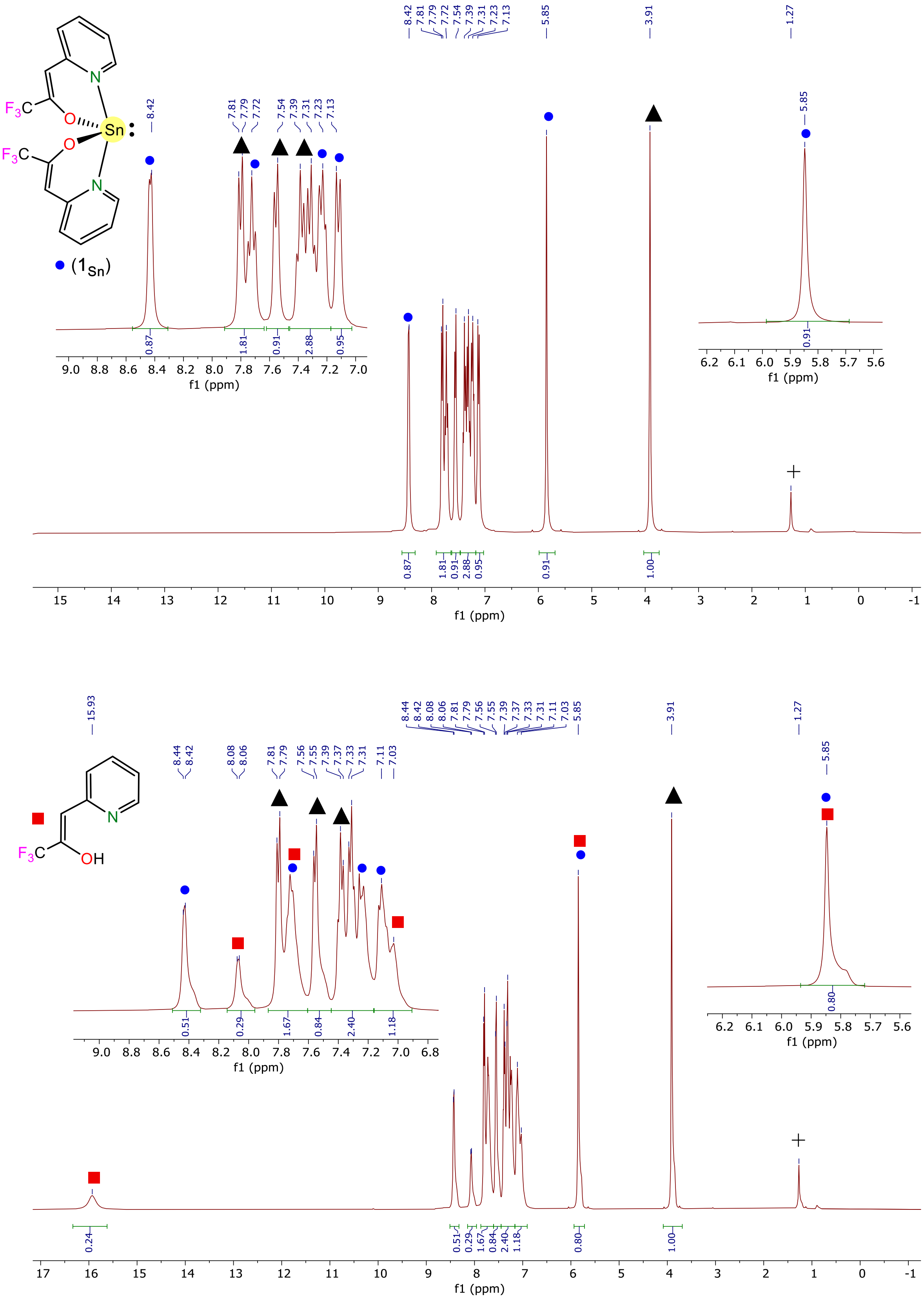

Figure S40. ^{1}H (300.1 MHz) NMR spectra ($CDCl_3$, 298 K) of **1**$_{\mathbf{Sn}}$ and fluorene as standard under argon (top) and after standing in air for one day (bottom). (● = **1**$_{\mathbf{Sn}}$, ▲= fluorene, ■ = tfppOH, + = paraffin grease).

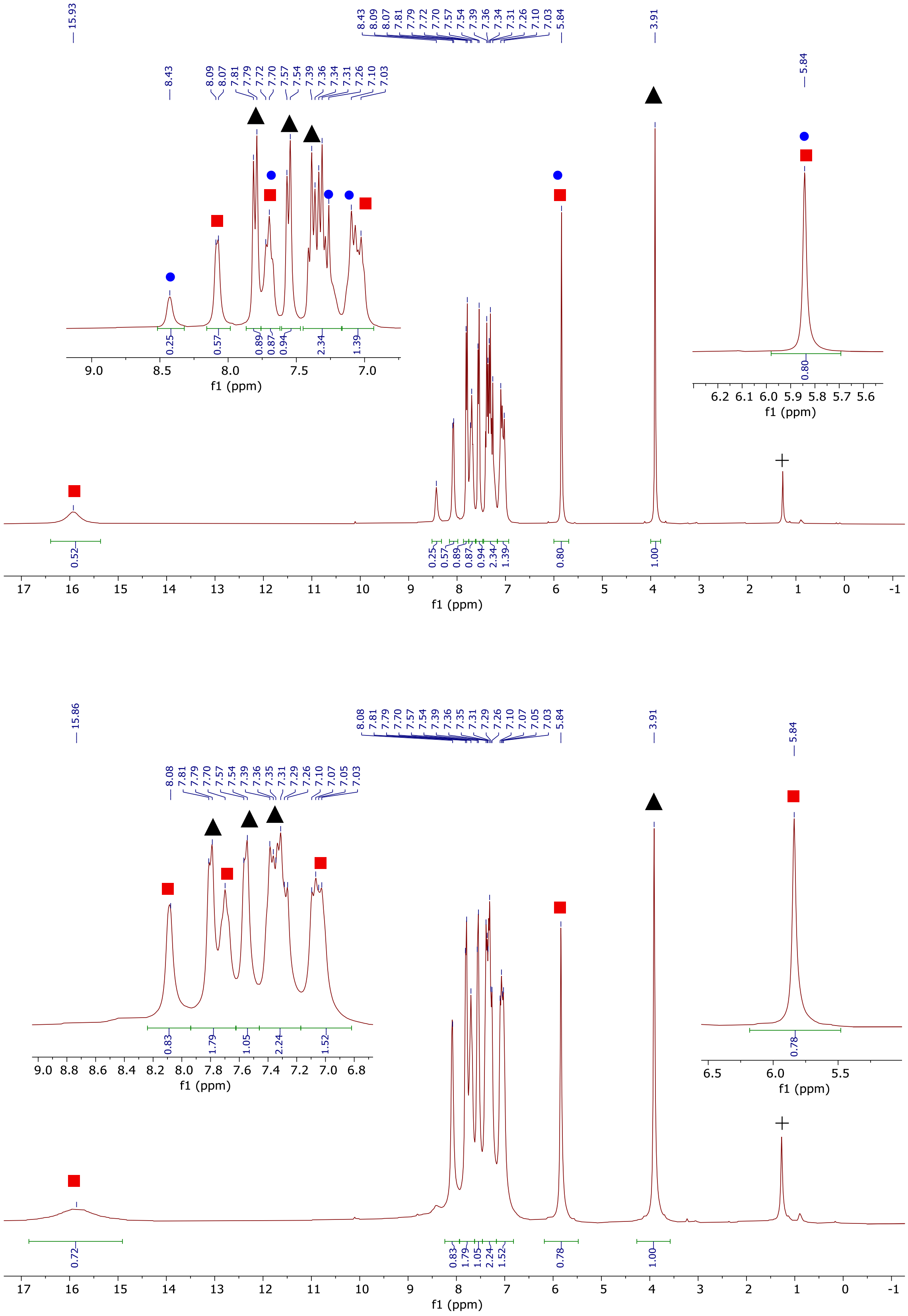

Figure S41. ^{1}H (300.1 MHz) NMR spectra ($CDCl_3$, 298 K) of $\mathbf{1_{Sn}}$ and fluorene as standard after standing in air for three days (top) and seven days (bottom). (● = $\mathbf{1_{Sn}}$, ▲= fluorene, ■ = tfppOH, + = paraffin grease).

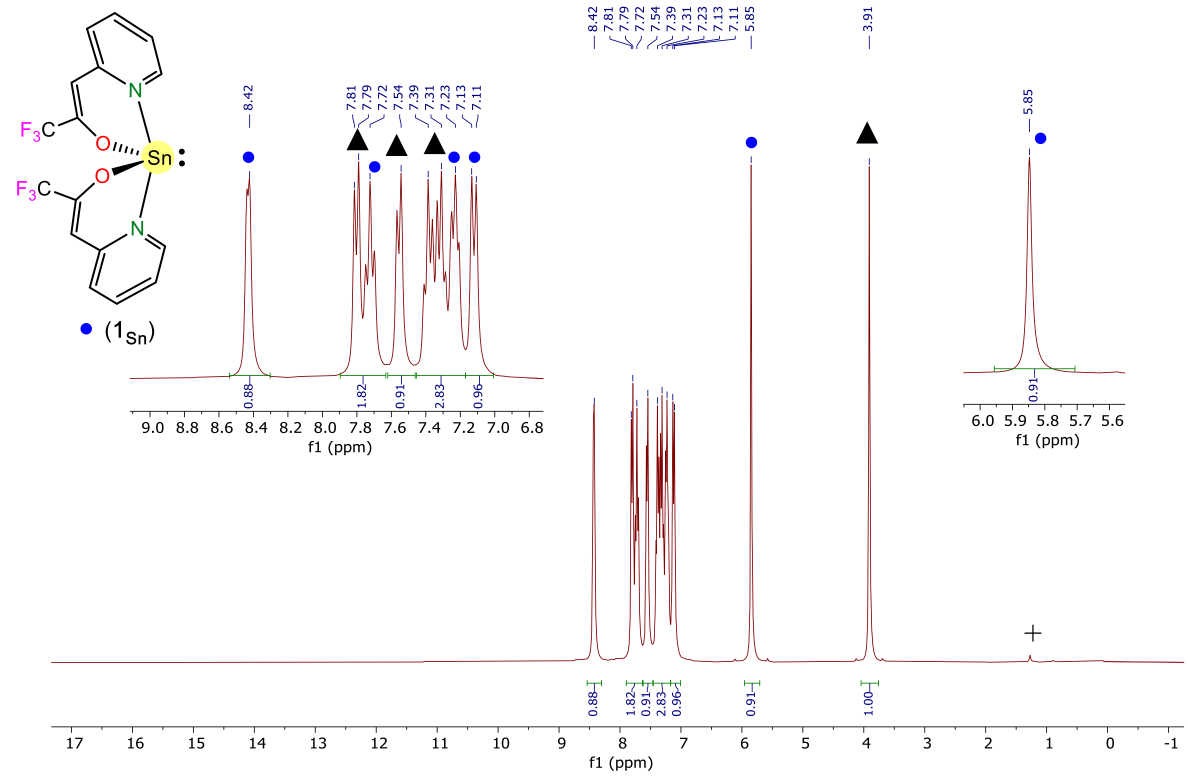

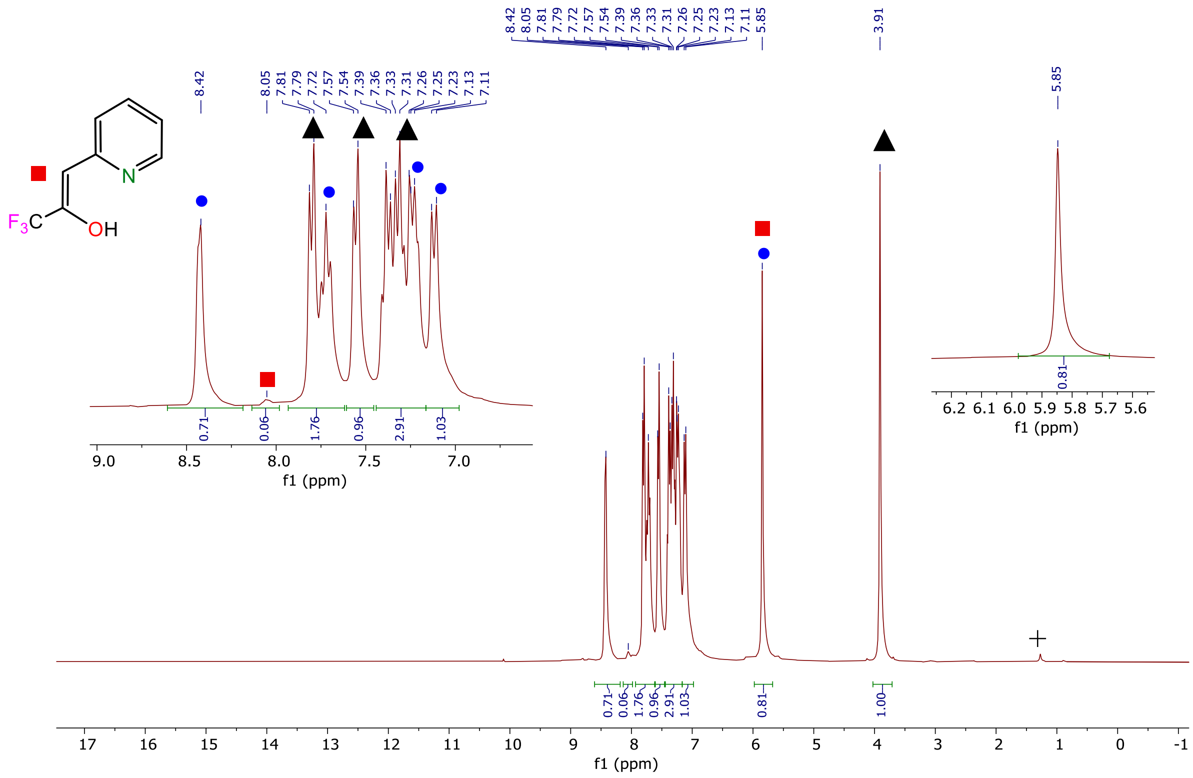

Figure S42. ^{1}H (300.1 MHz) NMR spectra ($CDCl_3$, 298 K) of $\mathbf{1_{Sn}}$ and fluorene as standard under argon (top) and after standing in dry air for one day (bottom). (● = $\mathbf{1_{Sn}}$, ▲= fluorene, ■ = tfppOH, + = paraffin grease).

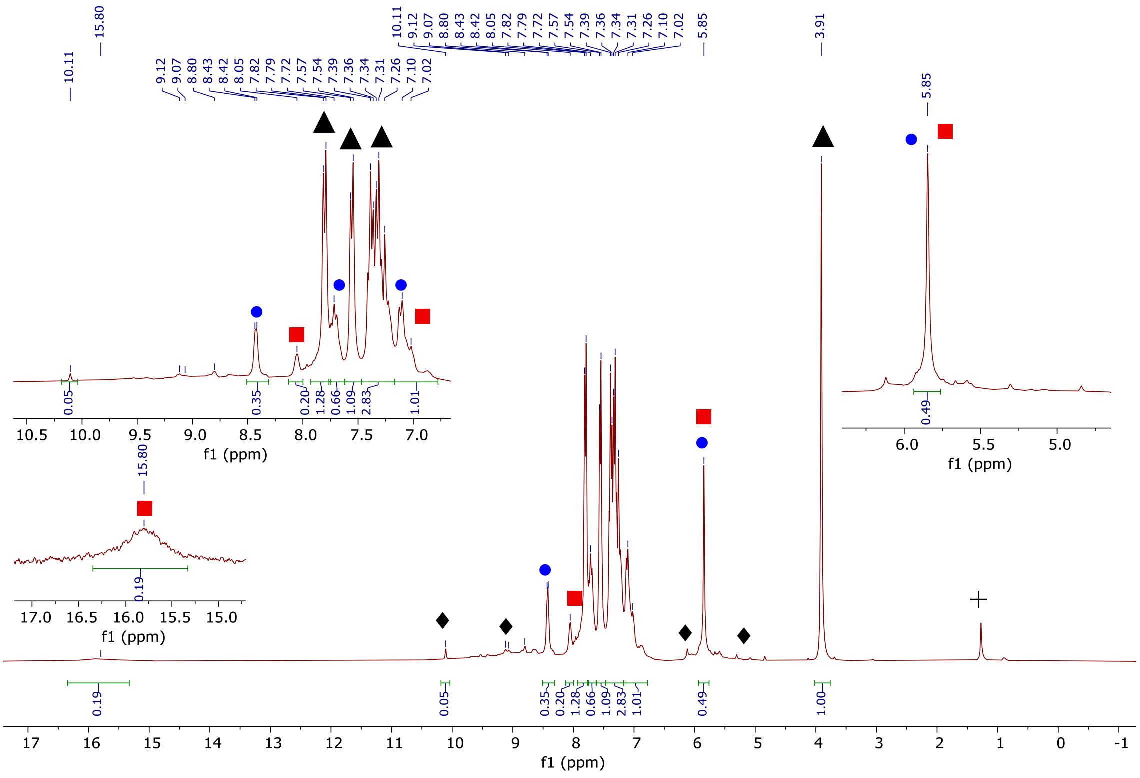

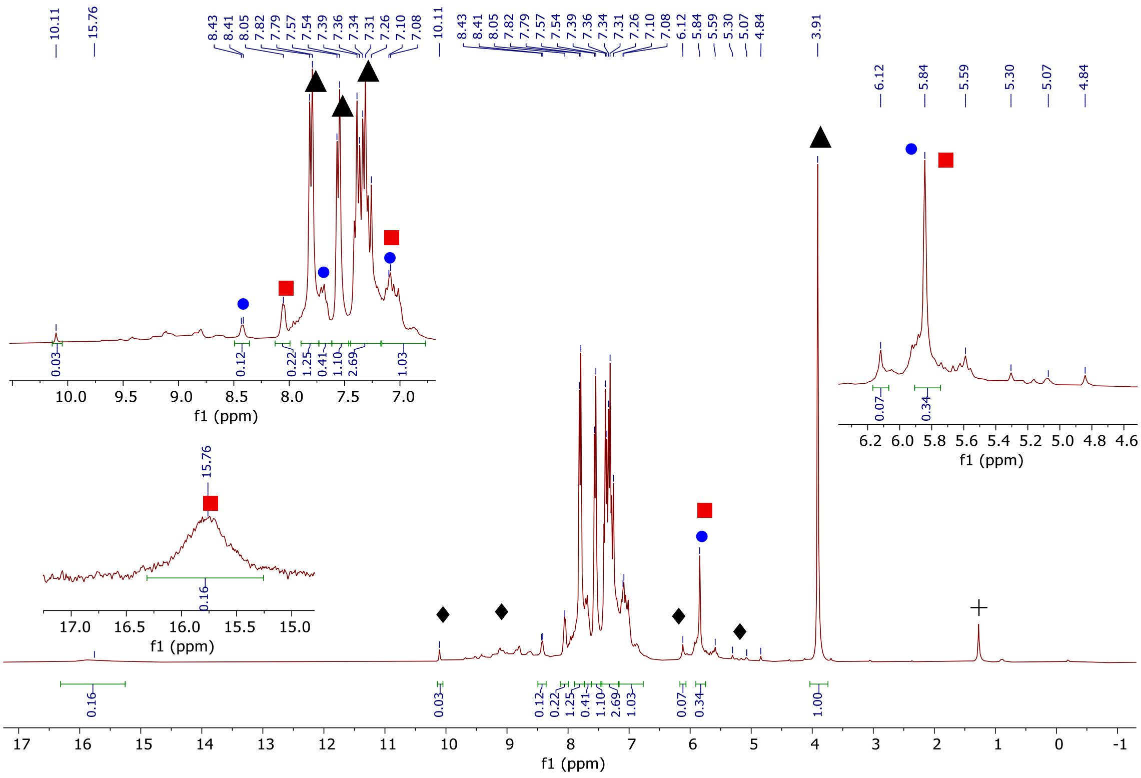

Figure S43. ^{1}H (300.1 MHz) NMR spectra ($CDCl_3$, 298 K) of $\mathbf{1_{Sn}}$ and fluorene as standard after standing in dry air for four days (top) and seven days (bottom). (● = $\mathbf{1_{Sn}}$, ▲= fluorene, ■ = tfppOH, ♦ = unknown new species, + = paraffin grease).

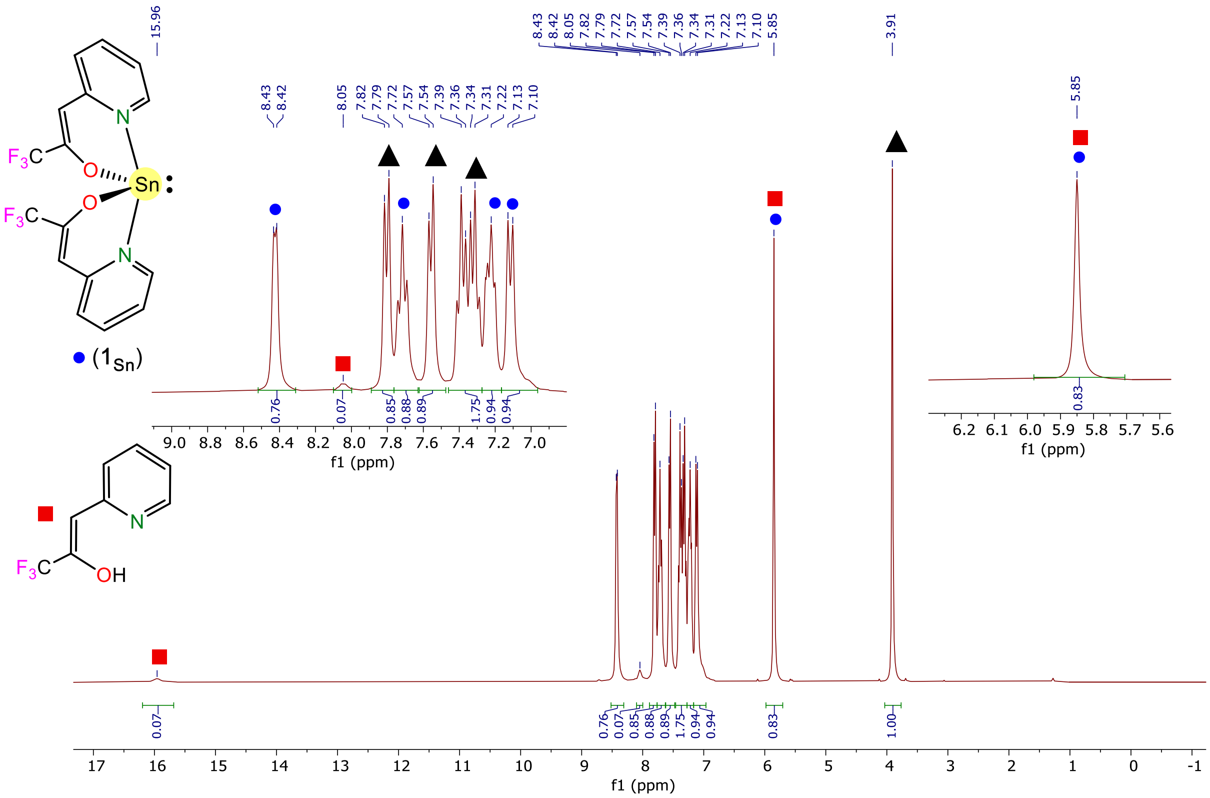

Figure S44. ^{1}H (300.1 MHz) NMR spectrum ($CDCl_3$, 298 K) of **1$_{Sn}$** and fluorene as standard after standing in air for one day as a solid. (● = **1$_{Sn}$**, ▲= fluorene, ■ = tfppOH).

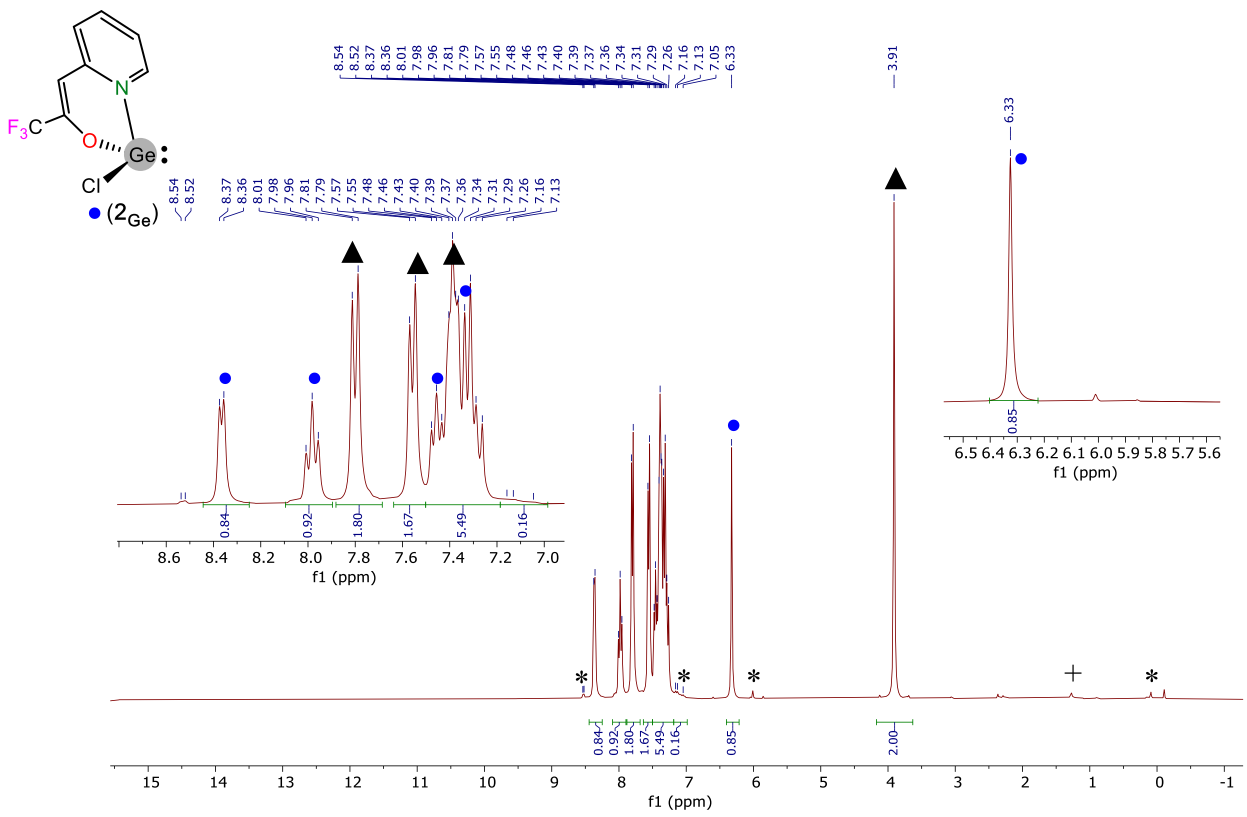

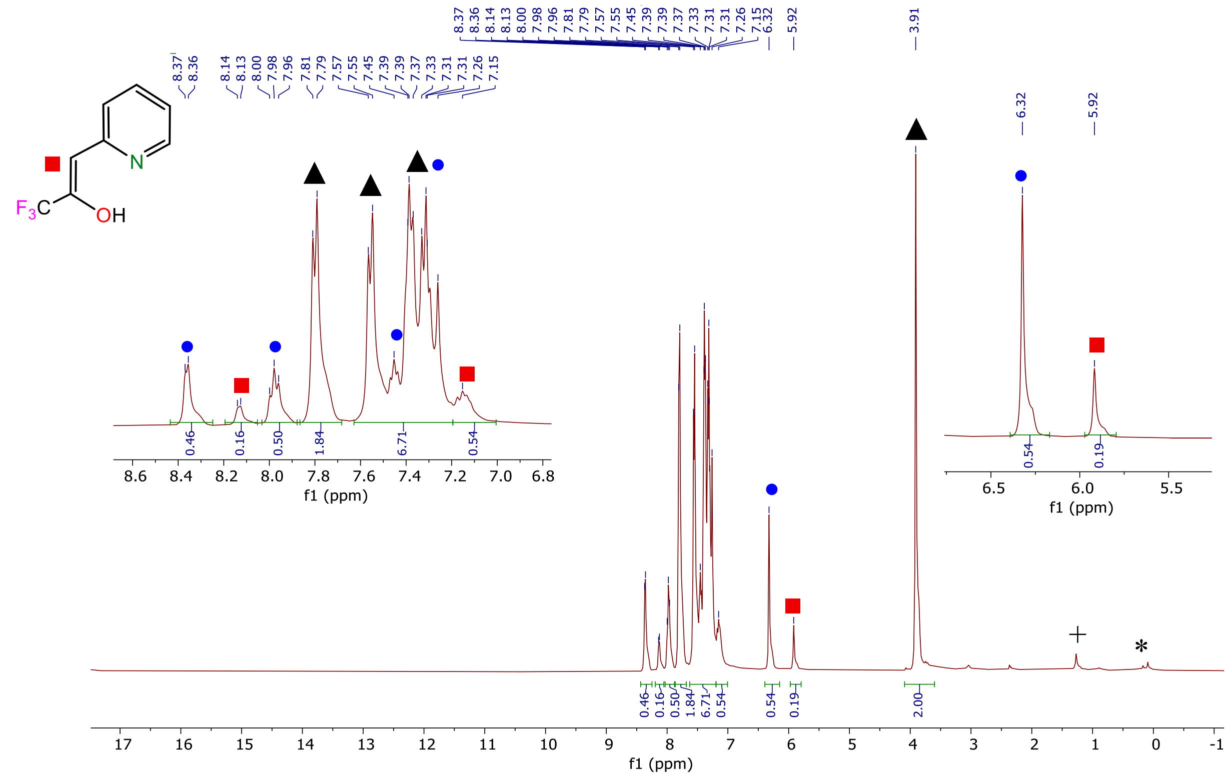

Figure S45. ^{1}H (300.1 MHz) NMR spectra ($CDCl_3$, 298 K) of **2$_{Ge}$** and fluorene as standard under argon (top) and after standing in air for one day (bottom). (● = **2$_{Ge}$**, ▲= fluorene, ■ = tfppOH, + paraffin grease, * = impurities).

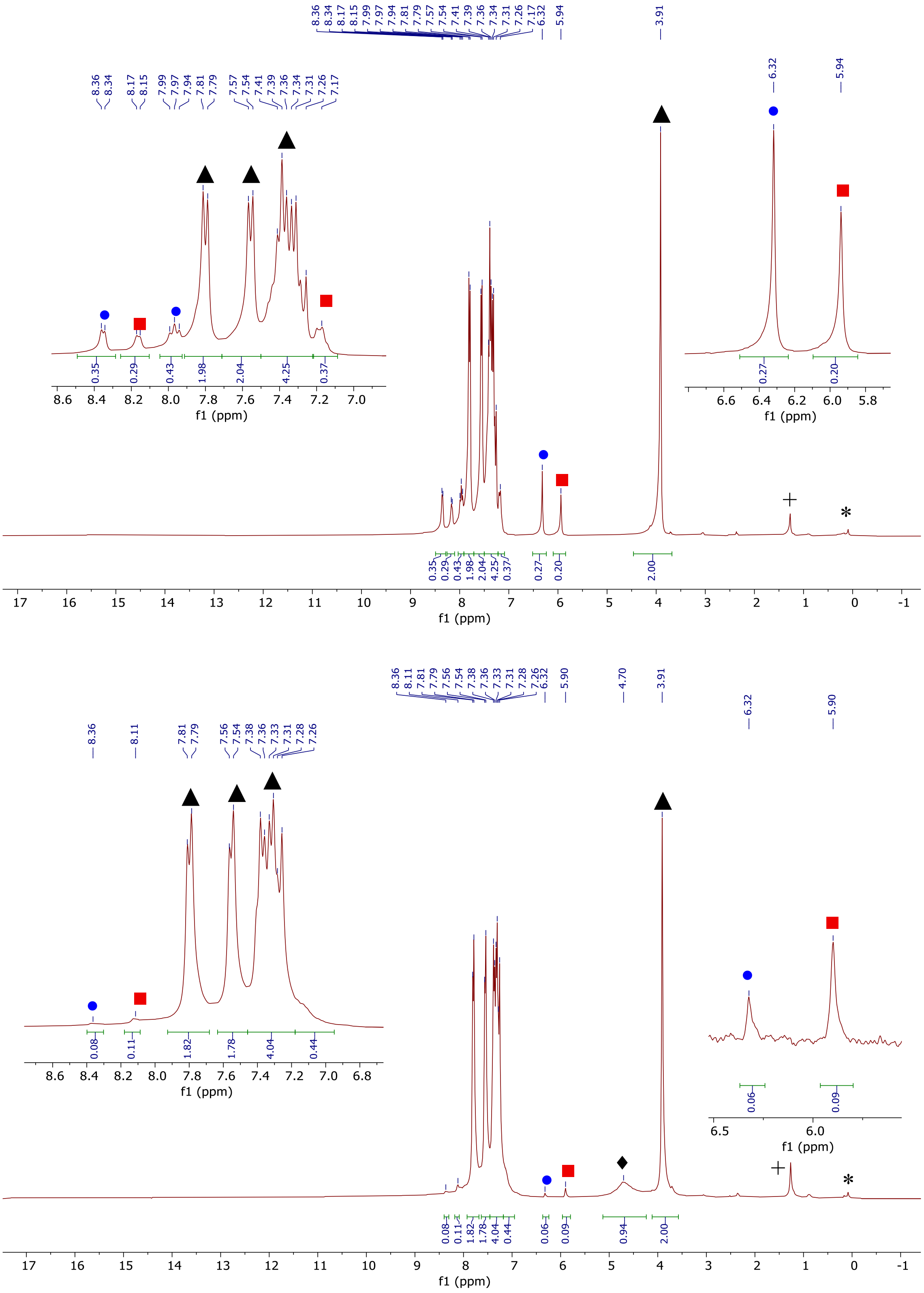

Figure S46. ^{1}H (300.1 MHz) NMR spectra ($CDCl_3$, 298 K) of $\mathbf{2}_{\mathbf{Ge}}$ and fluorene as standard after standing in air for three days (top) and seven days (bottom). (● = $\mathbf{2}_{\mathbf{Ge}}$, ▲= fluorene, ■ = tfppOH, ♦ = unknown new species, + paraffin grease, * = impurities).

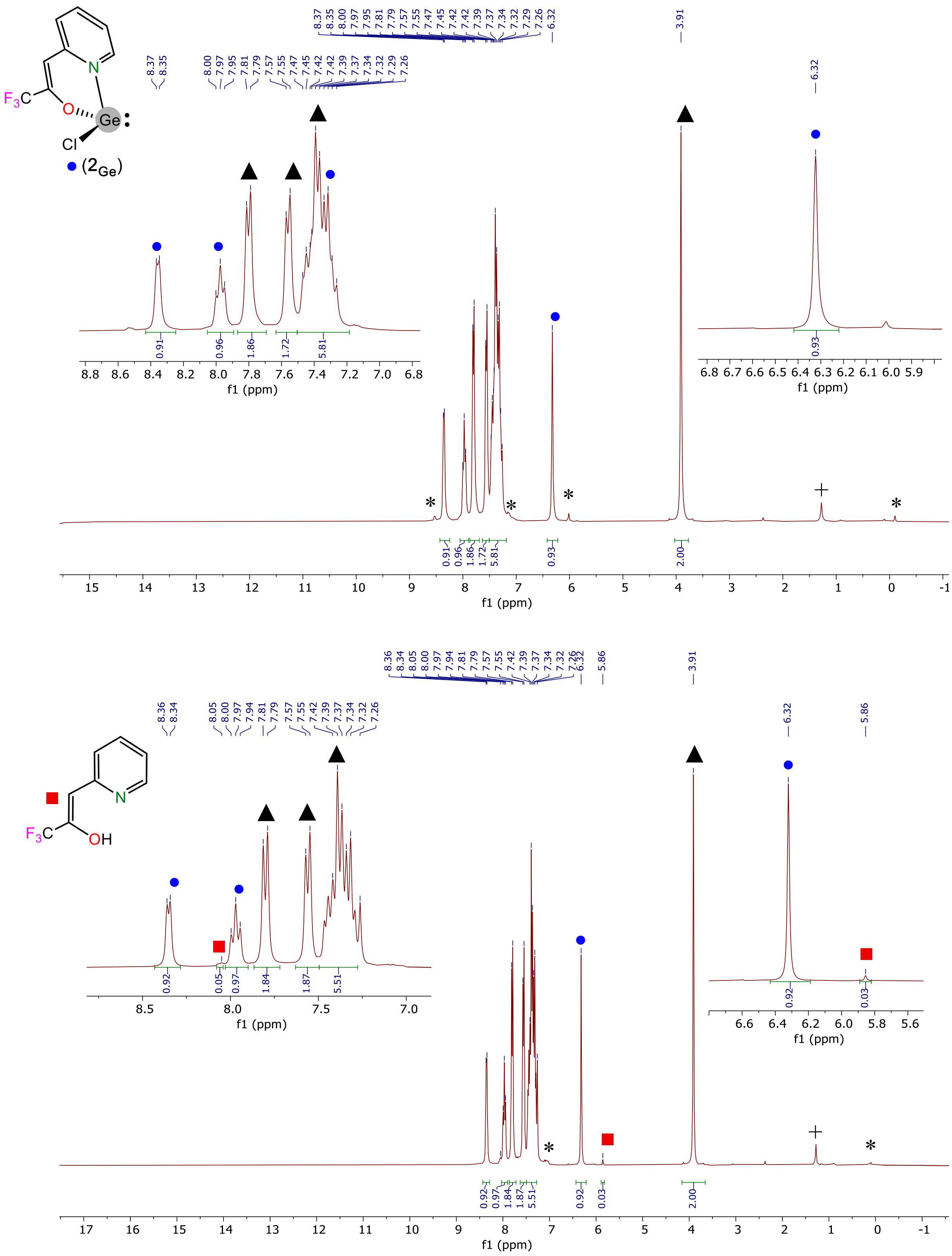

Figure S47. ^{1}H (300.1 MHz) NMR spectra ($CDCl_3$, 298 K) of **2Ge** and fluorene as standard under argon (top) and after standing in dry air for one day (bottom). (● = **2Ge**, ▲= fluorene, ■ = tfppOH, + paraffin grease, * = impurities).

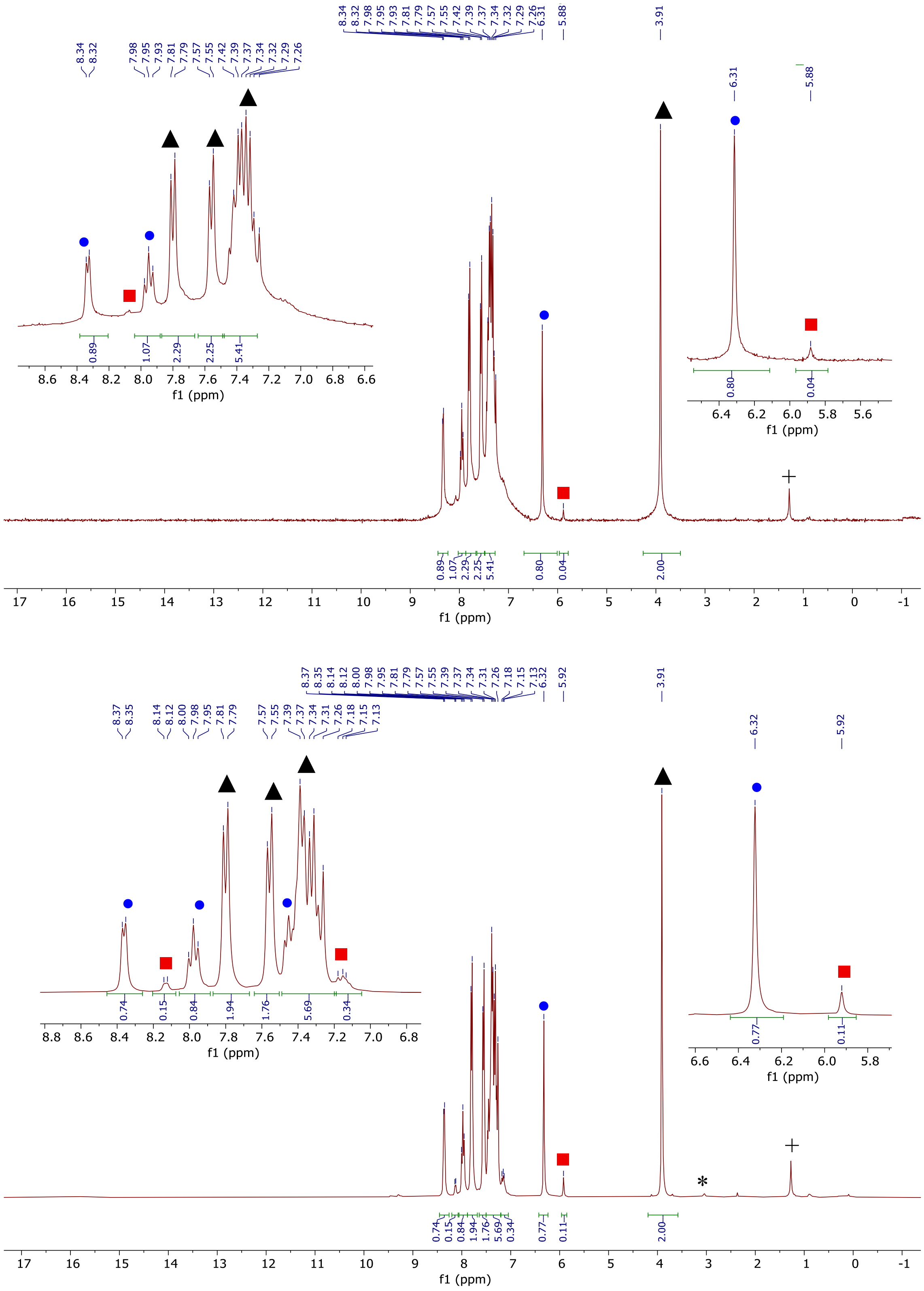

Figure S48. ^{1}H (300.1 MHz) NMR spectra ($CDCl_3$, 298 K) of **2**$_{\mathbf{Ge}}$ and fluorene as standard after standing in dry air for four days (top) and seen days (bottom). (● = **2**$_{\mathbf{Ge}}$, ▲= fluorene, ■ = tfppOH, + paraffin grease, * = impurities).

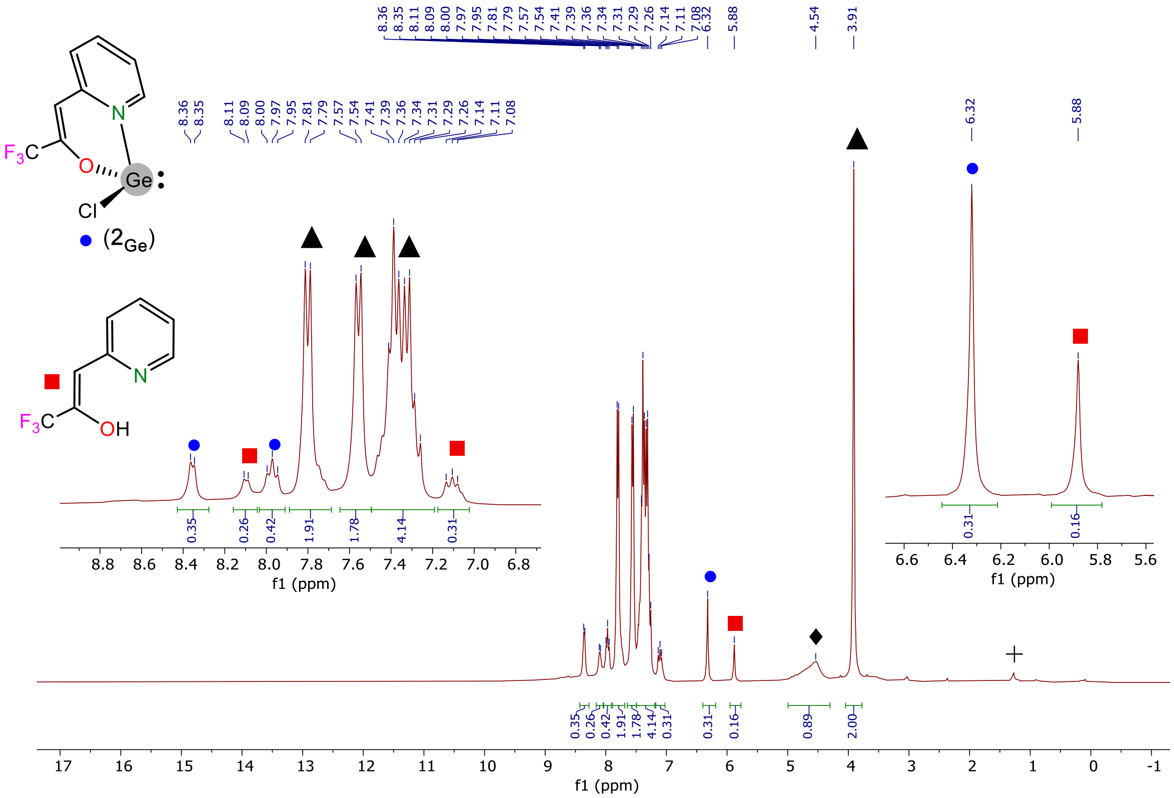

Figure S49. ^{1}H (300.1 MHz) NMR spectrum ($CDCl_3$, 298 K) of $\mathbf{2_{Ge}}$ and fluorene as standard after standing in air for one day as a solid. (● = $\mathbf{2_{Ge}}$, ▲= fluorene, ■ = tfppOH, ♦ = unknown new species, + paraffin grease).

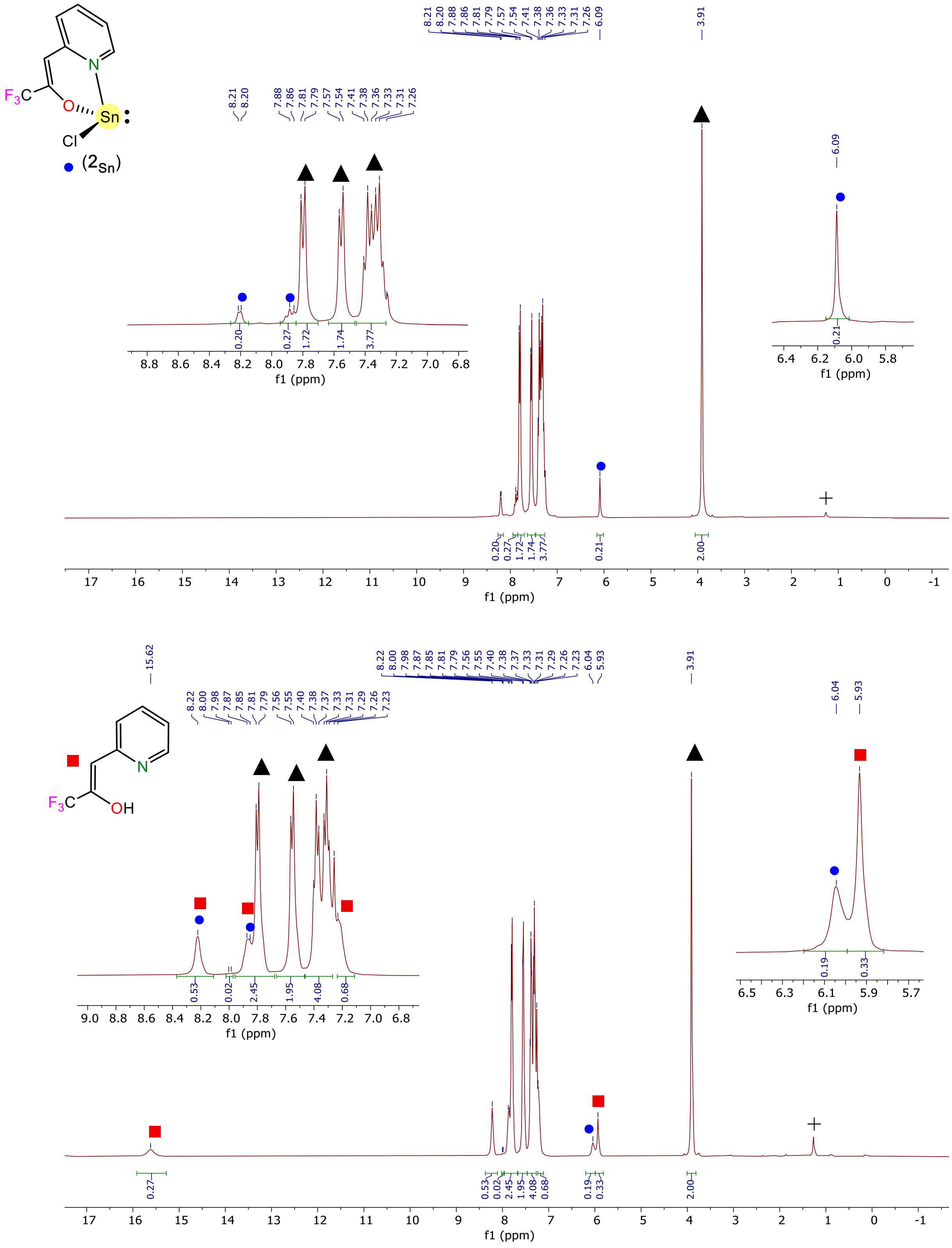

Figure S50. ^{1}H (300.1 MHz) NMR spectra ($CDCl_3$, 298 K) of **2Sn** and fluorene as standard under argon (top) and after standing in air for one day (bottom). (● = **2Sn**, ▲= fluorene, ■ = tfppOH, + = paraffin grease).

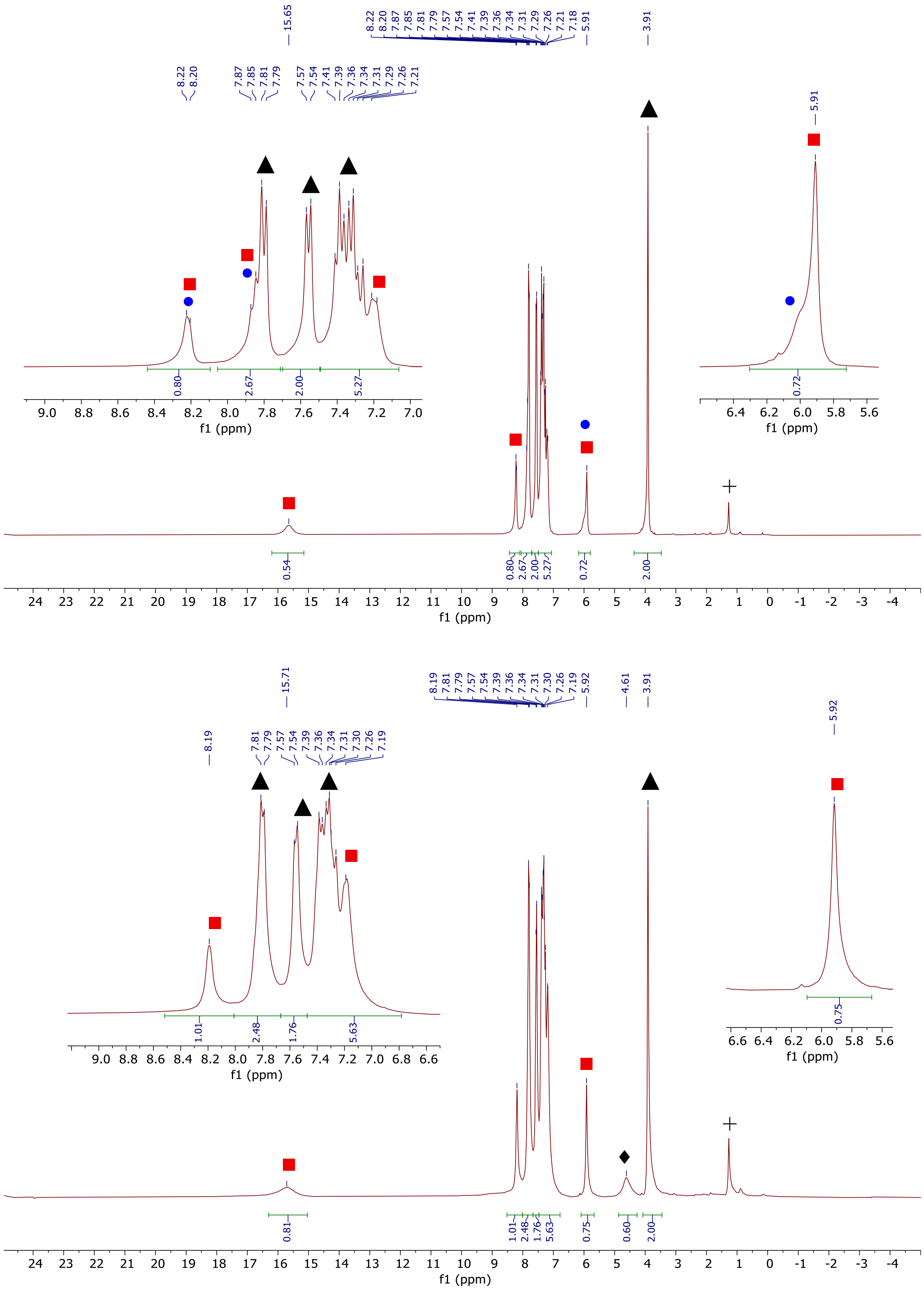

Figure S51. ^{1}H (300.1 MHz) NMR spectra ($CDCl_3$, 298 K) of $\mathbf{2_{Sn}}$ and fluorene as standard after standing in air for three days (top) and seven days (bottom). (● = $\mathbf{2_{Sn}}$, ▲= fluorene, ■ = tfppOH, ♦ = unknown new species, + paraffin grease).

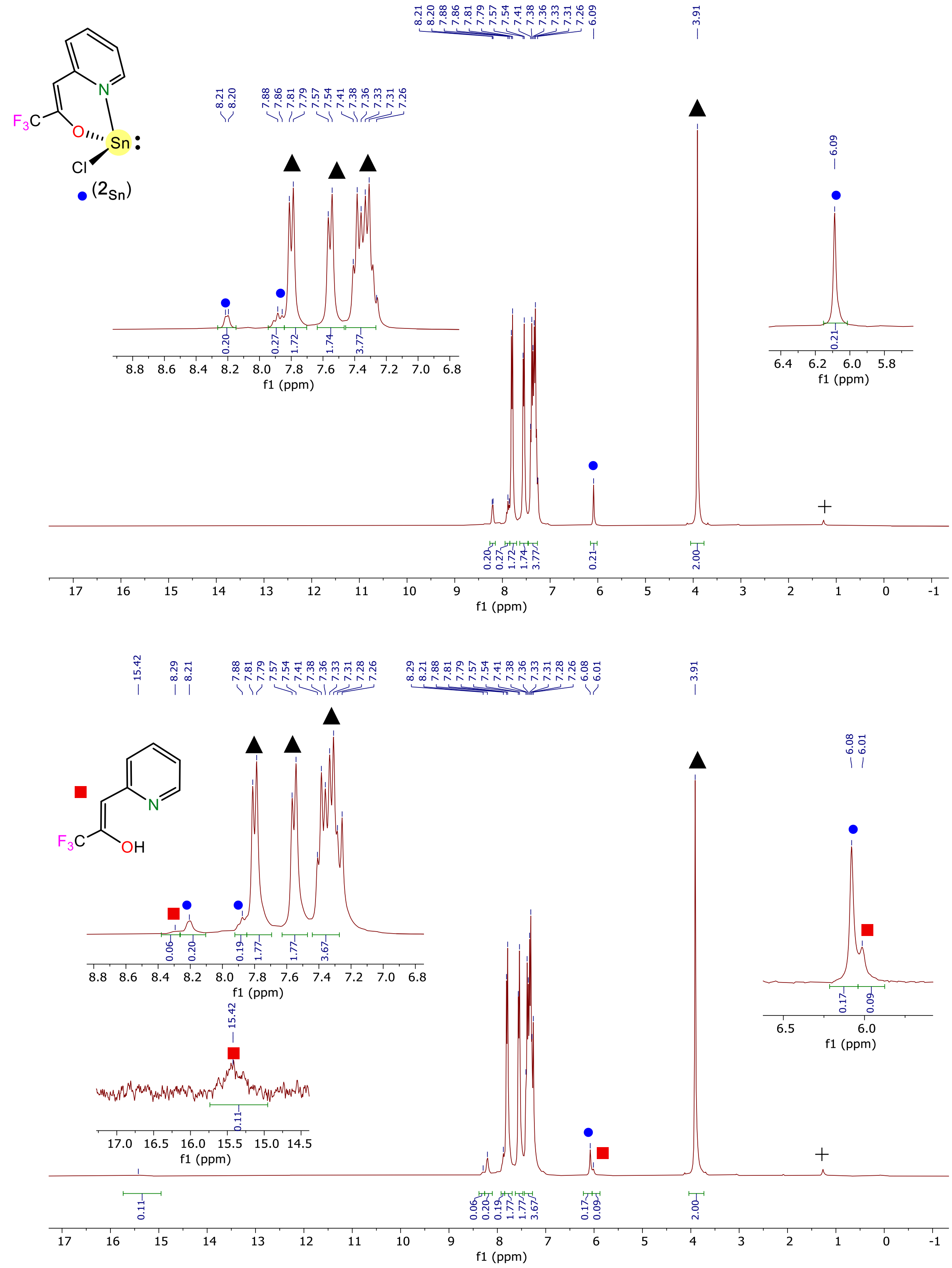

Figure S52. ^{1}H (300.1 MHz) NMR spectra ($CDCl_3$, 298 K) of $\mathbf{2_{Sn}}$ and fluorene as standard under argon (top) and after standing in dry air for one day (bottom). (● = $\mathbf{2_{Sn}}$, ▲= fluorene, ■ = tfppOH, + = paraffin grease).

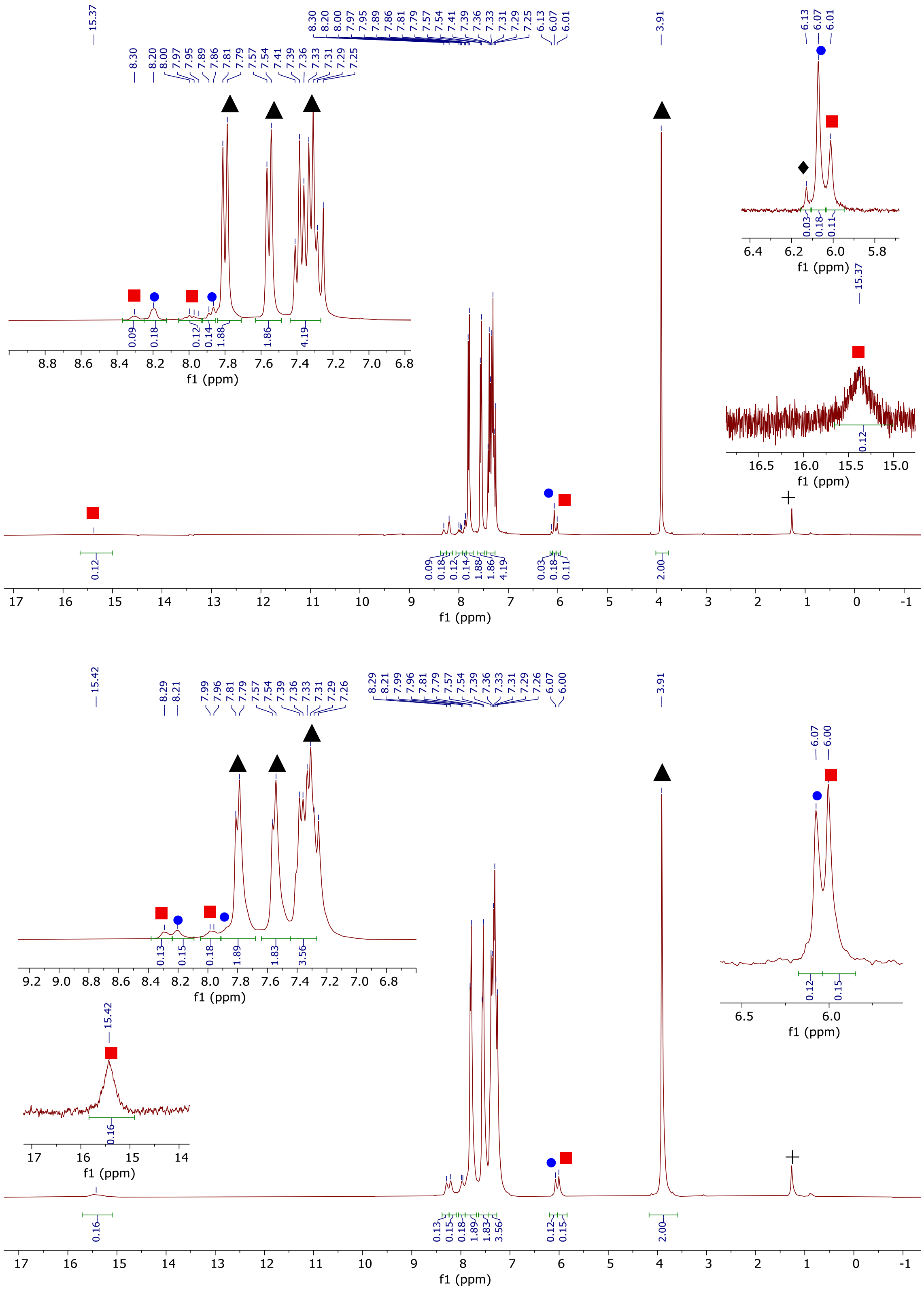

Figure S53. ^{1}H (300.1 MHz) NMR spectra ($CDCl_3$, 298 K) of **2$_{Sn}$** and fluorene as standard after standing in dry air for four days (top) and seven days (bottom). (● = **2$_{Sn}$**, ▲= fluorene, ■ = tfppOH, ♦ = unknown new species, + paraffin grease).

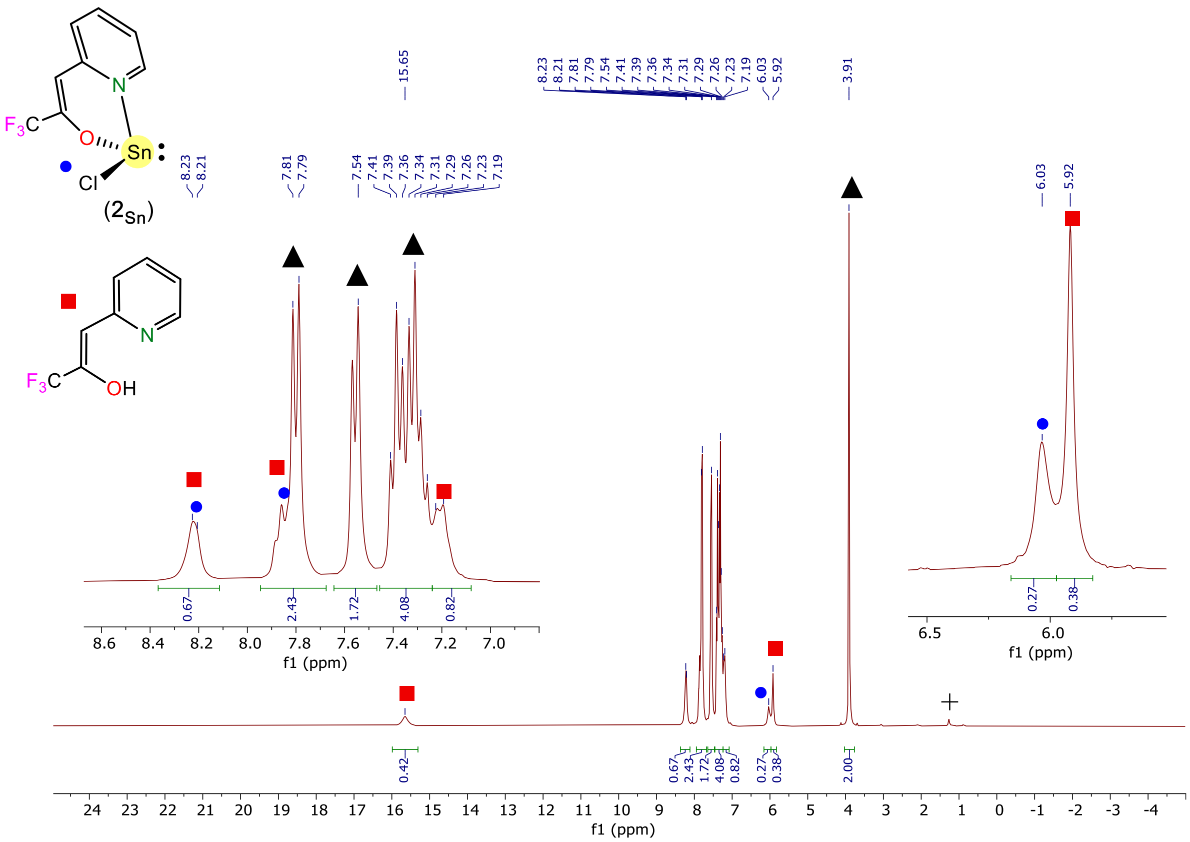

Figure S54. ^{1}H (300.1 MHz) NMR spectrum ($CDCl_3$, 298 K) of **2_{Sn}** and fluorene as standard after standing in air for one day as a solid. (● = **2_{Sn}**, ▲= fluorene, ■ = tfppOH, + = paraffin grease).

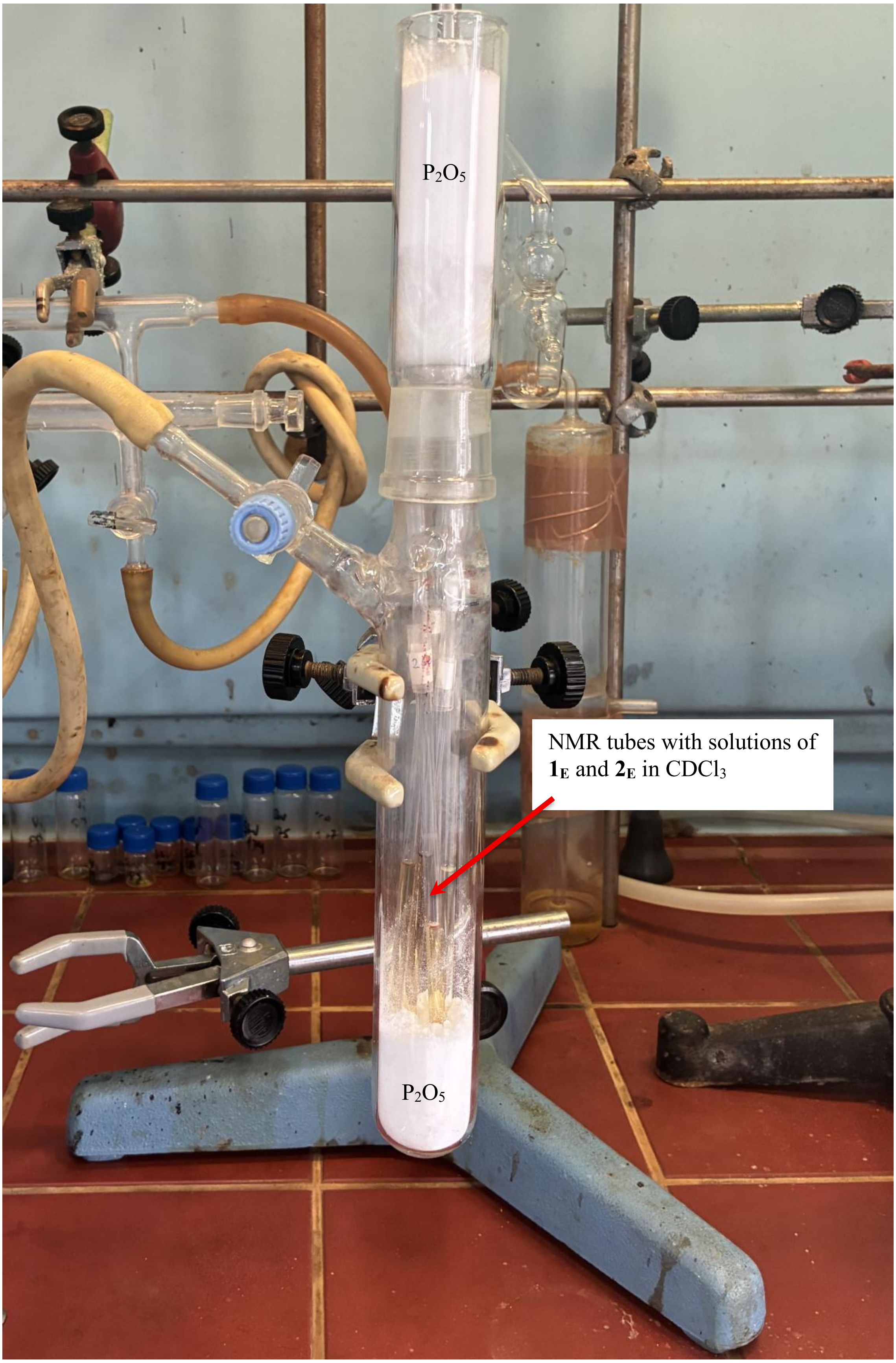

Figure S55. Schlenk flask used to study the dry air-stability of **1E** and **2E** in $CDCl_3$ solution.

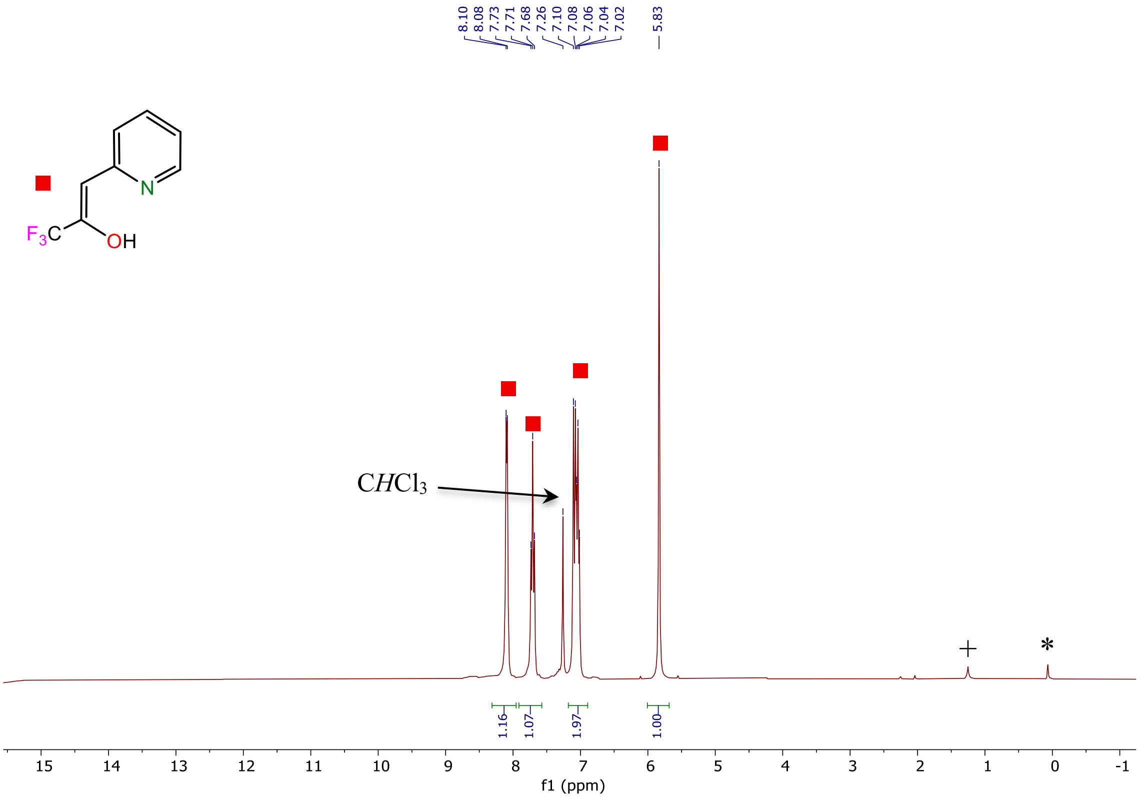

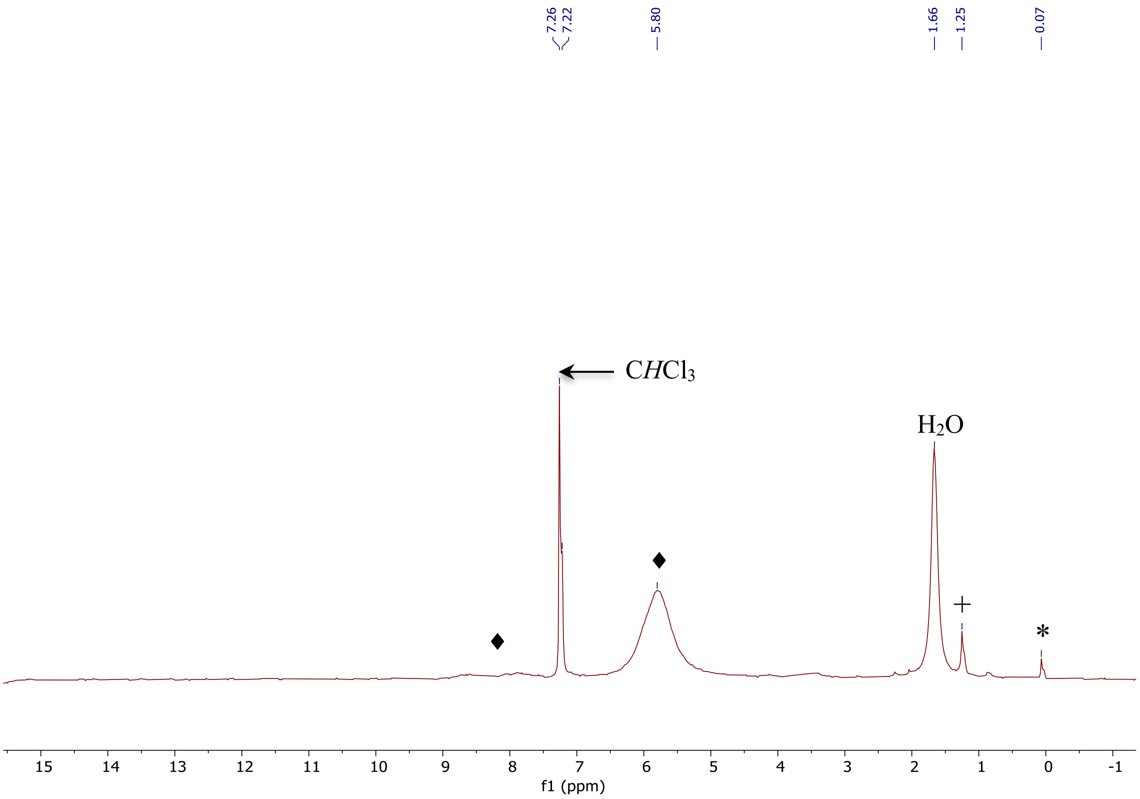

Figure S56. ^{1}H (300.1 MHz, 298 K) NMR spectra of **tfppOH** before (top) and after (bottom) the addition of 1 equivalent of HCl (■ = tfppOH ♦ = unknown new species, + = paraffin grease, * = impurities).

Table S1. Data extracted from the stability studies of **1_E** (in the presence of an internal standard)

HT	Stability test	Time (days)	NMR spectrum source	Fluorene CH_2 signal integral	HT signal[a] integral	Remaining HT %[b]	tfppOH signal[a] integral	tfppOH formed %[b]
**1_{Ge}**	Atmospheric air in $CDCl_3$ solution	0	Figure S35(top)	1	0,95	100,0	0,00	0,0
		1	Figure S35(bottom)	1	0,03	3,2	0,57	60,0
		3	Figure S36(top)	1	0,00	0,0	0,47	49,5
		7	Figure S36(bottom)	1	0,00	0,0	0,49	51,6
	Dry air in $CDCl_3$ solution	0	Figure S37(top)	1	1,02	100,0	0,00	0,0
		1	Figure S37(bottom)	1	1,02	100,0	0,00	0,0
		4	Figure S38(top)	1	0,85	83,3	0,08	7,8
		7	Figure S38(bottom)	1	0,81	79,4	0,11	10,8
	Atmospheric air as solid	1	Figure S39	1	0,75	75,0[c]	0,08	8,0[c]
**1_{Sn}**	Atmospheric air in $CDCl_3$ solution	0	Figure S40(top)	1	0,91	100,0	0,00	0,0
		1	Figure S40(bottom)	1	0,51[d]	56,0	0,29[d]	31,9
		3	Figure S41(top)	1	0,25[d]	27,5	0,57[d]	62,6
		7	Figure S41(bottom)	1	0,00	0,0	0,78	85,7
	Dry air in $CDCl_3$ solution	0	Figure S42(top)	1	0,91	100,0	0,00	0,0
		1	Figure S42(bottom)	1	0,71[d]	78,0	0,06[d]	6,6
		4	Figure S43(top)	1	0,35[d]	38,5	0,20[d]	22,0
		7	Figure S43(bottom)	1	0,12[d]	13,2	0,22[d]	24,2
	Atmospheric air as solid	1	Figure S44	1	0,76[d]	76,0[c]	0,07[d]	7,0[c]

[a]Vinylic C–H signal. [b]Calculated from the initial amount of HT present in solution according to the initial internal standard/HT ratio. [c]Calculated from the initial amount of HT added. [d]More deshielded pyridine ring C–H signal.

Table S2. Data extracted from the stability studies of **2_E** (in the presence of an internal standard)

HT	Stability test	Time (days)	NMR spectrum source	Fluorene CH_2 signal integral	HT signal[a] integral	Remaining HT %[b]	tfppOH signal[a] integral	tfppOH formed %[b]
**2_{Ge}**	Atmospheric air in $CDCl_3$ solution	0	Figure S45(top)	2	0,85	100,0	0,00	0,0
		1	Figure S45(bottom)	2	0,54	63,5	0,19	22,4
		3	Figure S46(top)	2	0,27	31,8	0,20	23,5
		7	Figure S46(bottom)	2	0,06	7,1	0,09	10,6
	Dry air in $CDCl_3$ solution	0	Figure S47(top)	2	0,93	100,0	0,00	0,0
		1	Figure S47(bottom)	2	0,92	98,9	0,03	3,2
		4	Figure S48(top)	2	0,80	86,0	0,04	4,3
		7	Figure S48(bottom)	2	0,77	82,8	0,11	11,8
	Atmospheric air as solid	1	Figure S49	2	0,31	31,0[c]	0,16	16,0[c]
**2_{Sn}**	Atmospheric air in $CDCl_3$ solution	0	Figure S50(top)	2	0,21	21,0[c]	0,00	0,0[c]
		1	Figure S50(bottom)	2	0,19	19,0[c]	0,33	33,0[c]
		3	Figure S51(top)	2	0,18[d]	18,0[c]	0,54[e]	54,0[c]
		7	Figure S51(bottom)	2	0,00	0,0[c]	0,75	75,0[c]
	Dry air in $CDCl_3$ solution	0	Figure S52(top)	2	0,21	21,0[c]	0,00	0,0[c]
		1	Figure S52(bottom)	2	0,17	17,0[c]	0,09	9,0[c]
		4	Figure S53(top)	2	0,18	18,0[c]	0,11	11,0[c]
		7	Figure S53(bottom)	2	0,12	12,0[c]	0,15	15,0[c]
	Atmospheric air as solid	1	Figure S54	2	0,27	27,0[c]	0,38	38,0[c]

[a]Vinylic C–H signal. [b]Calculated from the initial amount of HT present in solution according to the initial internal standard/HT ratio. [c]Calculated from the initial amount of HT added. [d]Vinylic C–H signal – tfppOH O–H signal integrals. [e]tfppOH O–H signal.

5. Stability Studies of the metal complexes

Air-stability of $\mathbf{3_{Ge}}$: An orange $CDCl_3$ solution of $\mathbf{3_{Ge}}$ was prepared in an NMR tube inside the glove box by adding $\mathbf{3_{Ge}}$ (17 mg; 0.05 mmol) to *ca*. 0.3 mL of $CDCl_3$. Some orange solid, remained undissolved. After acquiring the ^1H NMR spectrum under argon, the NMR tube was opened and left to stand under air for one day. A new ^1H NMR spectrum was then acquired (Figure S57). The air exposure led to the precipitation of additional orange crystalline material (possibly $\mathbf{3_{Ge}}$, due to solvent evaporation, among other species).

Air-stability of $\mathbf{5_{Ge}}$: Pale yellow solutions of $\mathbf{5_{Ge}}$ in C_6D_6 and $CDCl_3$ were prepared in NMR tubes inside the glove box by adding $\mathbf{5_{Ge}}$ (10 mg, 0.015 mmol) to *ca*. 0.3 mL of the corresponding solvent. Some purple (C_6D_6) and brown ($CDCl_3$) solid was observed. After acquiring their ^1H NMR spectra under argon, the NMR tubes were opened and left to stand under air for one day. New ^1H NMR spectra were then acquired (Figures S58 and S59). The air exposure led to the precipitation of additional amount of brownish solid (higher amount for the $CDCl_3$ solution).

Air-stability of $\mathbf{6_{Ge}}$: Pale yellow solutions of $\mathbf{6_{Ge}}$ in C_6D_6 and $CDCl_3$ were prepared in NMR tubes inside the glove box by adding $\mathbf{6_{Ge}}$ (10 mg, 0.009 mmol) to *ca*. 0.3 mL of the corresponding solvent. Some brown solid was observed. After acquiring their ^1H NMR spectra under argon, the NMR tubes were opened and left to stand under air for one day. New ^1H NMR spectra were then acquired (Figures S60 and S61). The air exposure led to the precipitation of an additional amount of brownish solid (higher amount for the $CDCl_3$ solution).

Air-stability of $\mathbf{7_{Ge}}$: A pale yellow THF-d_8 solution of $\mathbf{7_{Ge}}$ was prepared in an NMR tube inside the glove box by adding $\mathbf{7_{Ge}}$ (10 mg, 0.009 mmol) to *ca*. 0.3 mL of THF-d_8. Some white solid, remained undissolved. After acquiring the ^1H NMR spectrum under argon, the NMR tube was opened and left to stand protected from light under air for one day. A new ^1H NMR spectrum was then acquired (Figure S62). The air exposure led to no visual changes.

Air-stability of $\mathbf{7_{Sn}}$: An intense yellow THF-d_8 solution of $\mathbf{7_{Sn}}$ was prepared in an NMR tube inside the glove box by adding $\mathbf{7_{Sn}}$ (10 mg, 0.008 mmol) to *ca*. 0.3 mL of THF-d_8. After acquiring the ^1H NMR spectrum under argon, the NMR tube was opened and left to stand protected from light under air for one day. A new ^1H NMR spectrum was then

acquired (Figure S63). The air exposure led to the formation of a layer of a metallic grey solid on the wall of the NMR tube.

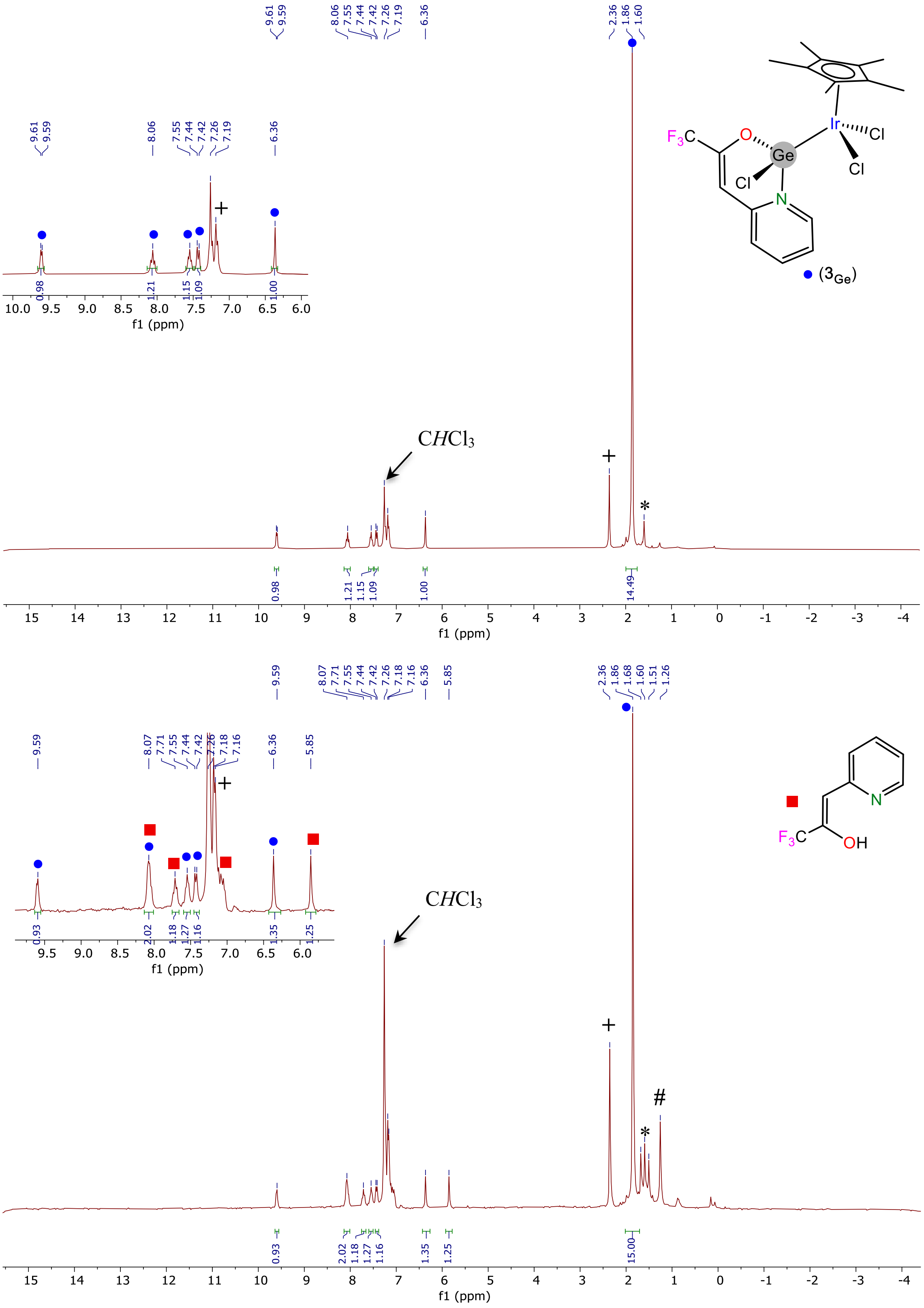

Figure S57. ^{1}H (top, 300.1 MHz) NMR spectra (CDCl$_3$, 298 K) of **3$_{Ge}$** under argon (top) and after standing in air for one day (bottom). (● = **3$_{Ge}$**, ■ = tfppOH, + = toluene, # = paraffin grease, * = impurities).

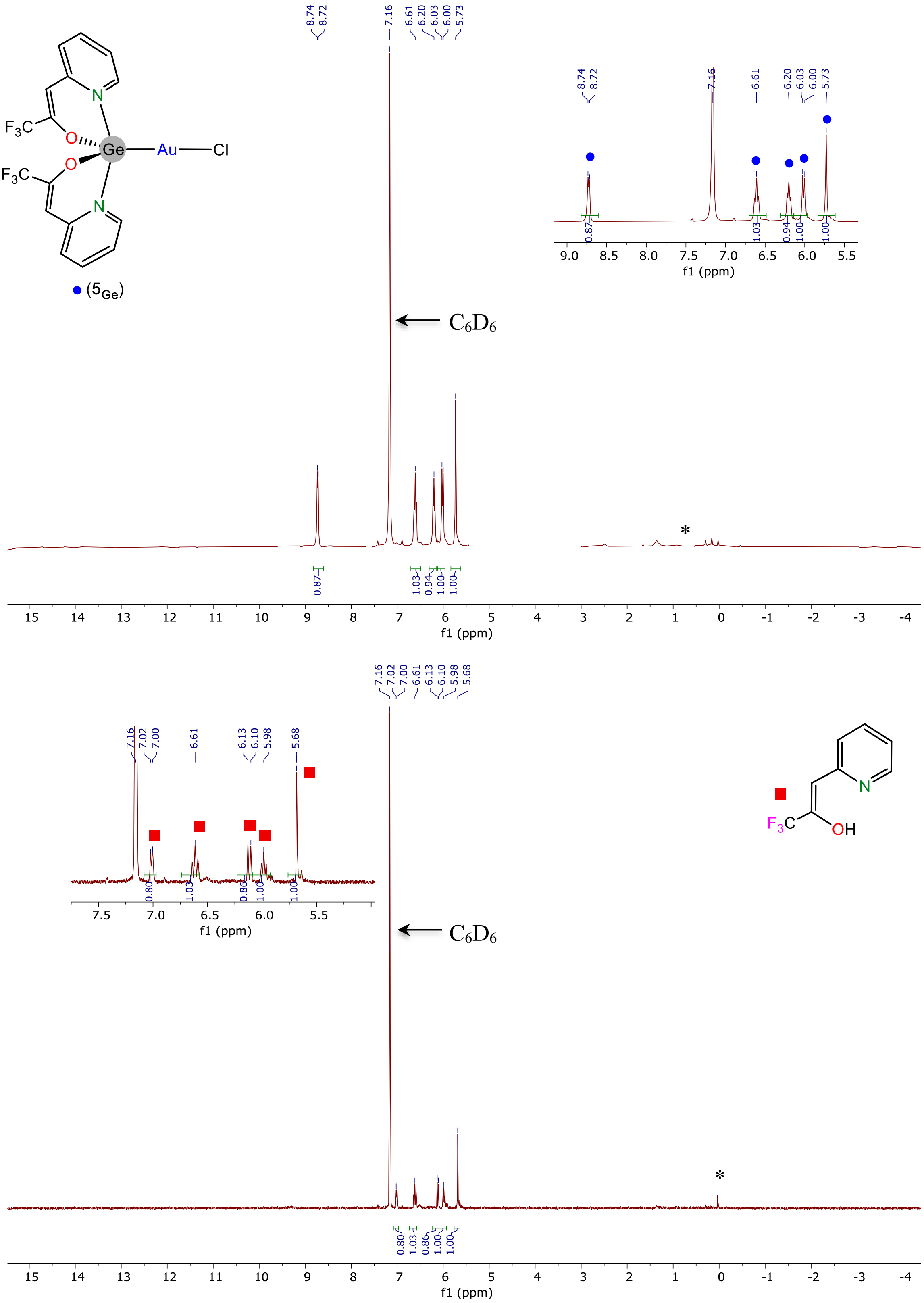

Figure S58. ^{1}H (top, 300.1 MHz) NMR spectra (C_6D_6, 298 K) of $\mathbf{5_{Ge}}$ under argon (top) and after standing in air for one day (bottom). (● = $\mathbf{5_{Ge}}$, ■ = tfppOH, * = impurities).

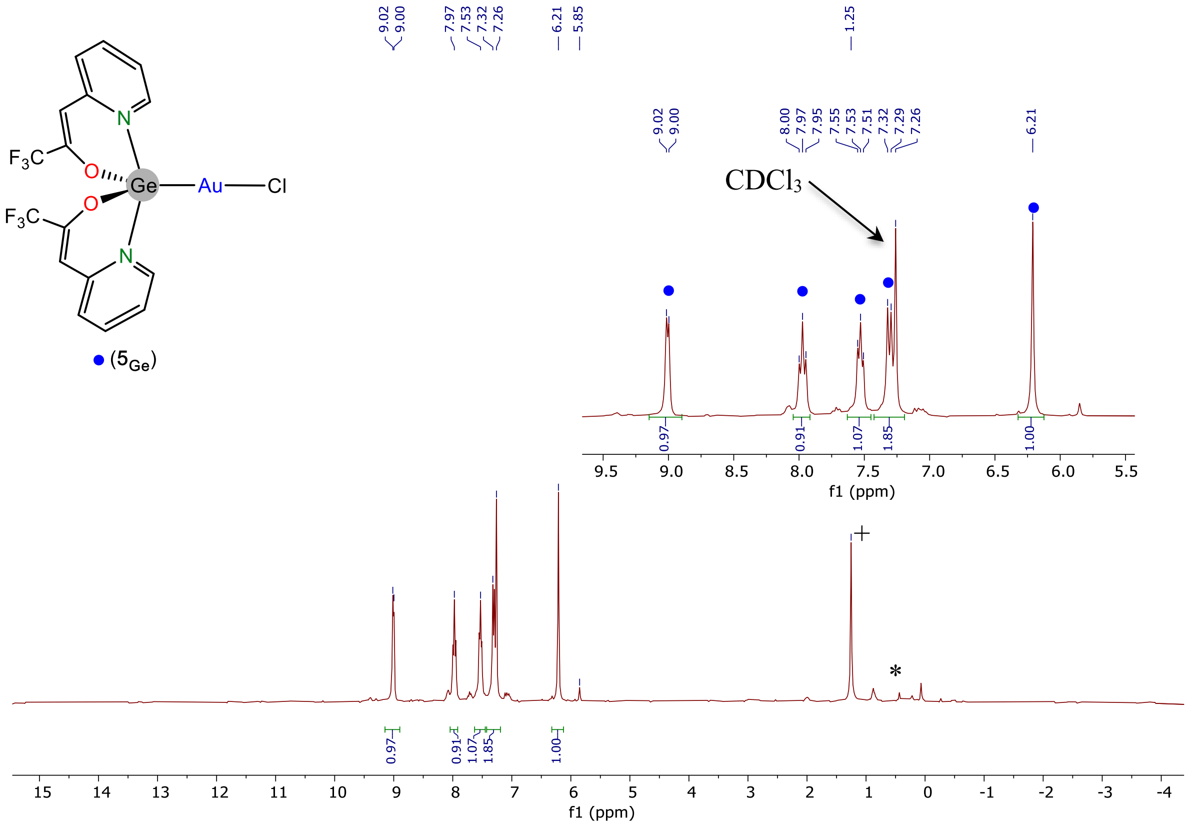

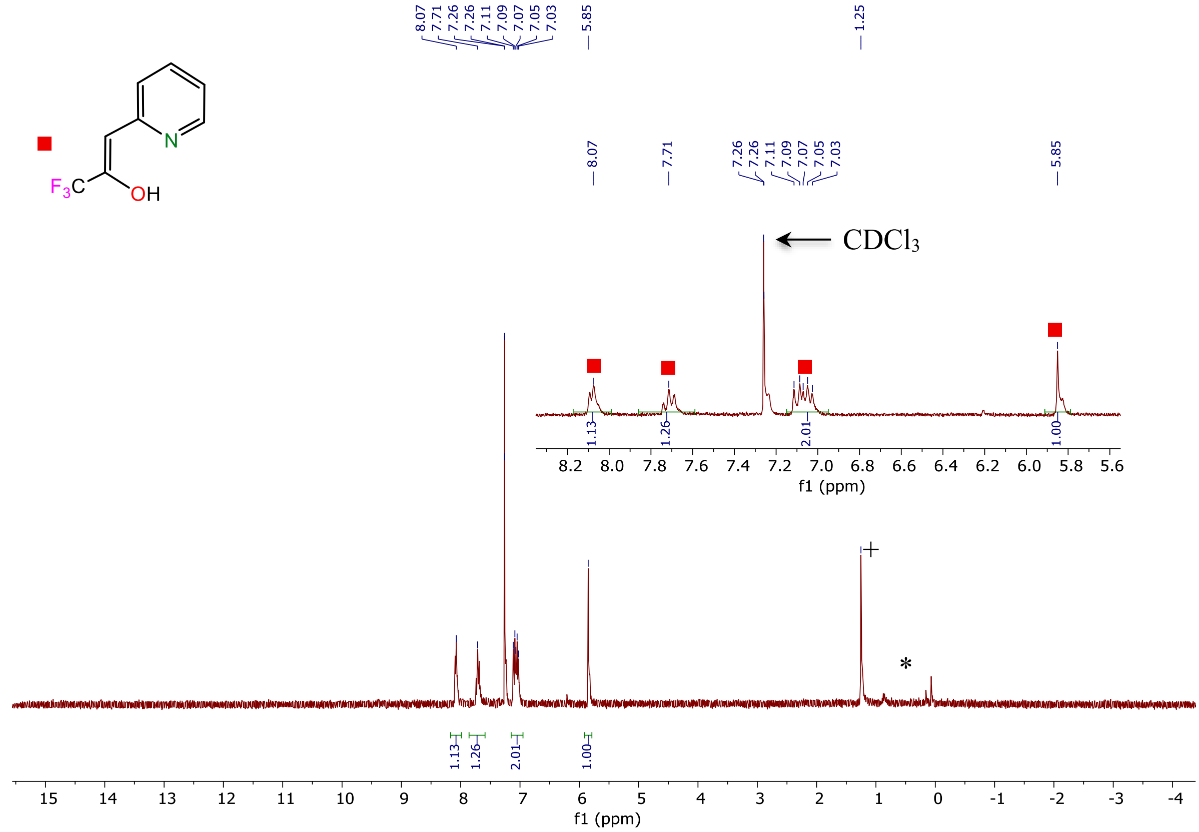

Figure S59. ^{1}H (top, 300.1 MHz) NMR spectra ($CDCl_3$, 298 K) of $\mathbf{5_{Ge}}$ under argon (top) and after standing in air for one day (bottom). (● = $\mathbf{5_{Ge}}$, ■ = tfppOH, + = paraffin grease, * = impurities).

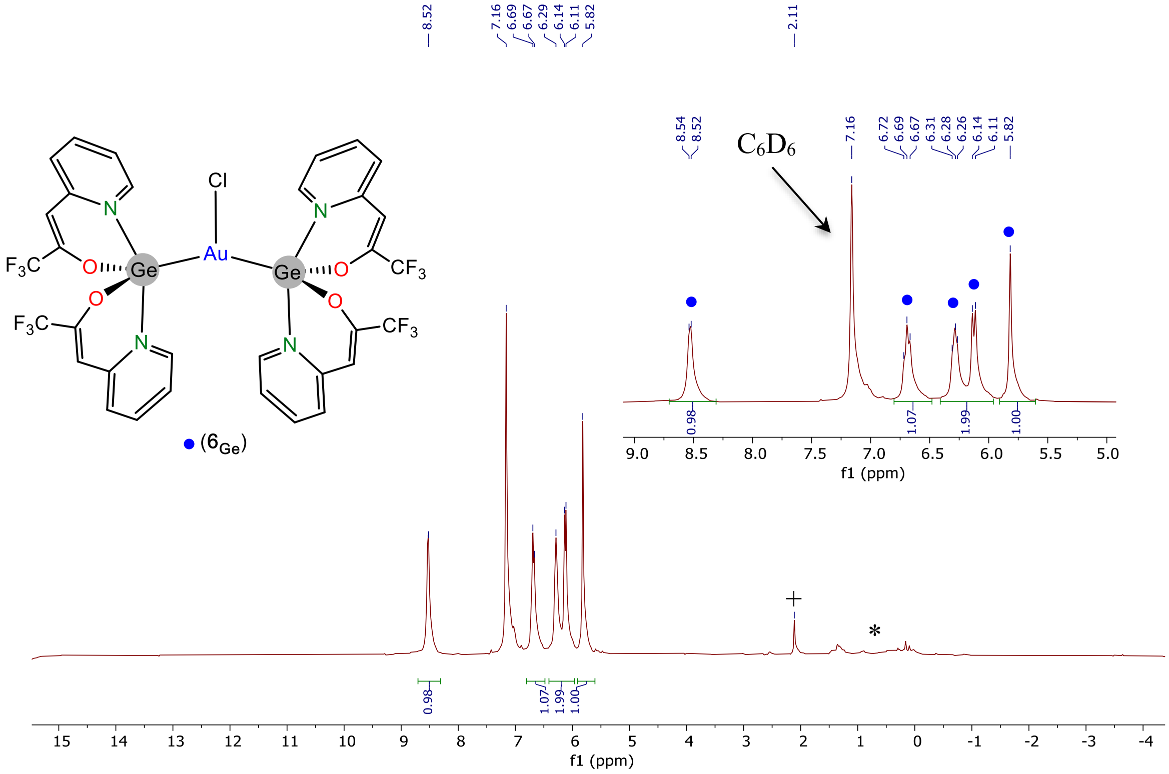

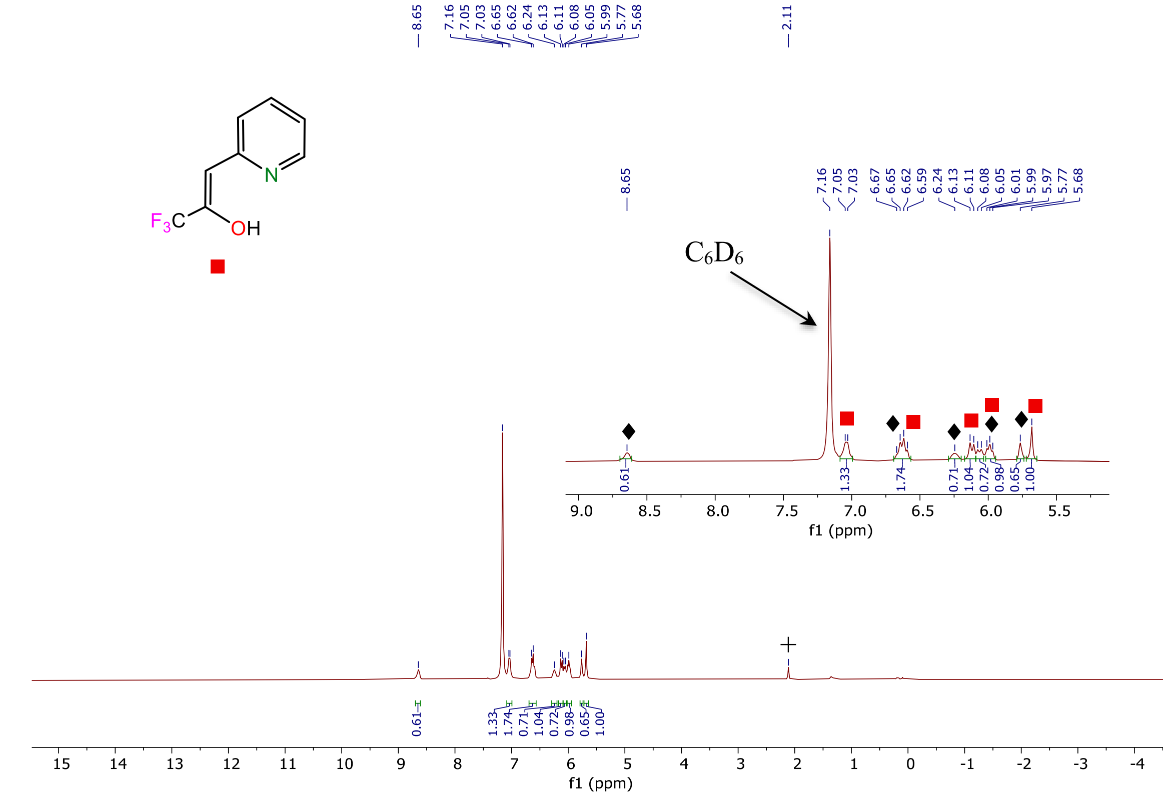

Figure S60. ^{1}H (top, 300 MHz) NMR spectra (C_6D_6, 298 K) of $\mathbf{6_{Ge}}$ under argon (top) and after standing in air for one day (bottom). (● = $\mathbf{6_{Ge}}$, ■ = tfppOH, ♦ = $\mathbf{5_{Ge}}$, + = toluene, * = impurities,).

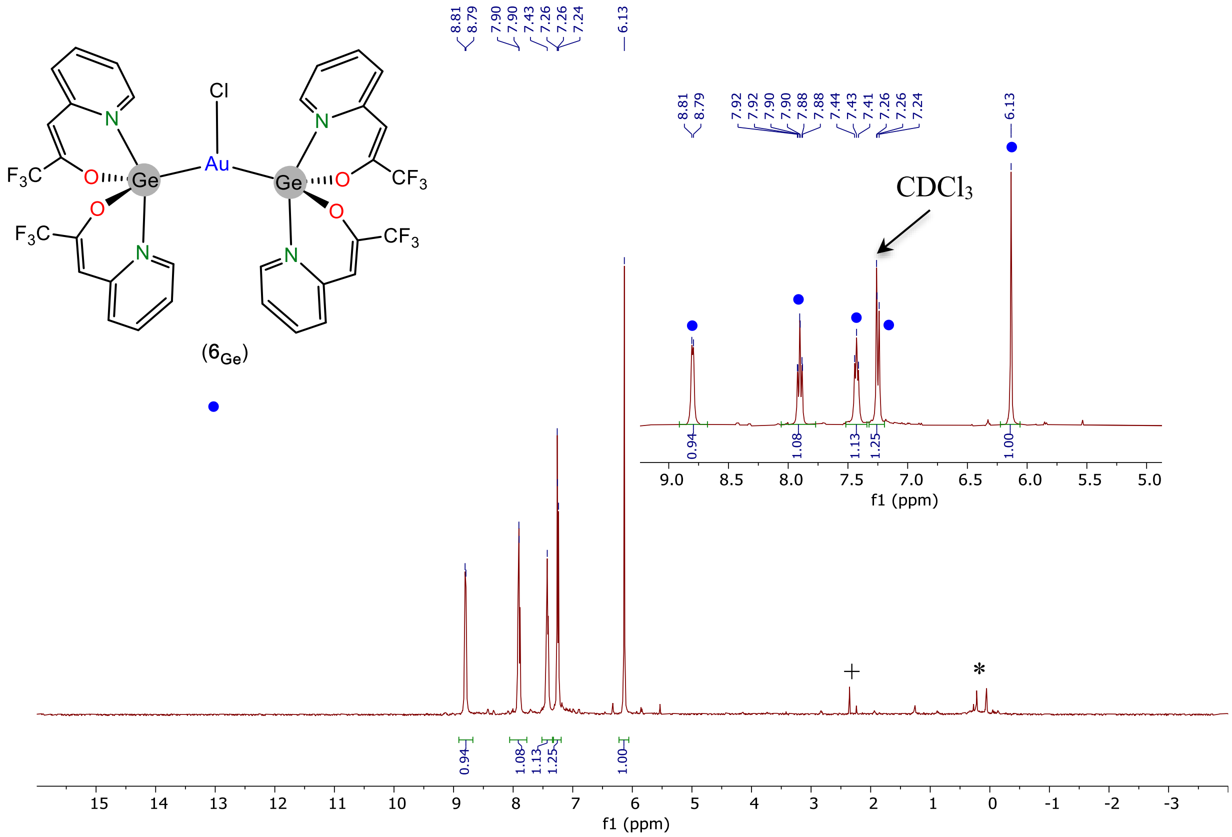

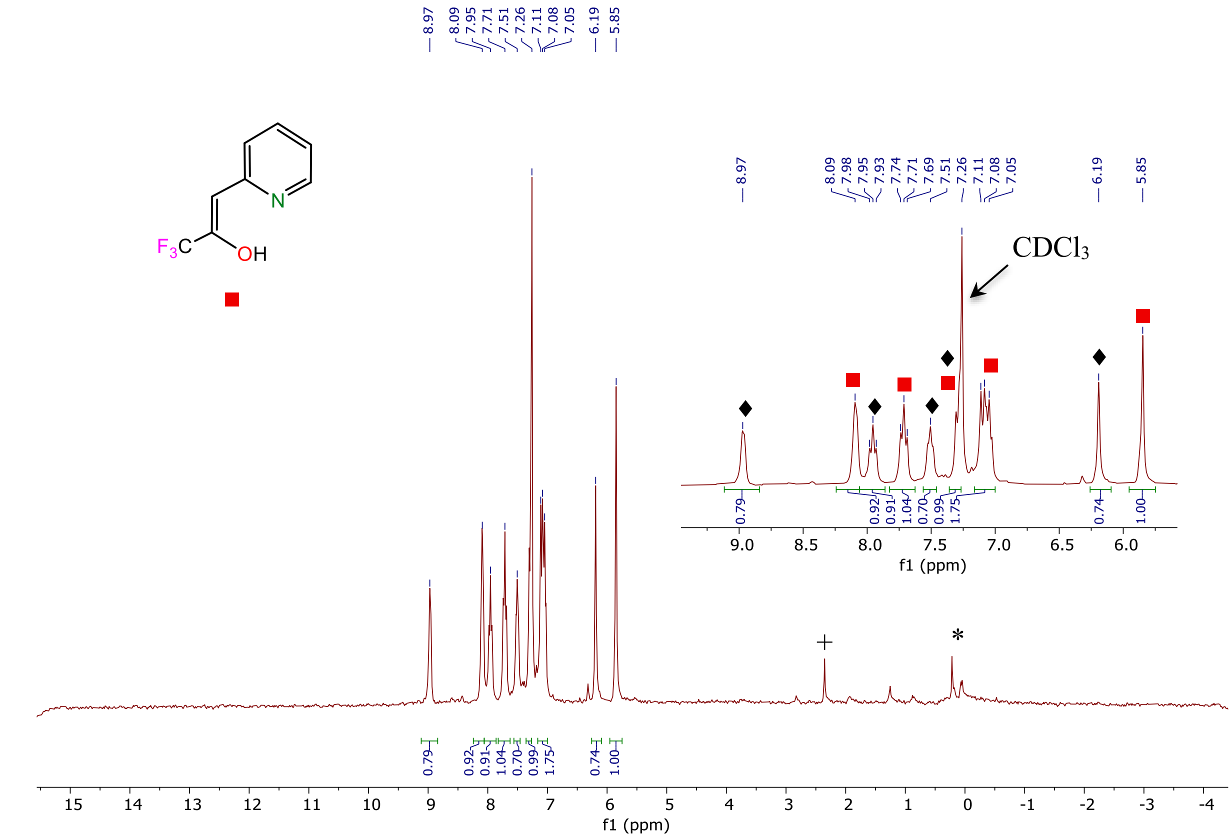

Figure S61. ^{1}H (top, 300.1 MHz) NMR spectra ($CDCl_3$, 298 K) of $\mathbf{6_{Ge}}$ under argon (top) and after standing in air for one day (bottom). (● = $\mathbf{6_{Ge}}$, ■ = tfppOH, ♦ = $\mathbf{5_{Ge}}$, + = toluene, * = impurities).

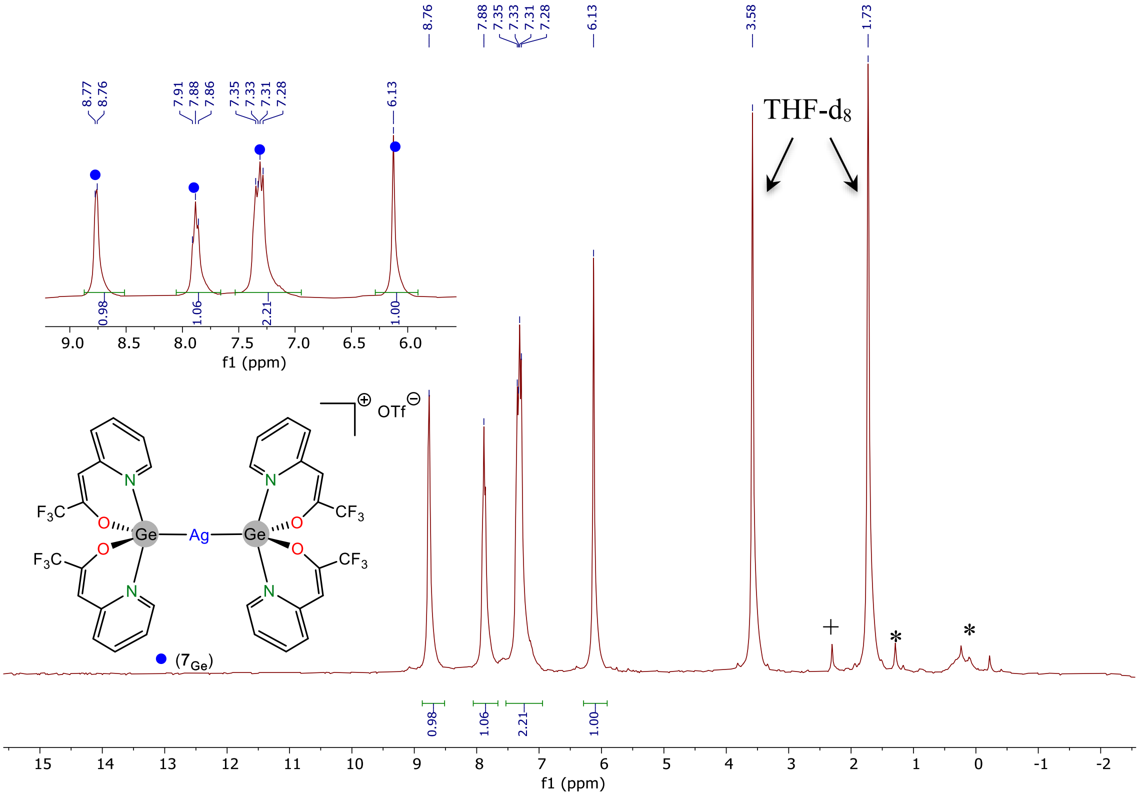

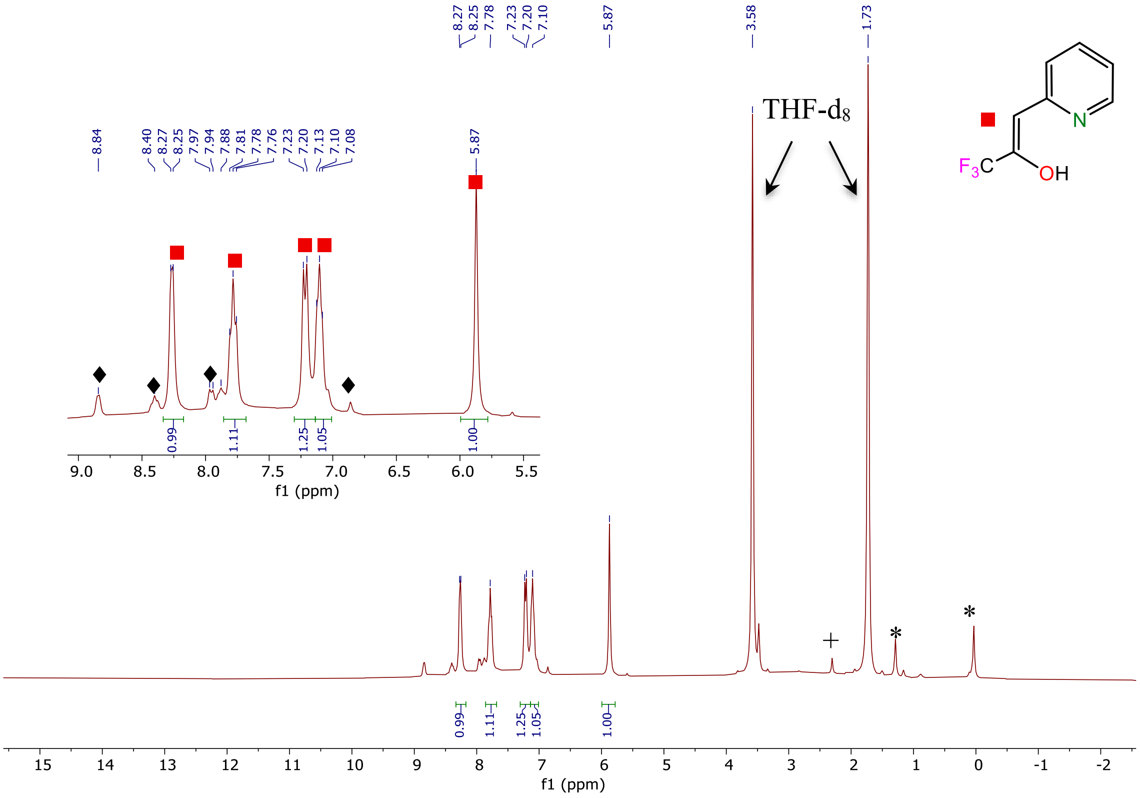

Figure S62. ^{1}H (top, 300.1 MHz) NMR spectra (THF-d$_8$, 298 K) of **7$_{Ge}$** under argon (top) and after standing in air for one day (bottom). (● = **7$_{Ge}$**, ■ = tfppOH), ♦ = unknown new species, + = toluene, * = impurities).

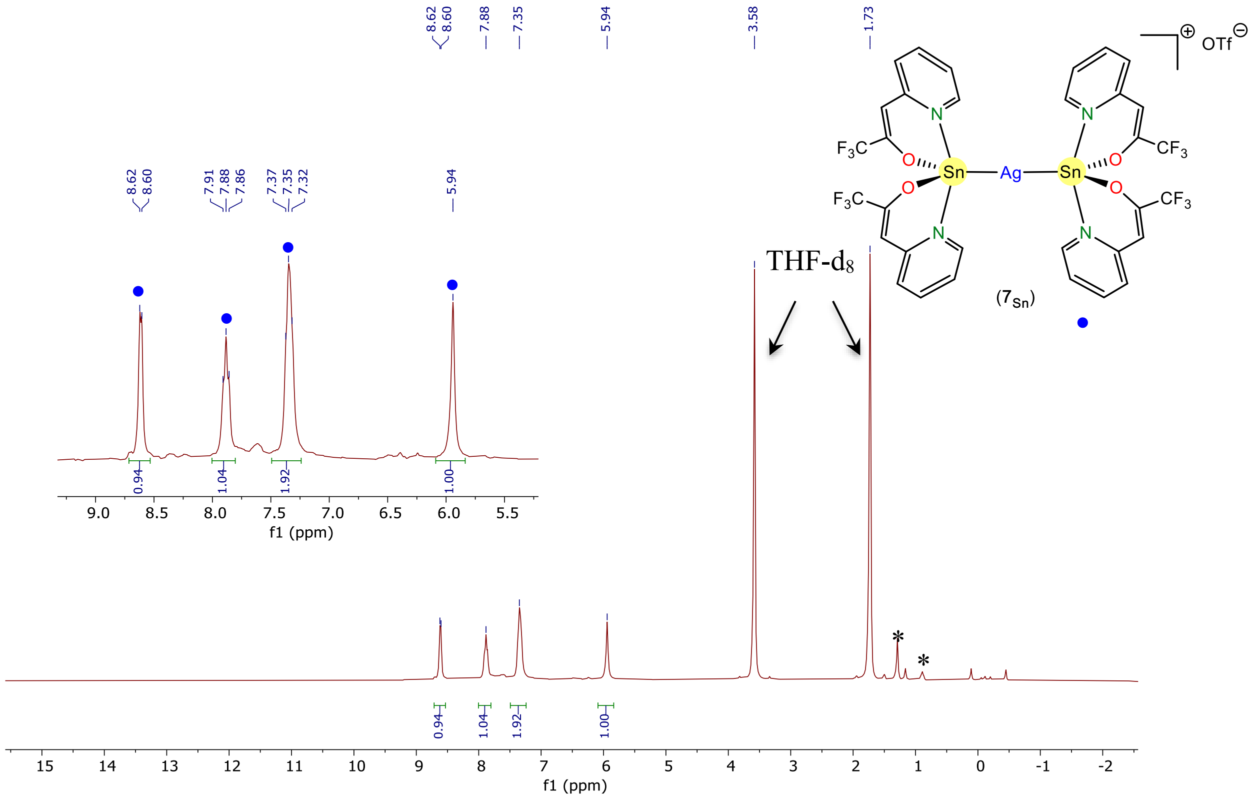

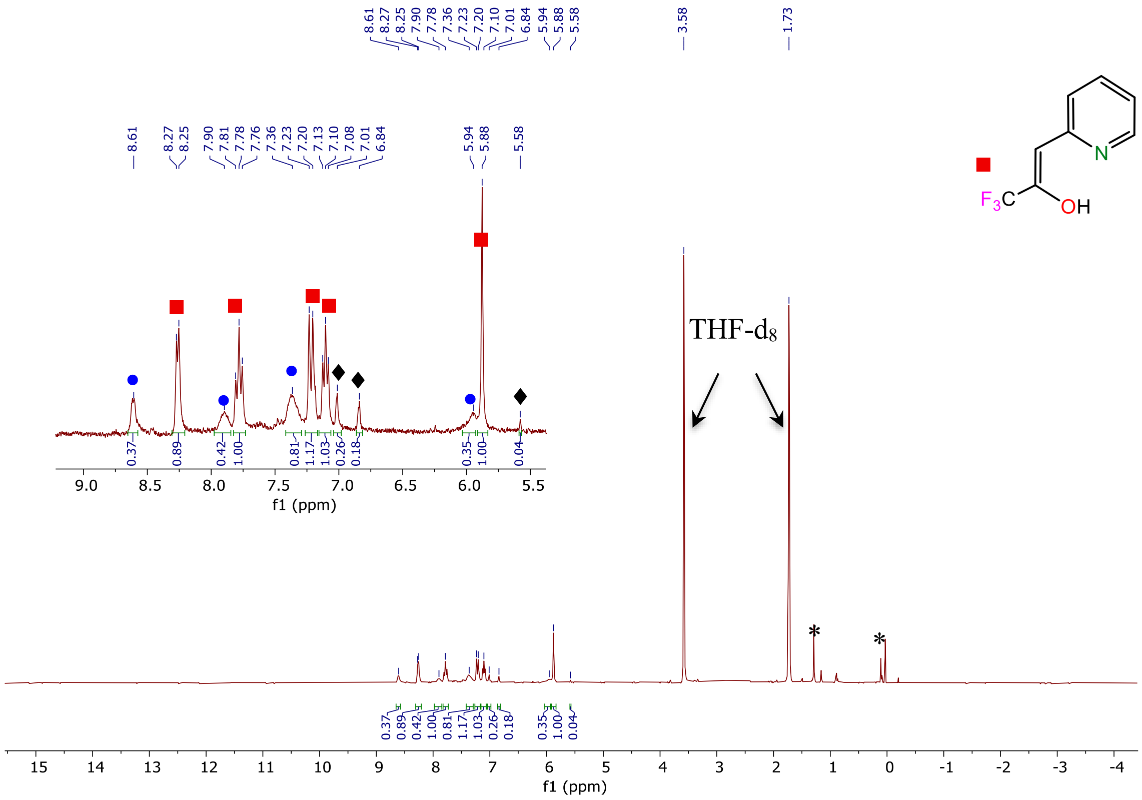

Figure S63. ^{1}H (top, 300.1 MHz) NMR spectra (THF-d$_8$, 298 K) of **7$_{Sn}$** under argon (top) and after standing in air for one day (bottom). (● = **7$_{Sn}$**, ■ = tfppOH), ♦ = unknown new species, * = impurities).

6. X-ray Diffraction Data

Crystals of **2**$_{Ge}$, **2**$_{Sn}$, **3**$_{Ge}$ and **5**$_{Ge}$ were analyzed by X-ray diffraction. A selection of crystal, measurement and refinement data is given in Table S3. Diffraction data were collected on a Bruker APEX II CCD (with MoKα radiation for **2**$_{Ge}$ and **2**$_{Sn}$) and Oxford Diffraction Xcalibur Onyx Nova Geminy (with CuKα radiation for **3**$_{Ge}$ and **5**$_{Ge}$) single crystal diffractometers. Empirical absorption corrections were applied using SADABS-2016/2[S6] (for **2**$_{Ge}$ and **2**$_{Sn}$) and the SCALE3 ABSPACK algorithm as implemented in Chrysalis RED[S7] (for **3**$_{Ge}$ ·and **5**$_{Ge}$). The structures were solved with SIR-92.[S8] Isotropic and full matrix anisotropic least square refinements were carried out using SHELXL.[S9] The WINGX program system[S10] was used throughout the structure determinations. The molecular plots were made with MERCURY.[S11]

Table S3. Crystal, measurement and refinement data for the compounds studied by X-ray diffraction.

	**2**$_{Ge}$	**2**$_{Sn}$	**3**$_{Ge}$	**5**$_{Ge}$
formula	$(C_8H_5ClF_3GeNO)_2$	$C_8H_5ClF_3NOSn$	$(C_{18}H_{20}Cl_3F_3GeIrNO)_2$	$C_{16}H_{10}AuClF_6GeN_2O_2$
fw	592.34	342.27	1388.98	1362.53
cryst syst	monoclinic	triclinic	triclinic	triclinic
space group	*P*21/c	*P*–1	*P*–1	*P*–1
a, Å	27.679(2)	6.7820(3)	8.4563(3)	8.1592(3)
b, Å	5.0863(3)	8.8796(5)	14.3665(5)	10.3364(4)
c, Å	14.4514(9)	9.9418(5)	19.0670(5)	12.5670(5)
α, deg	90	112.868(2)	96.849(2)	96.119(3)
β, deg	104.605(2)	97.897(2)	95.476(2)	99.383(3)
γ, deg	90	107.704(2)	103.968(3)	110.401(3)
V, Å^3	1968.8(2)	502.76(4)	2213.2(1)	964.66(7)
Z	4	2	2	2
F(000)	1152	324	1320	1496
D_{calcd}, g cm^{-3}	1.998	2.261	2.084	2.345
μ, mm^{-1}	3.397 (MoKα)	2.820 (MoKα)	16.792 (CuKα)	17.979 (CuKα)
cryst size, mm	0.19 x 0.04 x 0.02	0.13 x 0.06 x 0.03	0.58 x 0.25 x 0.04	0.59 x 0.32 x 0.10
T, K	100(2)	100(2)	150(2)	150(2)
θ range, deg	2.28 to 28.28	2.32 to 28.27	3.21 to 69.77	3.62 to 69.48
min./max. *h*, *k*, *l*	–36/36, –6/6, –19/19	–9/9, –11/11, –13/13	–10/8, –17/17, –22/23	–9/8, –12/12, –14/15
no. collected reflns	98994	17916	21798	13265
no. unique reflns	4885	2492	8210	3579
no. reflns with $I > 2\sigma(I)$	4175	2331	7274	3428
no. params/restraints	271/0	136/0	515/0	262/0
GOF (on F^2)	1.193	1.050	1.042	1.068
R_1 (on F, $I > 2\sigma(I)$)	0.036	0.018	0.044	0.044
wR_2 (on F^2, all data)	0.077	0.045	0.127	0.123
min./max. $\Delta\rho$, e Å^{-3}	–0.600/0.728	–0.405/0.785	–2.419/1.531	–2.015/2.490
CCDC dep. no.	2516399	2516400	2516401	2516402

7. Theoretical Calculations

Structure optimizations were performed with the Gaussian09 suite of programs,[S12] using the BP86 functional,[S13] with the D3 dispersion correction suggested by Grimme et al.[S14] The Stuttgart-Dresden relativistic effective core potentials and the associated basis sets (SDD) were used for the Ge, Sn and Au atoms.[S15] The basis set used for the remaining atoms was the cc-pVDZ.[S16] Frequency calculations confirmed the optimized structures as energy minima (zero imaginary eigenvalues) or as transition states (one imaginary eigenvalue). Gibbs energies were computed at 298.15 K and 1.0 atm. Solvation free energies were obtained with the self consistent reaction field (SCRF) for the standard continuum solvation model (CPCM),[S17] by using the single-point solvation energy of the optimized structures and the thermodynamic correction from the gas phase calculations.

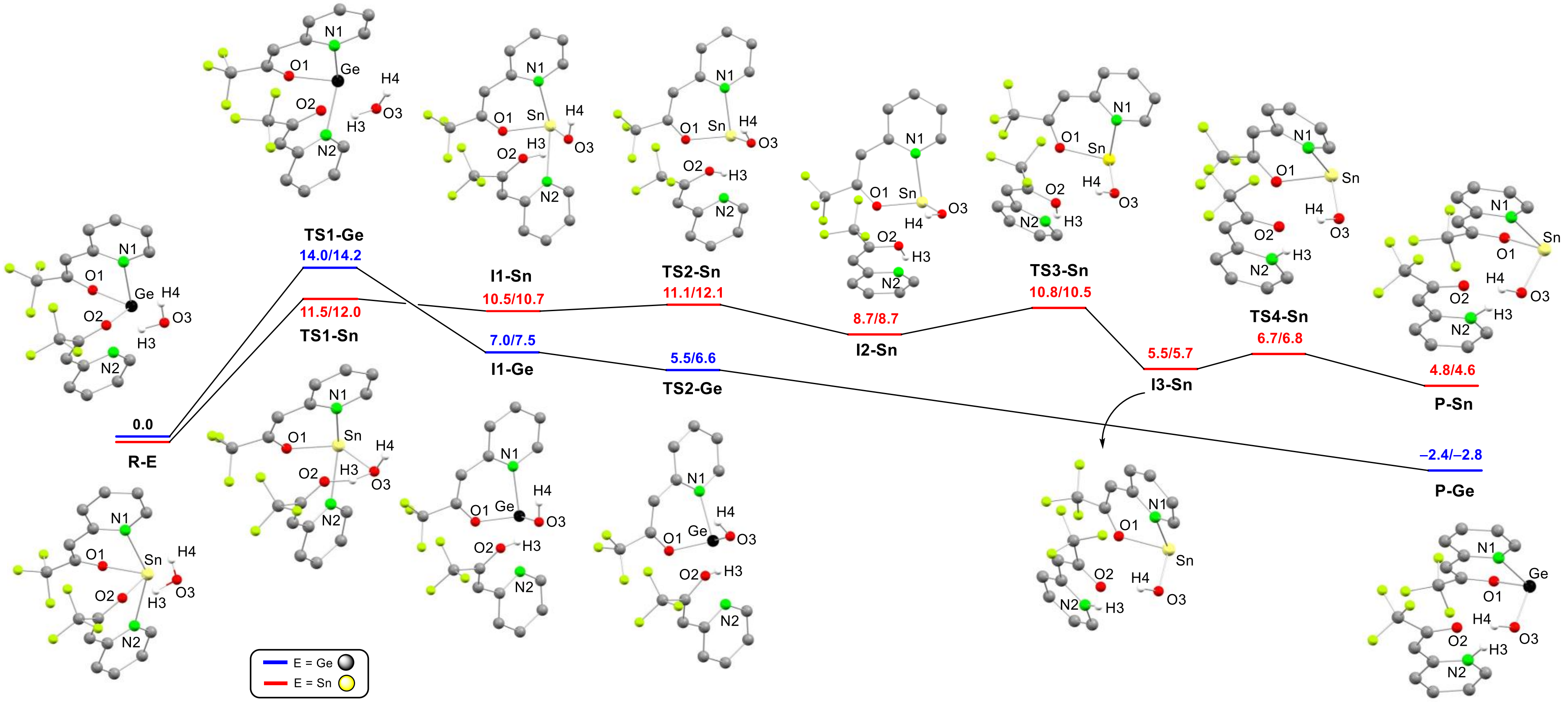

Figure S64. DFT-optimized structures (BP86-D3/SDD(Ge,Sn)) of the stationary points and relative energy profiles of the transformations of $\mathbf{1_E}$ + H_2O (**R-E**) into E(tfppO)OH + tfppOH (**P-E**), for E = Ge and Sn. For clarity, (a) only the H atoms derived from the water molecule are shown. Gibbs energies (CPCM-$CDCl_3/C_6D_6$, kcal mol^{-1}) are relative to those of **R-E**.

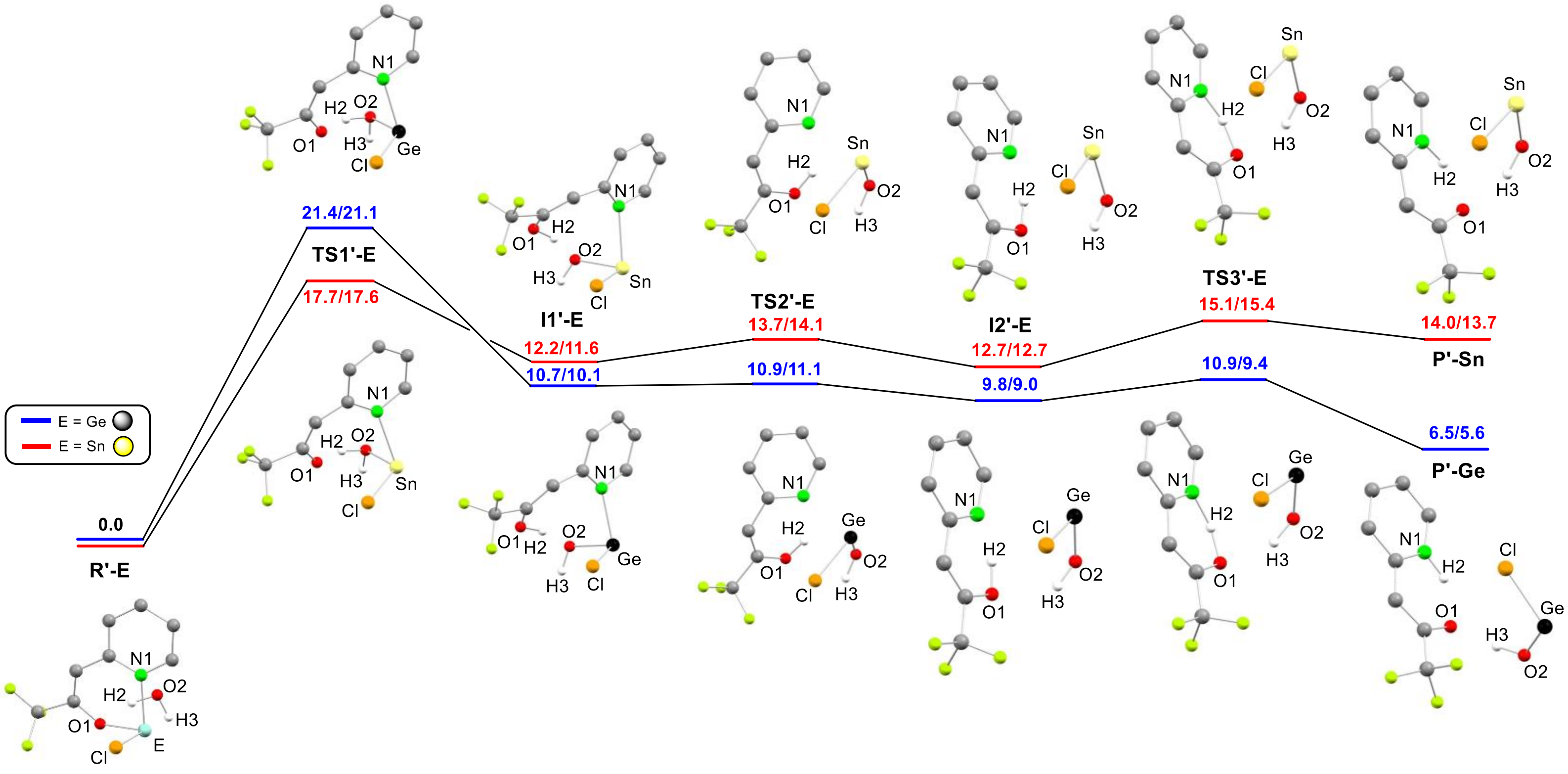

Figure S65. DFT-optimized structures (BP86-D3/SDD(Ge,Sn)) of the stationary points and relative energy profiles of the first steps of the transformations of $\mathbf{2_E}$ + H_2O (**R'-E**) into ECl(OH) + tfppOH (**P'-E**), for E = Ge and Sn. For clarity, only the H atoms derived from the water molecule are shown. Gibbs energies (CPCM-$CDCl_3$/C_6D_6, kcal mol^{-1}) are relative to those of **R'-E**.

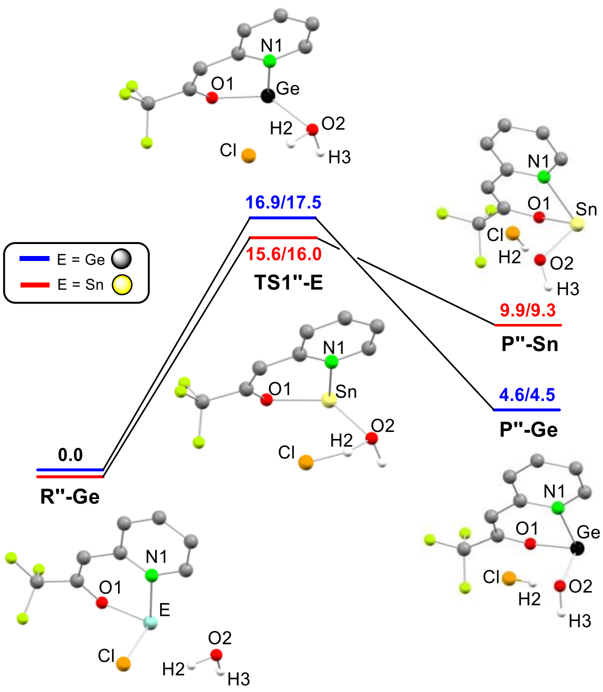

Figure S66. DFT-optimized structures (BP86-D3/SDD(Ge,Sn)) of the stationary points and relative energy profiles of the first steps of the transformations of $\mathbf{2}_{\mathbf{E}}$ + H_2O (**R''-E**) into E(tfppO)(OH) + HCl (**P''-E**), for E = Ge and Sn. For clarity, only the H atoms derived from the water molecule are shown. Gibbs energies (CPCM-$CDCl_3$/C_6D_6), kcal mol^{-1}) are relative to those of **R''-E**.

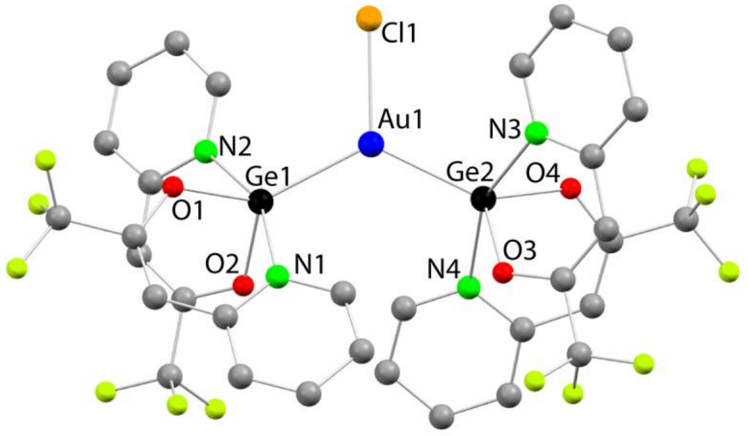

Figure S67. DFT-optimized structure of **6$_{AuClGe2}$** (H atoms have been omitted for clarity except that on the carbene carbon atom). Selected bond lengths (Å) and angles (°): Au1−Cl1 2.464, Au−Ge1 2.436, Au−Ge2 2.436, Ge1−N1 2.223, Ge1−N2 2.226, Ge1−O1 1.910, Ge1−O2 1.925, Ge2−N3 2.226, Ge2−N4 2.224, Ge2−O3 1.925, Ge2−O4 1.910; Ge1−Au1−Ge2 126.39, Ge1−Au1−Cl1 116.83, Ge2−Au1−Cl1 116.78.

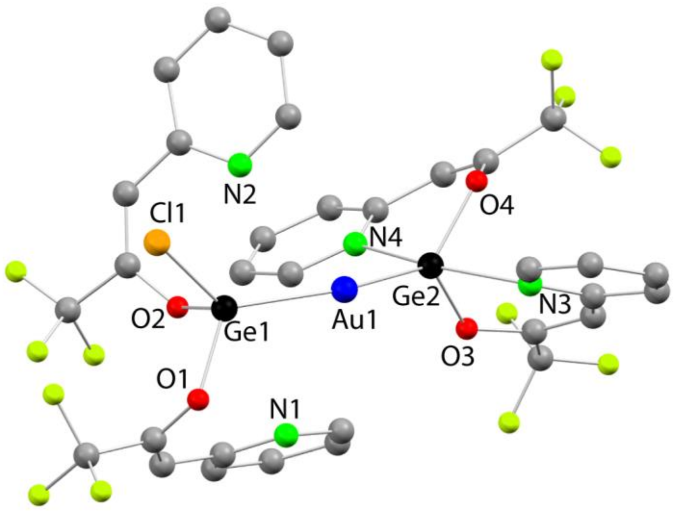

Figure S68. DFT-optimized structure of **6$_{AuGeClGe}$** (H atoms have been omitted for clarity except that on the carbene carbon atom). Selected bond lengths (Å) and angles (°): Au−Ge1 2.401, Au−Ge2 2.397, Au−Cl1 2.206, Ge1−N1 3.264, Ge1−N2 3.021, Ge1−O1 1.863, Ge1−O2 1.882, Ge2−N3 2.214, Ge2−N4 2.184, Ge2−O3 1.893, Ge2−O4 1.880; Ge1−Au1−Ge2 150.90.

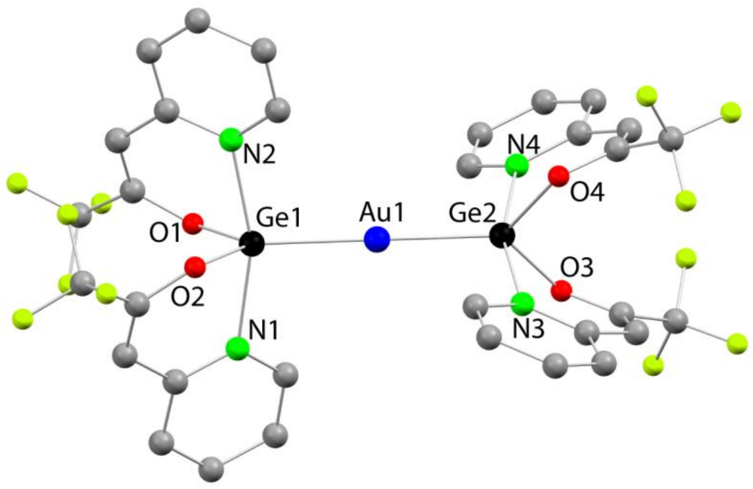

Figure S69. DFT-optimized structure of the cation of **$6_{AuGe2+Cl-}$** (H atoms have been omitted for clarity except that on the carbene carbon atom). Selected bond lengths (Å) and angles (°): Au−Ge1 2.422, Au−Ge2 2.422, Ge1−N1 2.176, Ge1−N2 2.176, Ge1−O1 1.867, Ge1−O2 1.867, Ge2−N3 2.176, Ge2−N4 2.176, Ge2−O3 1.867, Ge2−O4 1.867; Ge1−Au1−Ge2 179.94.

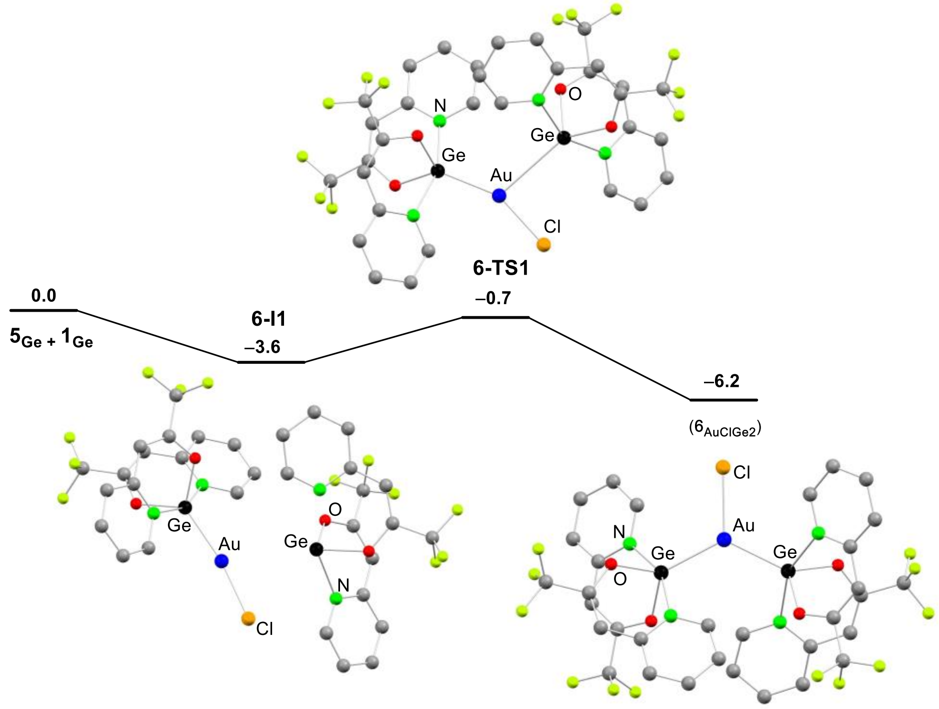

Figure S70. DFT-optimized structures (BP86-D3/SDD(Ge,Au)) and relative energy profiles of the stationary points involved in the transformations of **5Ge** + **1_{Ge}** into the isomer **$6_{AuClGe2}$**. For clarity, the hydrogen atoms have been omitted. The given energies are relative to that of **5Ge** + **1_{Ge}**. Gibbs energies (CPCM-toluene) are given in kcal mol^{-1}.

8. References

S1 Cabeza, J. A.; Reynes, J. F.; García, F.; García-Álvarez, P.; García-Soriano, R. Fast and scalable solvent-free access to Lappert's heavier tetrylenes E{N(SiMe3)2}2 (E = Ge, Sn, Pb) and ECl{N(SiMe3)2} (E = Ge, Sn). *Chem. Sci.* **2023**, *14*, 12477–12483.

S2 Heidemann, T.; Mathur, S. Air-Stable and Volatile Bis(pyridylalkenolato)germanium(II), -tin(II), and -lead(II) Complexes. *Eur. J. Inorg. Chem.* **2014**, 506–510.

S3 Kawase, M.; Teshima, M.; Saito, S.; Tani, S. Trifluoroacetylation of methylpyridines and other methylazines: A convenient access to trifluoroacetonylazines. Heterocycles **1998**, *48*, 2103–2108.

S4 Ball, R. G.; Graham, W. A. G.; Heinekey, D. M.; Hoyano, J. K.; McMaster, A. D.; Mattson, B. M.; Michel, S. T. Synthesis and structure of dicarbonylbis(.eta.-pentamethylcyclopentadienyl)diiridium. *Inorg. Chem.* **1990**. *29,* 2023–2025.

S5 Usón, R.; Laguna, A.; Laguna, M.; Briggs, D. A.; Murray, H. H.; Fackler, J. (Tetrahydrothiophene)Gold(I) or Gold(III) Complexes. *Inorg. Synth*. **1989**, *26*, 85–91.

S6 *SADABS-2016/2*: Krause, L.; Herbst-Irmer, R.; Sheldrick, G. M.; Stalke, D. Comparison of silver and molybdenum microfocus X-ray sources for single-crystal structure determination. *J. Appl. Crystallogr.* **2015**, *48*, 3–10.

S7 *CrysAlisPro RED*, version 1.171.38.46: Oxford Diffraction Ltd., Oxford, UK, 2015.

S8 *SIR-92*: Altomare, A.; Cascarano, G. Giacovazzo, C.; Guagliardi, A.; Burla, M. C.; Polidori, G.; Camalli, M. SIR92: a program for automatic solution of crystal structures by direct methods. *J. Appl. Crystallogr.* **1994**, *27*, 435–436.

S9 *SHELXL-2014*: Sheldrick, G. M. A short history of SHELX. *Acta Cryst.* **2008**, *A64*, 112–122.

S10 *WINGX*, version 2021.3: Farrugia, L. WinGX and ORTEP for Windows: an update. *J. Appl. Crystallogr.* **2012**, *45*, 849–854.

S11 *MERCURY*, version 2025.2.0 (build 454209): Cambridge Crystallographic Data Centre, Cambridge, UK, 2025.

S12 Frisch, M. J.; Trucks, G. W.; Schlegel, H. B.; Scuseria, G. E.; Robb, M. A.; Cheeseman, J. R.; Scalmani, G.; Barone, V.; Mennucci, B.; Petersson, G. A.; Nakatsuji, H.; Caricato, M.; Li, X.; Hratchian, H. P.; Izmaylov, A. F.; Bloino, J.; Zheng, G.; Sonnenberg, J. L.; Hada, M.; Ehara, M.; Toyota, K.; Fukuda, R.; Hasegawa, J.; Ishida, M.; Nakajima, T.; Honda, Y.; Kitao, O.; Nakai, H.; Vreven, T.; Montgomery, J. A., Jr.; Peralta, J. E.; Ogliaro, F.; Bearpark, M.; Heyd, J. J.; Brothers, E.; Kudin, K. N.; Staroverov, V. N.; Kobayashi, R.; Normand, J.; Raghavachari, K.; Rendell, A.; Burant, J. C.; Iyengar, S. S.; Tomasi, J.; Cossi, M.; Rega, N.; Millam, J. M.; Klene, M.; Knox, J. E.; Cross, J. B.; Bakken, V.; Adamo, C.; Jaramillo, J.; Gomperts, R.; Stratmann, R. E.; Yazyev, O.; Austin, A. J.; Cammi, R.; Pomelli, C.; Ochterski, J. W.; Martin, R. L.; Morokuma, K.; Zakrzewski, V. G.; Voth, G. A.; Salvador, P.; Dannenberg, J. J.; Dapprich, S.; Daniels, A. D.; Farkas, O.; Foresman, J. B.; Ortiz, J. V.; Cioslowski, J.; Fox, D. J. *Gaussian 09,* revision A.01; Gaussian, Inc.: Wallingford, CT, 2009.

S13 Weigend, F.; Ahlrichs, R. Balanced basis sets of split valence, triple zeta valence and quadruple zeta valence quality for H to Rn: Design and assessment of accuracy. *Phys. Chem. Chem. Phys*. **2005**, *7*, 3297–3305.

S14 Grimme, S.; Antony, J.; Ehrlich, S.; Krieg, H. A consistent and accurate ab initio parametrization of density functional dispersion correction (DFT-D) for the 94 elements H-Pu. *J. Chem. Phys*. **2010**, *132*, 154104.

S15 (a) Metz, B.; Stoll, H.; Dolg, M. Small-core multiconfiguration-Dirac–Hartree–Fock-adjusted pseudopotentials for post-d main group elements: Application to PbH and PbO. *J. Chem. Phys*. **2000**, *113*, 2563–2569. (b) Figgen, D.; Rauhut, G.; Dolg, M.; Stoll, H. Energy-consistent pseudopotentials for group 11 and 12 atoms: adjustment to multi-configuration Dirac–Hartree–Fock data. *Chem. Phys.* **2005**, 311, 227–244.

S16 Dunning, T. H. Gaussian basis sets for use in correlated molecular calculations. I. The atoms boron through neon and hydrogen. *J. Chem. Phys*. **1989**, *90*, 1007–1023.

S17 (a) Barone, V.; Cossi, M. Quantum Calculation of Molecular Energies and Energy Gradients in Solution by a Conductor Solvent Model. *J. Phys. Chem. A* **1998**, *102*, 1995−2001. (b) Cossi, M.; Rega, N.; Scalmani, G.; Barone, V. Energies, structures,

and electronic properties of molecules in solution with the C-PCM solvation model. *J. Comput. Chem.* **2003**, *24*, 669−681.
